# Supplementary material for: Correlates of and changes in aerobic physical activity and strength training before and after the onset of COVID-19 pandemic in the UK: findings from the HEBECO study
Source: BMJ Open. 2022 Jun 21;12(6):e054029. doi: 10.1136/bmjopen-2021-054029 (PMC9226469; doi:10.1136/bmjopen-2021-054029)
Supplement: Supplementary data [file bmjopen-2021-054029supp001.pdf]

Supplementary Materials 1-5

Manuscript Title: Correlates of and changes in aerobic physical activity and strength training before and after the onset of COVID-19 pandemic in the UK – findings from the HEBECO study.

Article pre-registration: https://osf.io/spdtb

Contents

Supplementary Materials 1: Changes to the pre-registered protocol.. 1
Supplementary Materials 2: detailed description of study measures.. 2
Supplementary materials 3: Univariable analyses.. 4
Supplementary Materials 4: Unweighted analyses 7
Supplementary Materials 5: Sensitivity Analyses for RQ3 (different cut-off values for MVPA change). 12

Supplementary Materials 1: Changes to the pre-registered protocol

The analysis protocol pre-registered on OSF prior to data inspection outlined analyses for research questions 3 and 4 involving unstratified multinomial analyses with change in MVPA and MSA as dependent variables. Specifically, the pre-registered protocol specified that we will use multinomial regression models to assess the correlates of a three-level dependent variables on change in (i) MVPA (increase by at least 20min/week, maintenance with change between 0 to 19min, and a decrease in MVPA by at least 20min/week) and in (ii) MSA (increase by at least 1 day/week, maintenance with change=0 days/week, and a decrease with change by at least 1 day/week). All the analyses were performed as per the original plan and can be accessed here: https://osf.io/2ujxq/.

Upon inspecting the data and the results a decision was made that this approach is not satisfactory as the group of maintainers of MVPA and MSA category included both inactive and active participants. This has motivated us to conduct and report the present analyses that stratified the sample based on pre-Covid-19 activity levels to identify meaningful changes.

Contact: Aleksandra Herbec a.herbec@ucl.ac.uk

Supplementary Materials 2: detailed description of study measures

Below are the details of the all the explanatory measures assessed at baseline that are relevant for the study. For the full wording of the baseline survey see: https://osf.io/bja7g/

Socio-demographic, health and wellbeing characteristics

Gender (female gender/other; prefer not to say (PNS) were treated as missing) was assessed by asking participants: "Which of the following best describes how you think of yourself?".

Ethnicity (white/non-white; PNS treated as missing) was assessed by asking participants: "What is your ethnic group?".

Education (high school education or higher; yes/no) was assessed by asking participants: "What is the highest level of education that you have completed?"

Employment (employed or self-employed; yes/no) and **furloughed/laid off** (yes/no) were assessed by asking participants: "What is your current MAIN occupation (during COVID-19)?".

Income (low-middle <50 000 GBP vs high ≥50 000 GBP vs PNS) was assessed by asking participants: "What was your household annual income BEFORE COVID-19? (do not include income of friends)".

Age was assessed as a continuous variable. For the analyses we used age in decades and entered it as a continuous variable. Where the continuous variable violated the linearity assumptions of the logistic regression models (as did its logarithmic and square root transformation), a 3-level categorical variable was created (≤34, 35-64, 65+).

Health behaviours and conditions

- **Body mass index, BMI** (normal and underweight ≤24.99/overweight 25-29.99/ obese ≥30/ unknown (included answers 'don't know' and PNS to height or weight variables)). BMI was calculated by dividing participants weight in kilogram by their squared height in meters and categorised into: normal weight and underweight. BMI is associated with both MVPA and MSA levels (Churilla et al., 2018).

- **Self-reports of any condition that limited their ability to engage in physical activity** (yes/no; PNS treated as missing). Participants were asked: "Do you now suffer from any condition that limits you from engaging in physical activity, including walking or doing housework?"

- **Self-perceived risk of Covid-19 to one's health** (minor or no risk/major, significant, moderate risk or 'don't know') was assessed with the question: "What risk does COVID-19 pose to your health?"

- **Smoking status** (current smokers vs not) was assessed using a single item: "Which statement about tobacco use and cigarette smoking best describes you?". Those responding 1 ("I smoke cigarettes (including hand-rolled) every day") and 2 ("I smoke cigarettes (including hand-rolled), but not every day") were classified as current smokers. Smoking is associated with physical activity levels (Kaczynski, Manske, Mannell, & Grewal, 2008).

Contact: Aleksandra Herbec a.herbec@ucl.ac.uk

- **Weekly alcohol consumption** since the Covid-19 pandemic has started was assessed by asking participants how many times they had a drink containing alcohol, with the categorical answers converted to weekly frequencies from 0 to 6.5 times per week (0=I have not had any alcohol after COVID-19 started, 0.25=monthly or less, 0.75=2 to 4 times a month, 2.5=2 to 3 times a week, 4.5=4 to 5 times a week, 6.5=6 or more times a week). In models where the continuous alcohol frequency variable violated the linearity assumptions of the logistic regression models (as did its logarithmic and square root transformation), weekly alcohol frequency was categorised into three levels (none/weekly or less often/more frequently than weekly). Alcohol drinking is associated with physical activity levels (Leasure, Neighbors, Henderson, & Young, 2015).

- **Deterioration in psychological wellbeing** (yes/no). Deterioration in psychological wellbeing was assessed on a five-point Likert scale (1=poor, 3=average, 5=excellent) by asking participants: "How would you rate the below aspects of your life? – Psychological wellbeing". The question was asked about the period before Covid-19 and since Covid-19. A change score was calculated by deducting the score before from since score. As the continuous variable violated the linearity assumptions of the logistic regression models (as did its logarithmic and square root transformation), participants were categorised into either deteriorated psychological wellbeing (change score < 0) versus maintained or improved psychological wellbeing (change score ≥ 0).

- **Isolation status** (total isolation/other) was assessed by asking which type of Covid-19 induced isolation participants were experiencing. Answers were dichotomised into indications of total isolation/quarantine ("not leaving the house for any reasons, not even to buy groceries or medications or to exercise") versus any other (some, general, or no isolation). Participants' perceived risk of Covid-19 to their health was dichotomised into 'no or minor risk perception' versus any other indication (moderate risk, significant, major or not known). It was hypothesised that perceived risk could affect physical activity, especially MVPA that may happen outdoors.

- **Access to space to exercise** was assessed using a single question on participants' access to spaces that allowed multiple answers, including for: „a garden or balcony big enough to exercise comfortably" and to "a public park/green space that is within a walking distance and open during Covid-19". The answers were dummy coded into separate binary variables (yes/no).

- **Living with children age 15 and younger** (yes/no) and **living with vulnerable persons** (any of: persons over the age of 70, persons in poor health versus, or persons who they believed may be vulnerable to Covid-19 for any reason) was assessed by asking participants: "Do you live with any of these persons below? (select all that apply)." It was hypothesised that living with these groups could limit some of the opportunities to exercise.

Contact: Aleksandra Herbec a.herbec@ucl.ac.uk

Supplementary materials 3: Univariable analyses.

Supplementary Table S3.1. (univariable) Research question 2 -Predictors of meeting both, MVPA only and MSA only in comparison to meeting WHO guidelines for neither MVPA nor MSA (reference, n=1178). Results from unadjusted models on weighted data with BH FDR correction of p-values (significant in bold).

	Both (n=388)				MVPA only (n=570)				MSA only (n=306)			
	OR	95% CI		*p*	OR	95% CI		*p*	OR	95% CI		*p*
Female	1.01	0.80	1.27	0.928	**0.62**	**0.51**	**0.76**	**<0.001**	0.93	0.72	1.19	0.546
Age ≤34[a]												
Age: 35-64	**0.57**	**0.43**	**0.75**	**<0.001**	1.08	0.82	1.42	0.604	**0.41**	**0.30**	**0.56**	**<0.001**
Age: 65+	**0.41**	**0.28**	**0.60**	**<0.001**	1.02	0.73	1.43	0.893	**0.58**	**0.40**	**0.84**	**0.004**
White ethnicity	0.77	0.52	1.12	0.166	1.53	1.02	2.30	0.040	**0.43**	**0.30**	**0.62**	**<0.001**
Household income ≥50.000												
Income: <50 000 GBP	**0.44**	**0.33**	**0.59**	**<0.001**	**0.66**	**0.50**	**0.85**	**0.002**	0.80	0.57	1.12	0.194
Income: Prefer not to say	**0.41**	**0.25**	**0.66**	**<0.001**	**0.50**	**0.32**	**0.77**	**0.002**	0.79	0.47	1.34	0.385
Education: Highschool or higher	**2.57**	**1.96**	**3.38**	**<0.001**	**1.34**	**1.09**	**1.65**	**0.006**	**2.41**	**1.79**	**3.24**	**<0.001**
Employed	1.14	0.90	1.43	0.272	1.20	0.98	1.46	0.077	1.05	0.81	1.35	0.714
Laid-off/Furloughed	0.95	0.67	1.35	0.784	1.01	0.75	1.36	0.972	1.04	0.72	1.51	0.839
Condition limiting PA	**0.24**	**0.16**	**0.37**	**<0.001**	**0.27**	**0.19**	**0.37**	**<0.001**	**0.64**	**0.46**	**0.89**	**0.009**
BMI ≤24												
BMI: 25-29.99	**0.62**	**0.48**	**0.81**	**<0.001**	0.86	0.68	1.08	0.191	**0.60**	**0.45**	**0.79**	**<0.001**
BMI: Obese: 30+	**0.31**	**0.22**	**0.43**	**<0.001**	**0.62**	**0.48**	**0.81**	**<0.001**	**0.27**	**0.19**	**0.40**	**<0.001**
Total isolation	**0.30**	**0.17**	**0.52**	**<0.001**	**0.17**	**0.10**	**0.31**	**<0.001**	0.67	0.43	1.05	0.079
Minor/no Covid-19 risk percept	**1.70**	**1.34**	**2.16**	**<0.001**	**1.39**	**1.12**	**1.72**	**0.002**	**1.37**	**1.05**	**1.79**	**0.019**
Living with children	0.99	0.73	1.33	0.925	0.81	0.61	1.06	0.121	0.70	0.49	1.01	0.056
Living with vulnerable	1.09	0.80	1.48	0.583	0.80	0.59	1.06	0.122	0.87	0.61	1.25	0.450
Access garden/balcony to exercise comfortably in	1.18	0.91	1.54	0.212	1.01	0.81	1.27	0.918	0.82	0.63	1.08	0.157
Access green space within walking distance	**1.70**	**1.33**	**2.16**	**<0.001**	**1.59**	**1.29**	**1.95**	**<0.001**	1.29	1.00	1.67	0.049
Smoker	**0.48**	**0.35**	**0.64**	**<0.001**	**0.72**	**0.57**	**0.91**	**0.006**	**0.64**	**0.47**	**0.87**	**0.004**
Alcohol drinking frequency, never (ref)												
Weekly or less	1.32	0.94	1.85	0.106	**1.50**	**1.12**	**2.02**	**0.006**	1.48	1.04	2.11	0.032
More than weekly	**1.47**	**1.09**	**1.98**	**0.012**	**1.50**	**1.15**	**1.95**	**0.003**	1.21	0.87	1.68	0.249
Deteriorated psychological wellbeing	**0.62**	**0.50**	**0.79**	**<0.001**	**0.71**	**0.58**	**0.87**	**0.001**	0.74	0.58	0.95	0.019
Meeting WHO PA rec (meeting neither=ref)												
Meeting both before	**17.58**	**12.53**	**24.65**	**<0.001**	**2.30**	**1.60**	**3.31**	**<0.001**	**4.03**	**2.72**	**5.99**	**<0.001**
Meeting only MVPA before	**4.53**	**3.13**	**6.55**	**<0.001**	**8.30**	**6.47**	**10.65**	**<0.001**	1.67	1.08	2.58	0.021
Meeting only MSA before	**7.72**	**5.27**	**11.32**	**<0.001**	0.86	0.52	1.42	0.549	**10.31**	**7.37**	**14.43**	**<0.001**

Contact: Aleksandra Herbec a.herbec@ucl.ac.uk

Supplementary Table S3.2. (univariable) Research question 3: Independent associations of change in MVPA. Segmented analyses of active sample (pre-Covid-19 MVPA activity ≥30min; predicting decrease by ≥20min) and less active/inactive sample (pre-Covid-19 MVPA activity <30min; predicting increase by ≥20min). Findings from unadjusted logistic regression models on weighted data and using BH FDR adjustment (significant in bold).

	Sample active (≥30min/week) pre-Covid-19 (weighted n=1857)				Sample inactive (<30min/week) pre-Covid-19 (weighted n=585)			
	Decrease (n=868) vs not				Increase (n=174) vs not			
	OR	95% CI		*p*	OR	95% CI		*p*
Female	**1.38**	**1.15**	**1.65**	**0.001**	1.13	0.79	1.62	0.496
Age (in decades)	0.94	0.88	0.99	0.024	**0.67**	**0.59**	**0.75**	**<0.001**
White ethnicity	**0.57**	**0.42**	**0.77**	**<0.001**	0.46	0.24	0.88	0.019
Household income ≥50.000								
Income: <50 000 GBP	**1.36**	**1.08**	**1.71**	**0.010**	**0.45**	**0.27**	**0.77**	**0.003**
Income: Prefer not to say	**1.59**	**1.09**	**2.33**	**0.017**	0.53	0.25	1.14	0.103
Education: Highschool or higher	**0.72**	**0.59**	**0.88**	**0.001**	**2.67**	**1.81**	**3.93**	**<0.001**
Employed	0.82	0.69	0.99	0.036	0.80	0.55	1.16	0.235
Laid-off/Furloughed	1.22	0.92	1.62	0.165	**2.16**	**1.36**	**3.43**	**0.001**
Condition limiting PA	**2.28**	**1.71**	**3.04**	**<0.001**	**0.27**	**0.16**	**0.43**	**<0.001**
BMI ≤24								
BMI: 25-29.99	1.01	0.82	1.23	0.961	1.04	0.69	1.55	0.863
BMI: Obese: 30+	0.99	0.78	1.27	0.945	0.56	0.34	0.90	0.016
Total isolation	**4.95**	**3.11**	**7.88**	**<0.001**	**0.22**	**0.10**	**0.49**	**<0.001**
Minor/no Covid-19 risk percept	0.94	0.77	1.14	0.503	**2.17**	**1.50**	**3.14**	**<0.001**
Living with children	0.89	0.70	1.13	0.319	1.37	0.82	2.30	0.225
Living with vulnerable	**0.72**	**0.56**	**0.93**	**0.012**	1.14	0.70	1.85	0.599
Access garden/balcony to exercise comfortably in	0.82	0.67	1.00	0.053	**0.61**	**0.42**	**0.89**	**0.011**
Access green space within walking distance	0.81	0.67	0.98	0.026	**1.66**	**1.16**	**2.37**	**0.006**
Smoker	0.94	0.75	1.17	0.576	1.15	0.79	1.68	0.454
Alcohol drinking frequency, never (ref)								
Weekly or less	**0.68**	**0.52**	**0.89**	**0.005**	1.19	0.73	1.96	0.483
More than weekly	**0.60**	**0.47**	**0.76**	**<0.001**	1.22	0.78	1.90	0.387
Deteriorated psychological wellbeing	**1.42**	**1.18**	**1.71**	**<0.001**	1.03	0.73	1.47	0.854

Adjusted for the time of enrolment and time when Covid-19 started to affect individuals in any way.

Contact: Aleksandra Herbec a.herbec@ucl.ac.uk

Supplementary Table S3.3 Research question 4: Independent associations of change in MSA. Segmented analyses of active sample (pre-Covid-19 MSA activity ≥1 day/week; predicting decrease by ≥1 day/week) and less inactive sample (pre-Covid-19 MSA activity 0 days/week; predicting increase by ≥1 day/week). Findings from unadjusted logistic regression models on weighted data and using BH FDR adjustment (significant in bold).

	Sample active (≥1 day/week) pre-Covid-19 (weighted n=985)				Sample inactive (0 days/week) pre-Covid-19 (weighted n=1456)			
	Decrease (n=444) vs not				Increase (n=239) vs not			
	OR	95% CI		*p*	OR	95% CI		*p*
Female	0.87	0.67	1.12	0.274	0.85	0.64	1.12	0.249
Age (in decades)	1.02	0.94	1.10	0.625	-	-	-	-
Age ≤34[a]								
Age: 35-64	-	-	-	-	**0.20**	**0.14**	**0.28**	**<0.001**
Age: 65+	-	-	-	-	**0.27**	**0.18**	**0.40**	**<0.001**
White ethnicity	0.80	0.57	1.13	0.210	**0.36**	**0.22**	**0.58**	**<0.001**
Household income ≥50.000								
Income: <50 000 GBP	1.01	0.75	1.36	0.953	**0.61**	**0.42**	**0.87**	**0.007**
Income: Prefer not to say	0.80	0.48	1.31	0.368	0.64	0.36	1.17	0.147
Education: Highschool or higher	0.74	0.55	1.00	0.051	**1.86**	**1.37**	**2.52**	**<0.001**
Employed	**1.60**	**1.24**	**2.06**	**<0.001**	1.01	0.76	1.33	0.951
Laid-off/Furloughed	0.99	0.67	1.45	0.954	1.33	0.91	1.96	0.141
Condition limiting PA	1.35	0.92	1.97	0.120	**0.43**	**0.27**	**0.67**	**<0.001**
BMI ≤24								
BMI: 25-29.99	**1.88**	**1.42**	**2.50**	**<0.001**	0.68	0.50	0.93	0.016
BMI: Obese: 30+	**3.20**	**2.18**	**4.72**	**<0.001**	**0.43**	**0.29**	**0.63**	**<0.001**
Total isolation	**2.05**	**1.22**	**3.45**	**0.007**	0.62	0.35	1.10	0.102
Minor/no Covid-19 risk percept	1.07	0.83	1.38	0.596	**1.61**	**1.20**	**2.15**	**0.001**
Living with children	0.83	0.60	1.15	0.266	1.44	0.95	2.18	0.086
Living with vulnerable	1.13	0.79	1.63	0.498	1.01	0.69	1.47	0.971
Access garden/balcony to exercise comfortably in	0.90	0.67	1.19	0.453	0.74	0.55	0.99	0.043
Access green space within walking distance	0.94	0.72	1.22	0.621	1.12	0.85	1.49	0.420
Smoker	0.65	0.46	0.90	0.010	1.00	0.74	1.36	0.995
Alcohol drinking frequency, never (ref)								
Weekly or less	0.88	0.61	1.27	0.508	1.50	1.00	2.27	0.051
More than weekly	0.93	0.67	1.30	0.677	1.40	0.97	2.03	0.072
Deteriorated psychological wellbeing	1.37	1.06	1.77	0.014	0.72	0.55	0.95	0.021

Adjusted for the time of enrolment and time when Covid-19 started to affect individuals in any way.

[a]Age was categorised in models where the continuous predictor violated the linearity assumption.

Contact: Aleksandra Herbec a.herbec@ucl.ac.uk

Supplementary Materials 4: Unweighted analyses

Supplementary Table S4.1. (unweighted data) Sample characteristics % (N).

	Included sample (2657)	Excluded sample (336)	*p*
Female	68.9 (1831)	68.1 (224)	0.760
Age; M (SD)	48.4 (15.2)	43.7 (16.7)	<0.001
White ethnicity	94.4 (2507)	91.1 (297)	0.020
Household income ≥50.000	90.9 (993)	9.1 (100)	<0.001
Income: <50 000 GBP	54.5 (1447)	55.5 (186)	
Income: Prefer not to say	8.2 (217)	14.6 (49)	
Education: Highschool or higher	86.8 (2307)	85.7 (288)	0.571
Employed	56.1 (1490)	49.1 (164)	0.016
Laid-off/Furloughed	9.8 (261)	12.3 (41)	0.161
Condition limiting PA	13.2 (351)	19.6 (54)	0.003
BMI ≤24	49.1 (1305)	23.8 (62)	<0.001
BMI: 25-29.99	31.3 (832)	15.8 (41)	
BMI: Obese: 30+	19.6 (520)	9.2 (24)	
Total isolation	7.2 (190)	7.2 (21)	0.955
Minor/no Covid-19 risk percept	37.5 (996)	42.4 (123)	0.101
Living with children	19.6 (520)	19.0 (64)	0.820
Living with vulnerable	14.8 (394)	16.1 (54)	0.547
Access garden/balcony	71.9 (1911)	63.1 (212)	0.001
Access green space	63.5 (1687)	57.1 (192)	0.023
Time affected up till mid March	49.5 (1316)	55.1 (185)	0.056
Enrolled from 1st June (ref)	5.2 (139)	4.8 (16)	0.479
Enrolled up until 15th May	48.9 (1298)	45.8 (154)	
Enrolled second half of May	45.9 (1220)	49.4 (166)	
Smoker	17.7 (470)	25.6 (86)	<0.001
Alcohol drinking frequency, never (ref)	17.0 (453)	19.6 (66)	0.092
Weekly or less	27.5 (732)	31.3 (105)	
More than weekly	55.4 (1472)	49.1 (165)	
Deteriorated psychological wellbeing	55.1 (1465)	50.2 (117)	0.148
Meeting WHO PA rec (meeting both=ref)	17.9 (475)	14.9 (26)	0.332
Meeting only MVPA before	22.8 (605)	23.0 (40)	
Meeting only MSA before	14.9 (395)	11.5 (20)	
Meeting neither before	44.5 (1182)	50.6 (88)	

Contact: Aleksandra Herbec a.herbec@ucl.ac.uk

Supplementary Table S4.2. (unweighted data) Aerobic physical activity (MVPA) and strength training (MSA) before to after the Covid-19 pandemic has started to affect individuals in the UK (up until 14th June 2020).

	Before Covid-19[1]	Since Covid-19[1]
Inactive (0 sessions MVPA and 0 days MSA) % (N)	14.1 (375)	16.0 (425)
MVPA 0 sessions % (N)	15.9 (422)	18.5 (491)
MVPA		
MVPA min/week among active; Median (IQR)	135.0 (180.0)	150.0 (240.0)
Mean (SD)	215.3 (363.4)	227.0 (341.7)
Mean (SD) replacing extremes with 840 min/week	191.8 (186.9)	206.9 (193.7)
MVPA min/week among entire sample; Median (IQR)	120.0 (185.0)	120.0 (215.0)
Mean (SD)	184.9 (345.0)	190.7 (324.1)
Mean (SD) replacing extremes with 840 min/week	164.7 (185.6)	173.8 (193.1)
MSA Median (IQR)	0 (2)	0 (2)
MSA % (N)		
0 das/week	53.0 (1408)	54.0 (1435)
1 day/week	14.3 (379)	11.7 (311)
2 days/week	14.6 (387)	10.5 (280)
3 days/week	10.4 (277)	9.6 (254)
4+ days/week	7.8 (206)	14.2 (377)
Meet guidelines % (N)		
Neither MVPA nor MSA	44.5 (1182)	42.0 (1117)
Meet MVPA (≥150min/week) only	22.8 (605)	23.7 (629)
Meet MSA (≥2 days/week) only	14.9 (395)	14.7 (390)
Meets both MVPA and MSA	17.9 (475)	19.6 (521)
Change in form of exercise % (N)		
None of the same		13.2 (350)
Some of the same		29.1 (774)
About half the same		8.8 (233)
Mostly the same		23.4 (622)
Exactly the same		10.0 (267)

Contact: Aleksandra Herbec a.herbec@ucl.ac.uk

Supplementary Table S4.3. (unweighted data) Research question 2 – Factors associated with meeting both, MVPA only and MSA only in comparison to meeting WHO guidelines for neither MVPA nor MSA (reference, n=1117) after the Covid-19 pandemic has started to affect individuals in the UK (up until June 2020[1]). Results from fully adjusted models on unweighted data with BH FDR correction of p-values (significant in bold).

	Both (n=521)				MVPA only (n=629)				MSA only (n=390)			
Characteristics and associations	aOR	95% CI		*p*	aOR	95% CI		*p*	aOR	95% CI		*p*
Female	1.17	0.89	1.52	0.256	0.93	0.73	1.18	0.546	1.16	0.87	1.53	0.308
Age ≤34[a]												
Age: 35-64	0.92	0.67	1.25	0.589	**1.49**	**1.09**	**2.05**	**0.013**	**0.59**	**0.43**	**0.82**	**0.001**
Age: 65+	0.64	0.40	1.03	0.067	**1.80**	**1.17**	**2.75**	**0.007**	0.90	0.57	1.44	0.664
White ethnicity	1.36	0.81	2.27	0.245	1.79	1.01	3.15	0.044	0.67	0.42	1.08	0.099
Household income ≥50.000												
Income: <50 000 GBP	**0.58**	**0.44**	**0.76**	**<0.001**	**0.70**	**0.55**	**0.90**	**0.006**	1.00	0.75	1.33	0.976
Income: Prefer not to say	**0.55**	**0.34**	**0.89**	**0.014**	0.76	0.49	1.17	0.214	0.90	0.54	1.48	0.667
Education: Highschool or higher	1.38	0.92	2.09	0.121	1.20	0.86	1.67	0.293	**1.86**	**1.19**	**2.90**	**0.007**
Employed	0.78	0.58	1.05	0.102	1.11	0.85	1.47	0.442	1.22	0.89	1.68	0.212
Laid-off/Furloughed	1.00	0.64	1.55	0.997	1.07	0.70	1.62	0.759	1.33	0.84	2.13	0.227
Condition limiting PA	**0.42**	**0.27**	**0.65**	**<0.001**	**0.36**	**0.24**	**0.54**	**<0.001**	0.78	0.53	1.16	0.219
BMI ≤24												
BMI: 25-29.99	**0.61**	**0.46**	**0.80**	**<0.001**	0.80	0.62	1.02	0.076	**0.66**	**0.49**	**0.88**	**0.005**
BMI: Obese: 30+	**0.35**	**0.24**	**0.51**	**<0.001**	**0.69**	**0.51**	**0.93**	**0.014**	**0.43**	**0.30**	**0.62**	**<0.001**
Total isolation	**0.39**	**0.22**	**0.69**	**0.001**	**0.22**	**0.12**	**0.41**	**<0.001**	0.84	0.52	1.38	0.498
Minor/no Covid-19 risk percept	0.82	0.64	1.07	0.147	0.92	0.72	1.17	0.476	0.90	0.68	1.20	0.477
Living with children	0.79	0.58	1.07	0.130	**0.56**	**0.42**	**0.76**	**<0.001**	0.89	0.64	1.24	0.497
Living with vulnerable	1.14	0.81	1.60	0.457	1.07	0.79	1.46	0.662	0.94	0.65	1.35	0.721
Access garden/balcony to exercise comfortably in	1.09	0.82	1.44	0.562	1.00	0.78	1.29	0.994	0.98	0.74	1.31	0.901
Access green space within walking distance	1.17	0.90	1.51	0.243	1.23	0.97	1.56	0.081	1.16	0.89	1.53	0.278
Smoker	**0.56**	**0.40**	**0.79**	**0.001**	**0.69**	**0.52**	**0.93**	**0.016**	**0.65**	**0.46**	**0.92**	**0.016**
Alcohol drinking frequency, never (ref)												
Weekly or less	1.24	0.84	1.82	0.279	1.16	0.82	1.63	0.405	1.07	0.72	1.58	0.745
More than weekly	1.25	0.88	1.78	0.221	1.14	0.83	1.56	0.412	1.02	0.71	1.47	0.923
Deteriorated psychological wellbeing	**0.61**	**0.48**	**0.78**	**<0.001**	0.78	0.63	0.98	0.029	**0.65**	**0.51**	**0.84**	**0.001**
Meeting WHO PA rec (meeting neither=ref)												
Meeting both before	**14.58**	**10.48**	**20.30**	**<0.001**	**2.47**	**1.74**	**3.52**	**<0.001**	**3.53**	**2.44**	**5.10**	**<0.001**
Meeting only MVPA before	**3.31**	**2.35**	**4.66**	**<0.001**	**5.86**	**4.54**	**7.55**	**<0.001**	0.84	0.54	1.31	0.445
Meeting only MSA before	**4.98**	**3.49**	**7.11**	**<0.001**	0.79	0.51	1.24	0.303	**6.59**	**4.80**	**9.06**	**<0.001**

Models adjusted for the time of enrolment and time when Covid-19 started to affect individuals in any way.

[1] Covering the period of the strictest first lockdown in the UK

Contact: Aleksandra Herbec a.herbec@ucl.ac.uk

Supplementary Table S4.4. Research question 3: Independent associations of change in MVPA from before to after the Covid-19 pandemic has started to affect individuals in the UK (up until 14th June 2020[2]). Segmented analyses of active sample (pre-Covid-19 MVPA activity ≥30min; predicting decrease by ≥20min) and less active/inactive sample (pre-Covid-19 MVPA activity <30min; predicting increase by ≥20min). Findings from fully adjusted logistic regression models on unweighted data and using BH FDR adjustment (significant in bold).

Characteristics and associations	Sample active (≥30min/week) pre-Covid-19 (unweighted n=2132)				Sample inactive (<30min/week) pre-Covid-19 (unweighted n=525)			
	Decrease (n=905) vs not				Increase (n=195) vs not			
	aOR	95% CI		*p*	aOR	95% CI		*p*
Female	1.05	0.86	1.27	0.660	1.12	0.73	1.74	0.605
Age (in decades)	**0.91**	**0.85**	**0.98**	**0.011**	**0.80**	**0.68**	**0.94**	**0.007**
White ethnicity	**0.52**	**0.35**	**0.76**	**0.001**	0.80	0.30	2.15	0.656
Household income ≥50.000								
Income: <50 000 GBP	**1.41**	**1.15**	**1.73**	**0.001**	0.95	0.59	1.53	0.829
Income: Prefer not to say	1.31	0.92	1.87	0.130	0.72	0.32	1.65	0.441
Education: Highschool or higher	0.73	0.54	0.98	0.036	1.87	1.07	3.26	0.028
Employed	0.96	0.77	1.18	0.682	0.70	0.43	1.13	0.140
Laid-off/Furloughed	0.95	0.68	1.34	0.782	1.51	0.77	2.93	0.227
Condition limiting PA	**1.87**	**1.37**	**2.55**	**<0.001**	**0.47**	**0.27**	**0.83**	**0.010**
BMI ≤24								
BMI: 25-29.99	1.08	0.88	1.33	0.456	1.11	0.69	1.79	0.665
BMI: Obese: 30+	0.97	0.75	1.26	0.833	0.71	0.42	1.19	0.195
Total isolation	**2.91**	**1.89**	**4.46**	**<0.001**	**0.22**	**0.09**	**0.54**	**0.001**
Minor/no Covid-19 risk percept	1.21	0.99	1.47	0.067	1.19	0.76	1.87	0.441
Living with children	0.80	0.64	1.01	0.061	0.86	0.52	1.44	0.570
Living with vulnerable	0.92	0.71	1.20	0.551	0.83	0.49	1.43	0.511
Access garden/balcony to exercise comfortably in	**0.74**	**0.60**	**0.91**	**0.005**	0.96	0.61	1.49	0.847
Access green space within walking distance	0.95	0.78	1.15	0.589	1.45	0.96	2.19	0.077
Smoker	0.88	0.68	1.13	0.311	1.70	1.05	2.74	0.030
Alcohol drinking frequency, never (ref)								
Weekly or less	1.08	0.81	1.44	0.605	0.96	0.54	1.71	0.890
More than weekly	0.89	0.68	1.16	0.378	1.24	0.75	2.06	0.399
Deteriorated psychological wellbeing	**1.40**	**1.16**	**1.68**	**<0.001**	1.12	0.74	1.67	0.597

Models adjusted for the time of enrolment and time when Covid-19 started to affect individuals in any way.

[2] Covering the period of the strictest first lockdown in the UK

Contact: Aleksandra Herbec a.herbec@ucl.ac.uk

Supplementary Table S4.5. (unweighted data). Research question 4: Independent associations of change in MSA from before to after the Covid-19 pandemic has started to affect individuals in the UK (up until 14th June 2020[3]). Segmented analyses of active sample (pre-Covid-19 MSA activity ≥1 day/week; predicting decrease by ≥1 day/week) and less inactive sample (pre-Covid-19 MSA activity 0 days/week; predicting increase by ≥1 day/week). Findings from fully adjusted logistic regression models on unweighted data and using BH FDR adjustment (significant in bold).

	Sample active (≥1 day/week) pre-Covid-19 (unweighted n=1249)				Sample inactive (0 days/week) pre-Covid-19 (unweighted n=1408)			
	Decrease (n=534) vs not				Increase (n=284) vs not			
Characteristics and associations	aOR	95% CI		*p*	aOR	95% CI		*p*
Female	0.83	0.64	1.08	0.160	1.216	0.899	1.647	0.205
Age (in decades)	1.03	0.94	1.12	0.579	-	-	-	-
Age ≤34[a]								
Age: 35-64	-	-	-	-	**0.353**	**0.248**	**0.502**	**<0.001**
Age: 65+	-	-	-	-	**0.305**	**0.178**	**0.524**	**<0.001**
White ethnicity	0.89	0.57	1.39	0.611	0.553	0.298	1.024	0.060
Household income ≥50.000								
Income: <50 000 GBP	1.25	0.96	1.64	0.096	**0.678**	**0.496**	**0.928**	**0.015**
Income: Prefer not to say	1.39	0.88	2.18	0.155	0.480	0.258	0.893	0.020
Education: Highschool or higher	0.96	0.62	1.49	0.863	1.310	0.837	2.051	0.237
Employed	**1.49**	**1.12**	**1.97**	**0.005**	0.900	0.634	1.278	0.557
Laid-off/Furloughed	1.18	0.75	1.85	0.467	1.104	0.676	1.803	0.694
Condition limiting PA	1.08	0.72	1.62	0.708	0.664	0.409	1.078	0.097
BMI ≤24								
BMI: 25-29.99	**1.63**	**1.24**	**2.13**	**<0.001**	0.846	0.613	1.168	0.310
BMI: Obese: 30+	**2.50**	**1.73**	**3.60**	**<0.001**	0.707	0.484	1.031	0.072
Total isolation	**2.03**	**1.18**	**3.50**	**0.011**	0.861	0.481	1.543	0.615
Minor/no Covid-19 risk percept	1.18	0.91	1.52	0.216	1.045	0.769	1.421	0.777
Living with children	1.04	0.77	1.41	0.784	0.708	0.503	0.997	0.048
Living with vulnerable	1.11	0.78	1.59	0.553	0.990	0.677	1.446	0.957
Access garden/balcony to exercise comfortably in	0.86	0.65	1.13	0.275	0.765	0.562	1.040	0.088
Access green space within walking distance	0.95	0.74	1.23	0.713	1.102	0.816	1.489	0.527
Smoker	**0.61**	**0.43**	**0.87**	**0.007**	1.096	0.772	1.557	0.608
Alcohol drinking frequency, never (ref)								
Weekly or less	1.01	0.69	1.49	0.943	1.192	0.776	1.833	0.422
More than weekly	0.99	0.69	1.42	0.951	1.124	0.755	1.672	0.565
Deteriorated psychological wellbeing	**1.38**	**1.09**	**1.76**	**0.008**	**0.643**	**0.488**	**0.848**	**0.002**

Models adjusted for the time of enrolment and time when Covid-19 started to affect individuals in any way.
[a]Age was categorised in models where the continuous predictor violated the linearity assumption.

[3] Covering the period of the strictest first lockdown in the UK

Contact: Aleksandra Herbec a.herbec@ucl.ac.uk

Supplementary Materials 5: Sensitivity Analyses for RQ3 (different cut-off values for MVPA change).

Supplementary Table S5.1. Predicting MVPA decrease of at least 15 min in the active sample from before to after the Covid-19 pandemic has started to affect individuals in the UK (up until 14th June 2020). (fully adjusted model, weighted data).

Characteristics and associations	**aOR**	**95% CI**		***p***
Female	1.232	1.010	1.503	.039
Age (in decades)	**.914**	**.849**	**.983**	**.015**
White ethnicity	**.649**	**.464**	**.909**	**.012**
Household income ≥50.000	1.0			
Income: <50 000 GBP	1.272	.986	1.641	.064
Income: Prefer not to say	1.182	.783	1.783	.427
Education: Highschool or higher	**.754**	**.602**	**.944**	**.014**
Employed	.841	.677	1.045	.118
Laid-off/Furloughed	1.025	.738	1.423	.884
Condition limiting PA	**1.732**	**1.260**	**2.381**	**.001**
BMI ≤24	1.0			
BMI: 25-29.99	1.025	.822	1.280	.824
BMI: Obese: 30+	.875	.667	1.147	.334
Total isolation	**3.682**	**2.252**	**6.020**	**.000**
Minor/no Covid-19 risk percept	.984	.791	1.224	.882
Living with children	.858	.661	1.114	.251
Living with vulnerable	.801	.607	1.058	.118
Access garden/balcony to exercise comfortably in	.773	.618	.968	.025
Access green space within walking distance	.813	.662	.998	.048
Smoker	1.106	.861	1.420	.432
Alcohol drinking frequency, never (ref)	1.0			
Weekly or less	.790	.596	1.049	.103
More than weekly	.772	.594	1.003	.053
Deteriorated psychological wellbeing	**1.422**	**1.170**	**1.728**	**.000**

Model was adjusted for all the variable sin the table as well as the time of enrolment and time when Covid-19 started to affect individuals in any way. Significant results are in bold.

Contact: Aleksandra Herbec a.herbec@ucl.ac.uk

Supplementary Table S5.2. Predicting MVPA increase of at least 15 min in the active sample from before to after the Covid-19 pandemic has started to affect individuals in the UK (up until 14th June 2020; fully adjusted model, weighted data).

Characteristics and associations	**aOR**	**95% CI**		**p**
Female	1.513	1.003	2.282	.048
Age (in decades)	**.750**	**.642**	**.876**	**.000**
White ethnicity	.564	.249	1.276	.169
Household income ≥50.000	1.0			
Income: <50 000 GBP	.633	.339	1.182	.152
Income: Prefer not to say	.663	.271	1.620	.367
Education: Highschool or higher	1.700	1.078	2.683	.023
Employed	.846	.526	1.362	.492
Laid-off/Furloughed	1.380	.762	2.498	.288
Condition limiting PA	**.445**	**.249**	**.795**	**.006**
BMI ≤24	1.0			
BMI: 25-29.99	1.640	1.014	2.652	.044
BMI: Obese: 30+	.778	.451	1.343	.368
Total isolation	.395	.172	.907	.029
Minor/no Covid-19 risk percept	1.349	.857	2.122	.196
Living with children	1.600	.917	2.793	.098
Living with vulnerable	.872	.505	1.508	.624
Access garden/balcony to exercise comfortably in	.730	.469	1.136	.163
Access green space within walking distance	.986	.656	1.481	.944
Smoker	1.491	.963	2.309	.073
Alcohol drinking frequency, never (ref)	1.0			
Weekly or less	.719	.407	1.272	.257
More than weekly	.941	.567	1.563	.814
Deteriorated psychological wellbeing	1.275	.850	1.913	.241

Model was adjusted for all the variable sin the table as well as the time of enrolment and time when Covid-19 started to affect individuals in any way. Significant results are in bold.

Contact: Aleksandra Herbec a.herbec@ucl.ac.uk

Supplementary Table S5.3. Predicting MVPA decrease of at least 30 min in the active sample (fully adjusted model, weighted data).

Characteristics and associations	**aOR**	**95% CI**		**p**
Female	1.207	.989	1.474	.064
Age (in decades)	**.904**	**.841**	**.973**	**.007**
White ethnicity	**.618**	**.441**	**.865**	**.005**
Household income ≥50.000	1.0			
Income: <50 000 GBP	1.356	1.049	1.753	.020
Income: Prefer not to say	1.276	.845	1.927	.246
Education: Highschool or higher	**.741**	**.592**	**.928**	**.009**
Employed	.853	.686	1.061	.153
Laid-off/Furloughed	1.037	.747	1.441	.827
Condition limiting PA	**1.672**	**1.219**	**2.293**	**.001**
BMI ≤24	1.0			
BMI: 25-29.99	1.047	.838	1.308	.684
BMI: Obese: 30+	.880	.671	1.155	.357
Total isolation	**3.426**	**2.130**	**5.511**	**.000**
Minor/no Covid-19 risk percept	.978	.785	1.218	.842
Living with children	.837	.644	1.087	.182
Living with vulnerable	.756	.572	.998	.048
Access garden/balcony to exercise comfortably in	**.744**	**.594**	**.932**	**.010**
Access green space within walking distance	.820	.667	1.008	.059
Smoker	1.097	.854	1.410	.468
Alcohol drinking frequency, never (ref)	1.0			
Weekly or less	.753	.567	.999	.049
More than weekly	.752	.578	.977	.033
Deteriorated psychological wellbeing	**1.403**	**1.154**	**1.706**	**.001**

Model was adjusted for all the variable sin the table as well as the time of enrolment and time when Covid-19 started to affect individuals in any way. Significant results are in bold.

Contact: Aleksandra Herbec a.herbec@ucl.ac.uk

Supplementary Table S5.4. Predicting MVPA increase of at least 30 min in the inactive sample (fully adjusted model, weighted data).

Characteristics and associations	aOR	95% CI		p
Female	**1.877**	**1.213**	**2.902**	**.005**
Age (in decades)	**.707**	**.599**	**.836**	**.000**
White ethnicity	1.391	.575	3.363	.464
Household income ≥50.000	1.0			
Income: <50 000 GBP	**.394**	**.205**	**.757**	**.005**
Income: Prefer not to say	**.208**	**.077**	**.560**	**.002**
Education: Highschool or higher	**2.116**	**1.296**	**3.456**	**.003**
Employed	**.516**	**.309**	**.861**	**.011**
Laid-off/Furloughed	1.380	.757	2.517	.293
Condition limiting PA	.478	.257	.887	.019
BMI ≤24	1.0			
BMI: 25-29.99	1.511	.917	2.490	.105
BMI: Obese: 30+	.616	.341	1.113	.108
Total isolation	.332	.133	.827	.018
Minor/no Covid-19 risk percept	1.001	.620	1.616	.997
Living with children	**2.290**	**1.225**	**4.283**	**.009**
Living with vulnerable	.936	.519	1.687	.825
Access garden/balcony to exercise comfortably in	.764	.482	1.209	.250
Access green space within walking distance	.960	.622	1.481	.853
Smoker	.979	.622	1.542	.926
Alcohol drinking frequency, never (ref)	1.0			
Weekly or less	1.028	.555	1.904	.931
More than weekly	1.234	.708	2.152	.459
Deteriorated psychological wellbeing	.888	.579	1.362	.585

Model was adjusted for all the variable sin the table as well as the time of enrolment and time when Covid-19 started to affect individuals in any way. Significant results are in bold.

Contact: Aleksandra Herbec a.herbec@ucl.ac.uk
