# Supplementary material for: Correlates of and changes in aerobic physical activity and strength training before and after the onset of COVID-19 pandemic in the UK: findings from the HEBECO study
Source: BMJ Open. 2022 Jun 21;12(6):e054029. doi: 10.1136/bmjopen-2021-054029 (PMC9226469; doi:10.1136/bmjopen-2021-054029)
Supplement: Author's manuscript [file bmjopen-2021-054029.draft_revisions.pdf]

BMJ Open is committed to open peer review. As part of this commitment we make the peer review history of every article we publish publicly available.

When an article is published we post the peer reviewers' comments and the authors' responses online. We also post the versions of the paper that were used during peer review. These are the versions that the peer review comments apply to.

The versions of the paper that follow are the versions that were submitted during the peer review process. They are not the versions of record or the final published versions. They should not be cited or distributed as the published version of this manuscript.

BMJ Open is an open access journal and the full, final, typeset and author-corrected version of record of the manuscript is available on our site with no access controls, subscription charges or pay-per-view fees (http://bmjopen.bmj.com).

If you have any questions on BMJ Open's open peer review process please email info.bmjopen@bmj.com

BMJ Open

Correlates of and changes in aerobic physical activity and strength training before and after the onset of COVID-19 pandemic in the UK – findings from the HEBECO study.

Journal:	*BMJ Open*
Manuscript ID	bmjopen-2021-054029
Article Type:	Original research
Date Submitted by the Author:	31-May-2021
Complete List of Authors:	Herbec, Aleksandra; University College London,<br>Schneider, Verena; University College London<br>Fisher, Abigail; University College London<br>Kale, Dimitra; University College London<br>Shahab, Lion; University College London<br>Lally, Phillippa; University College London
Keywords:	COVID-19, SPORTS MEDICINE, PUBLIC HEALTH

SCHOLARONE™
Manuscripts

I, the Submitting Author has the right to grant and does grant on behalf of all authors of the Work (as defined in the below author licence), an exclusive licence and/or a non-exclusive licence for contributions from authors who are: i) UK Crown employees; ii) where BMJ has agreed a CC-BY licence shall apply, and/or iii) in accordance with the terms applicable for US Federal Government officers or employees acting as part of their official duties; on a worldwide, perpetual, irrevocable, royalty-free basis to BMJ Publishing Group Ltd ("BMJ") its licensees and where the relevant Journal is co-owned by BMJ to the co-owners of the Journal, to publish the Work in this journal and any other BMJ products and to exploit all rights, as set out in our licence.

The Submitting Author accepts and understands that any supply made under these terms is made by BMJ to the Submitting Author unless you are acting as an employee on behalf of your employer or a postgraduate student of an affiliated institution which is paying any applicable article publishing charge ("APC") for Open Access articles. Where the Submitting Author wishes to make the Work available on an Open Access basis (and intends to pay the relevant APC), the terms of reuse of such Open Access shall be governed by a Creative Commons licence – details of these licences and which Creative Commons licence will apply to this Work are set out in our licence referred to above.

Other than as permitted in any relevant BMJ Author's Self Archiving Policies, I confirm this Work has not been accepted for publication elsewhere, is not being considered for publication elsewhere and does not duplicate material already published. I confirm all authors consent to publication of this Work and authorise the granting of this licence.

For peer review only

Title: Correlates of and changes in aerobic physical activity and strength training before and after the onset of COVID-19 pandemic in the UK – findings from the HEBECO study.

Authors: Aleksandra Herbec[1,2*], Verena Schneider[1], Abi Fisher[1], Dimitra Kale[1], Lion Shahab[1], Phillippa Lally[1].

1 Department of Behavioural Science and Health, University College London, UK

2 Clinical, Educational and Health Psychology, University College London, UK

*contact: Aleksandra Herbec a.herbec@ucl.ac.uk

Pre-registration: **https://osf.io/spdtb**

For peer review only

Tables are at the end of the manuscript

Abstract

Objectives: Understanding changes in moderate to vigorous aerobic physical activity (MVPA) and muscle-strengthening activity (MSA) from before to after (pre-/post-) the onset of the Covid-19 pandemic in the UK (first lockdown) and their correlates can inform interventions.

Methods: Cross-sectional analysis of retrospective and concurrent data on MVPA/MSA pre- and post-Covid-19 (until 14th June 2020) among 2,657 UK adults. The associations between socio-demographic and health characteristics, MVPA/MSA pre-Covid-19, living and exercise conditions and meeting WHO recommended levels for MVPA/MSA/both (vs meeting neither), and changes in MVPA/MSA from pre- to post-Covid-19 following stratification for pre-Covid-19 MVPA/MSA levels were evaluated.

Results: A third of adults maintained (30.4%), decreased (36.2%) or increased (33.4%) their MVPA levels post-Covid-19. For MSA, the percentages were 61.6%, 18.2%, and 20.2%, respectively. MVPA increased or decreased by an average of 150min/week, and MSA by 2 days/week. Meeting both MSA+MVPA recommendations during lockdown (vs. meeting neither) was positively associated with meeting MVPA+MSA pre-lockdown (aOR=16.11,95%CI=11.24-23.07), and post-16-years of age education (aOR=1.57,1.14-2.17), and negatively associated with being obese (aOR=0.49,0.33-0.73), older age (65+ vs ≤34; aOR=.53,.32-.87), and annual household income <50.000GBP (vs ≥50.000GBP; aOR=0.65,0.46-0.91). The odds for decreasing MVPA were significantly lower for white ethnicity, post-16-years of age education, access to garden/balcony, and higher for those who were in total isolation. The odds for decreasing MSA were significantly higher for those who were overweight or obese.

Conclusion: Aerobic and strength training were differently impacted during the first UK lockdown, with poorer outcomes associated with older age, lower education, and higher body mass index.

Keywords: aerobic activity, strength training, covid-19, UK, cross-sectional, changes, MSA, MVPA

For peer review only

Article summary

- At the start of the Covid-19 pandemic, a third of UK adults decreased and a third increased moderate-to-vigorous physical activity; the corresponding proportions for muscle strength training were 18% and 20%.
- Adults with higher body mass index were at risk of not meeting WHO recommendations for physical activity, and at decreasing muscle strength training in this period.
- Those with lower incomes, no post-16-years of age education, ethnic minority background and living in total isolation had poorer physical activity outcomes, while older adults were more likely to maintain inactivity from before to start of the pandemic.

Strengths and limitations of this study

- This study contributes novel data on the unique correlates and o increases and decreases in two types of physical activity - moderate-to-vigorous physical activity and muscle strength training, during the initial stages of the Covid-19 pandemic.
- This was a cross-sectional and self-reported study among a convenience sample, but we minimised biases by weighing data using UK Census data and testing robustness of findings in sensitivity analyses.

For peer review only

Introduction

Physical activity has been negatively affected by the Covid-19 pandemic, which could have major implications for general health and Covid-19 outcomes[1,2,3]. As the Covid-19 pandemic continues and new restrictions can be introduced with the new waves of the pandemic, it is paramount to understand how physical activity, and especially aerobic and muscle-strength training, were affected during the initial stages of the lockdown, and which groups in the society may require most support.

Adults who engage in less than 30min of moderate physical activity per week are considered to be inactive [4,5]. For best health outcomes adults are recommend to engage in moderate-to-vigorous intensity aerobic physical activity (MVPA; i.e. activities that increase heart rate and make one feel warmer) for at least 150 minutes/week, as well as in muscle-strengthening activity (MSA; e.g., strength/resistance training) for at least two days/week[6]. MVPA and MSA both independently and combined lower morbidity and mortality[7-11,12]. Importantly, improving MVPA and MSA requires different strategies and interventions at both population and individual levels, thus necessitating dedicated research into them [13].

The Covid-19 pandemic [13] and the social distancing measures introduced in the UK and other countries have limited many opportunities to engage in physical activity[1413]. The first national lockdown in the UK was introduced on 23rd March 2020. As part of the lockdown measures, sports centres and gyms were temporarily closed and team sports banned, while access to outdoors spaces (e.g. remote national parks) was greatly reduced (i.e. some parks were closed or were only available to local visitors). On the other hand, certain lockdown measures introduced in the UK could facilitate exercising, including the introduction of the furlough scheme and the increased prevalence of remote working that offered greater flexibility in scheduling and engaging in exercising at home or in local parks [13]. Furthermore, exercising outdoors was listed as one of the few activities that were still permitted even during the strictest lockdown in the UK, alongside shopping for essential items or medicine and going to work.

A recent systematic review [15] showed that majority of studies to date reported declines in different measures of physical activity and increases in sedentary behaviour during the Covid-19 pandemic[15,16]. However, some studies found that a small minority of adults increased their activity[17], suggesting it is important to identify factors associated with both the detrimental and beneficial changes to activity levels. Female gender, lower income, older age, health conditions, perceived risks, and poorer mental health, and non-white ethnicity [18,19] [17,20-23] were associated with lower physical activity levels. However, few studies accounted for other factors that could be relevant during the pandemic, such as having access to indoor and outdoor exercising space or the household makeup[17].

Furthermore, research on how the Covid-19 pandemic has affected physical activity to date often focused on moderate-to-vigorous physical activity (MVPA), did not assess muscle-strengthening activity (MSA), failed to distinguish between MVPA and MSA[17,24,15,25], or assessed only perceived changes to physical activity levels with respect to pre-pandemic levels [26,27]. Moreover, few studies that assessed and reported declines in MVPA and MSA, for example among adults in Russia [28] and in Italy [29], did not report on factors associated with these specific changes.

For peer review only - http://bmjopen.bmj.com/site/about/guidelines.xhtml

In order to identify groups in the population that may require targeted interventions for physical activity during future periods of lockdowns or restrictions to physical activity, it is important to research the patterns and factors associated with changes in the levels of both MVPA and MSA that are recommended by the WHO.

Aims

We characterised separately and combined MVPA and MSA levels during the periods *before* and *after* (pre- /post-) the Covid-19 pandemic had started in the UK, with the latter covering the period of the first strictest UK lockdown up until 14th June 2020. The research questions (RQs) were:

- RQ1: What were the levels and changes in MVPA and MSA among UK adults from pre- to post- Covid-19?
- What socio-demographic, environmental and health factors were associated with:
  RQ2: meeting WHO recommendations for MVPA and MSA post-Covid-19?
  RQ3: (i) decrease in MVPA among those who were active, and (ii) increase in MVPA among those who were inactive from pre- to post-Covid-19?
  RQ4: (i) decrease in MSA among those who were active, and (ii) increase in MSA among those who were inactive, from pre- to post-Covid-19?

Methods

Study Design

The study involved cross-sectional data analysis from the baseline online survey of the HEalth BEhaviours during the COvid-19 pandemic (HEBECO) study (https://osf.io/sbgru/). Data were collected using REDCap software at UCL (Harris et al, 2019). The protocol for the present data analysis was preregistered (https://osf.io/ejghs/). Departures from the protocol are explained in Supplementary Materials 1. The results from all the pre-registered analyses not reported here are available elsewhere: https://osf.io/2ujxq/. The reporting follows the STROBE guidelines.[30]

Participants and Procedures

Recruitment involved a UK-wide campaign including paid and unpaid posts on social media and information shared through the networks of Cancer Research UK, Public Health England, other charities, sports clubs, universities, and local authorities. Interested participants were directed to study website where they were shown participant information sheet and provided informed consent, and then completed the online survey. Participants with complete data on the measures of interest for this study, and who enrolled into the baseline between 5th May 2020 (start of the data collection for all study variables, during the first UK lockdown) until 14th June 2020 (the last day prior to easing of restrictions of the first UK lockdown) were included in the analytical sample.

Patient and Public Involvement

For peer review only

Patients or public were not involved in the HEBECO baseline design, but the survey was shared with researchers at CRUK and Public Health England Behavioural Insights in order to collect their feedback and suggestions for question and answer wording, where relevant (e.g. when questions were not validated). Participants in the HEBECO study were encouraged to leave comments about specific behaviours and the survey itself and these were incorporated into subsequent follow-up surveys (not the present study), where possible and relevant.

Measures

For the wording of all measures see https://osf.io/bja7g/ and Supplementary Materials 2. At baseline participants indicated the time when their lives started to be affected by the Covid-19 pandemic in any way, which was used as an anchor to assess before/since (pre-/post-Covid-19) MVPA and MSA. Almost half (44.0%, weighted) of adults selected the second half of March, followed by 26.9% who selected the first half of March.

Meeting WHO recommendations for MVPA and MSA

The pre-/post-Covid-19 MVPA and MSA were measured using the same set of questions[12,31]. For MVPA, participants were first asked "In the month Before/Since Covid-19, on average, HOW MANY TIMES PER WEEK did you do at least 15 MINUTES or more of moderate or vigorous AEROBIC PHYSICAL ACTIVITY? Examples: brisk walk, jogging, dancing, cycling for recreation or commute, swimming, team or racket sports." The answers were capped at 14+, equivalent to at least twice per day. The 15 minute period was selected as it corresponds to the minimum amount of PA needed for mortality reduction and extending lifespan[32]. Those who engaged in at least one such session were asked "In the month BEFORE/Since COVID-19, HOW LONG (in MINUTES) was your average session of moderate or vigorous AEROBIC PHYSICAL ACTIVITY? Do NOT include strength training." Total weekly MVPA was computed by multiplying the session number by the minutes.

MSA activity was assessed by a single question: "Before/Since Covid-19, on average, on HOW MANY DAYS PER WEEK did you do STRENGTH TRAINING? Examples: Pilates, push-ups, squats, yoga, and exercises involving free weights, weight machines or elastic band." The answers were capped at 4+ given that the recommendations were for 2 days of MSA per week.

Data on MVPA and MSA were used to categorise participants, separately for pre-/post-Covid-19, into four groups based on whether they met the WHO recommendations for MSA (at least 2 days/week) and MVPA (≥150 minutes/week)[6]: meeting neither, meeting MSA only, meeting MVPA only, or meeting recommendations for both MSA and MVPA.

Change in MVPA and MSA

Changes in MVPA (RQ 3) and MSA (RQ 4) were calculated by subtracting the pre-Covid-19 levels of MSA or MVPA from the post-Covid-19 levels. Participants were categorised into: maintenance (change in MVPA <20 min; change in MSA=0), decrease (MVPA by ≥20min; MSA by ≥1 day) or increase in activity (MVPA by ≥20min; MSA by ≥1 day). Dichotomous variables were then creased: decrease (vs maintain/increase) and increase (vs maintain/decrease) in MVPA and MSA.

For peer review only

To minimise the bias due to ceiling and floor effects when assessing changes to MVPA and MSA, the analyses for RQs 3 and 4 were conducted after stratifying for pre-Covid-19 MVPA and MSA . Participants were categorised into: inactive MVPA (<30min of MVPA/week[4,5]) or MSA (0 days of MSA/week) and active (≥30 min/week of MVPA; and ≥ 1 day/week of MSA) pre-Covid-19.

Participants who were active post-Covid-19 were also asked “Do you do the same FORM of exercise as you did before the COVID-19? (even if it is in a different location)?” The answer options were: None of the same/some of the same/about half the same/mostly the same/exactly the same.

Explanatory factors and correlates

Socio-demographic characteristics and living conditions assessed were: gender (participants were asked to select which answer they identify with most, and the answers were dichotomised into: female/all other), ethnicity (white/non-white); education (post-16-years of age/other); employed (yes/no); furloughed/laid off (yes/no); income (low-middle <50 000 GBP/high ≥50 000 GBP/prefer-not-to-say); age (in decades was entered as a continuous variable where it met assumptions, or as a 3-level categorical variable: ≤34, 35-64, 65+).

We assessed health behaviours, health and living conditions that could impact on MVPA or MSA levels: self-reports of any condition that limited physical activity (yes/no); body mass index (BMI[31]: normal and underweight ≤24.99/overweight 25-29.99/ obese ≥30); smoking status (current smoker vs not)[33]; weekly frequency of alcohol drinking[34]; deterioration in psychological wellbeing from pre- to post-Covid-19 (yes/no); participants’ perceived risk of Covid-19 to their health (‘no or minor risk’/other); access to a garden or balcony big enough to exercise comfortably (yes/no); access to a public park/green space that is within a walking distance and open during Covid-19 (yes/no); living with children aged ≤15 (yes/no); living with vulnerable persons (persons over the age of 70, in poor health, or who may be vulnerable to Covid-19; yes/not). Participants were asked about the status of their isolation at the time of completing the survey: “Which type of COVID-19 induced isolation are you experiencing?” (i) Total isolation/quarantine (not leaving the house for ANY reasons, not even to buy groceries or medications or to exercise); (ii) Some isolation (not leaving the house EXCEPT to buy essential items, such as groceries or medication or to exercise); (iii) General isolation but still go out to work (still go out to work and to buy essential items, such as groceries or medications or to exercise); (iv) No isolation (I am free to leave the house whenever I like, including participating in social gatherings or group sports, going to a bar or restaurant, travelling for leisure). The answers were dichotomized into: (i) total isolation/ (ii-iv) all other.

The regression models described below included two time co-variates to account for weather changes: enrolment time (up until 15th May, the second half of May, the first half June), and time when Covid-19 started to affect individuals (before mid-March/later) as these could affect exercise levels.

Analysis

Analyses were conducted in SPSS 26 with the data weighted using the 2018 Census and APS mid-year estimates for age, gender, ethnicity, country of living and household income. The analysis used weights trimmed to top 98th percentile to minimise the impact of extremely high weights [35].

For peer review only - http://bmjopen.bmj.com/site/about/guidelines.xhtml

Differences between the included and excluded participants were assessed with chi-squared for categorical and t-tests for continuous data.

For RQ1, descriptive statistics were computed to characterise the levels of MVPA, MSA, and inactivity pre-/post-Covid-19 and changes in exercise form. For RQ2 univariate and fully adjusted multinomial regression models were computed. For RQ3 and RQ4 the sample was stratified using pre-Covid-19 MVPA or MSA levels to assess outcomes of interest using univariate and adjusted logistic regression models. All independent variables were entered into the models together as they were selected due to their previously established or theoretical importance. Sensitivity analyses involved replicating the analyses using unweighted data to check for the robustness of the results, and for RQ3 using differ cut-off values of 15mins and 30mins. Family-wise error was corrected for by using the Benjamini-Hochberg procedure separately for each research question (Benjamini and Hochberg, 1995).

Results

Out of 2992 participants who were included in the HEBECO baseline sample, 2,657 adults (weighted N=2,442) had complete data for this study and were included in the analytical sample. Table 1 present comparisons of the excluded and included sample. The included weighted sample comprised 52% females, 90.5% of white ethnicity, and 67.0% with high school education or higher.

The sections below summarise the significant results for the weighted fully adjusted analyses. Results from univariable analyses are reported in Supplementary Materials 3, and from sensitivity analyses in Supplementary Materials 4 and 5, and have not changed the conclusions.

Research Question 1: Changes in MVPA and MSA pre- to post-Covid-19.

Table 2 presents data on physical activity levels before and since Covid-19. Pre-Covid-19 17.6% of adults engaged in no MVPA and MSA, 55.3% engaged in no MSA, and 19.4% engaged in no MVPA. Just under 15% of adults met the recommended levels of both MVPA and MSA pre- and post- Covid-19. The proportion of those who had no MVPA or MSA activity increased minimally (17.4% to 22.1%) which was primarily driven by declines in MVPA. Among those who were active post-Covid-19, 41.5% continued with the mostly or exactly activity form as pre-Covid-19.

From before to since Covid-19 similar proportions of adults maintained (30.4%), decreased (36.2%) or increased (33.5%) their weekly MVPA. Among those who were active pre-Covid-19, 46.7% decreased MVPA. The decrease was of on average 219min/week. Among those who were inactive pre-Covid-19, 29.7% increased MVPA, with an average increase of 144min/week.

Maintenance of MSA was relatively more common (61.6%), with 18.2% decreasing and 20.2% increasing the number of days they engaged in MSA. Among those who were active pre-Covid-19 (40.2%), 45.1% decreased MSA, with an average decrease of 1.9 days/week. Among those who engaged in no MSA pre-Covid-19 (59.8%), 16.4% increased, with an average increase of 2.5 days/week.

Research Question 2: Predictors of meeting WHO recommendations

For peer review only - http://bmjopen.bmj.com/site/about/guidelines.xhtml

For peer review only

Table 3 presents results from fully adjusted models. Being aged 65+, having a lower pre-Covid-19 household income, having a condition limiting physical activity, being obese, living in total isolation and deterioration in psychological wellbeing were significantly associated with lower odds, and having at least high school education or meeting the WHO recommended levels of MVPA, MSA or both pre-Covid-19, were significantly associated with greater odds of meeting both MVPA and MSA WHO guidelines since Covid-19, as compared with not meeting either.

Having a condition that limited physical activity, being in total isolation and deterioration in psychological wellbeing were significantly associated with lower odds, and meeting the WHO recommended levels of MVPA or both MVPA and MSA was significantly associated with greater odds of meeting MVPA only post-Covid-19. Being aged 35-64, and having obesity (BMI≥30) was associated with lower odds, while higher education, and meeting both MVPA and MSA or only MSA recommendations pre-Covid-19 was associated with meeting the recommendations for MSA only post-Covid-19.

RQ 3: Associations with changes in MVPA

Older, white participants with at least high school education and access to a garden/balcony to exercise comfortably in were significantly less likely to decrease MVPA activity (Table 4). Those with conditions limiting PA, living in total isolation and experiencing a deterioration in psychological wellbeing during lockdown were significantly more likely to decrease MVPA activity from pre-Covid levels.

White ethnicity, being employed during Covid-19, and living in total isolation were significantly associated with lower odds of increasing MVPA among this group. At least high school education and living with children was significantly associated with higher odds of increasing MVPA activity.

RQ 4: Associations with changes in MSA

Being employed during lockdown and being overweight (BMI 25-29.99) or obese (BMI ≥30) was significantly associated with higher odds of decreasing MSA activity. Older age (35-64, and 65+) and deterioration in psychological wellbeing were significantly associated with lower odds of increasing MSA. See Table 5 for details.

Discussion

This study shows that the first Covid-19 lockdown in the UK affected differently the levels of moderate-to-vigorous physical activity (MVPA) and muscle-strengthening activity (MSA), with different factors associated with engagement in and changes in these two activity types. MVPA and MSA declined among a substantial proportion of UK adults during the first lockdown, which is in line with much research to date that also showed declines across different measures of physical activity[15,18]. However, the present findings also demonstrate that a substantial minority of UK adults maintained or increased their MVPA or MSA. These findings underline the importance of measuring MVPA and MSA, and their correlates, separately.

For peer review only - http://bmjopen.bmj.com/site/about/guidelines.xhtml

Meeting WHO recommendations for MVPA and MSA pre- and post-Covid-19

Adherence to WHO recommendations for both MVPA and MSA pre- and post-Covid-19 was low – at about 15%, which is lower than 26% reported in the Health Survey for England (HSE) for 2012-2016[36]. In comparison to the findings from HSE, fewer adults in this study met MVPA only recommendations (21% vs 36%), but more met MSA only recommendations (12% vs 1%). The current MSA levels are in line with other epidemiological studies[7,37]. The differences between the HEBECO and HSE findings could be due to the differences in the measures of MSA and MVPA used, participant characteristics (e.g. in comparison to the HSE study[36], the HEBECO sample included more educated adults who may be more aware of the benefits of MSA), or reflect genuine changes to the patterns of physical activity in the UK across time. As found in other studies [36,38], older adults (aged 65+), adults who were obese, those with pre-Covid-19 household income below 50,000 GBP and who had conditions limiting physical activity were at risk of not meeting the WHO recommendations for both MVPA and MSA. These groups should be target by future interventions.

Moreover, meeting the recommendations for MVPA and MSA before Covid-19 was strongly predictive of meeting them post-Covid-19. Additionally, adults who remained active tended to maintain at least similar levels and forms of the exercises as pre-pandemic. Thus, as UK adults remain relatively consistent in their behaviour even during the lockdown, this study highlights the need to especially support those with low activity levels to develop healthy exercising routines.

Changes to MVPA and MSA levels from pre- to the post-Covid-19 pandemic start

Although the group level data suggest little change in physical activity pre- to post-Covid-19, especially in MSA, about a third of adults decreased and another third increased their MVPA from pre- to post-Covid-19. Those who changed MVPA decreased or increased their MVPA levels by over three and two hours per week, respectively. Those who changed MSA either gained or lost two days of MSA per week. These changes are substantial and likely to affect health outcomes.

Decreases in MVPA were found among other adults samples in the UK[17], Italy [24] and Spain[25]. However, the findings that over 70% of UK adults maintained or increased their MVPA could be at least partially attributed to the UK government's consistent lockdown policies that allowed leaving the house for exercising, which placed UK in contrast to other countries[25,39].

The maintenance of MSA levels could be at least partially explained by the low pre-Covid-19 MSA levels. Additionally, many MSA exercises can be performed with no equipment and at one's home (e.g. Pilates, push-ups) and thus may be less affected by social distancing measures. This is further supported by the present findings that total isolation (i.e. not leaving the house for any reason) was associated with MVPA but not MSA levels.

Factors associated with changes and maintenance of MVPA and MSA post-Covid-19

Older adults were more likely to maintain their pre-Covid-19 MVPA and MSA levels, including inactivity. Due to more established routines and lower baseline physical activity this group might have been less affected by the lockdown restrictions, such as gym closures or team sports bans[40].

For peer review only

MVPA inactivity was more commonly maintained by those who were employed or living in total isolation. Such adults likely had fewer opportunities or flexibility to exercise. Additionally, those who had a health condition limiting physical activity and those living in total isolation tended to decrease MVPA. Factors that were associated with maintaining MVPA activity post-Covid-19 were white ethnicity, higher education, and having a garden or balcony large enough to exercise.

Increasing MVPA levels among those who were previously inactive was most common among those with higher education, which is in line with prior studies [41], and those who lived with children. Caring for children might have promoted some forms of MVPA, including outdoors, particularly as parents became responsible for children's physical education during school lockdown closures.

In terms of changes in MSA levels, older adults were at higher risk of maintaining MSA inactivity, while adults with higher BMI levels were at greater risk of decreasing MSA from pre- to post-Covid-19. Adults with higher BMI reported lower levels of physical activity during lockdowns in other studies as well.[26] Therefore, both older adults and those with higher BMI should be among priority groups targeted with MSA interventions, particularly as MSA can bring important clinical benefits to these two groups[42-45]. Other priority groups for MSA training are those with lower household income and lower educational attainment.

Finally, as found previously[22], psychological wellbeing was associated with physical activity levels. In the present study deterioration in psychological wellbeing was associated with not meeting guidelines for MVPA or decreasing MVPA levels, but not with MSA. Due to the cross-sectional design causality cannot be assumed, as poorer psychological wellbeing (e.g. lower mood, anxiety, stress), can be both a predictor and a consequence of low exercise levels[46,47]. However, interventions aimed at improving physical activity are likely to improve mental health as well[46-48].

Strength and limitation

The study is among a few that assessed the levels and changes to both MVPA and MSA during the first UK lockdown. The study drew on previously used measures of MVPA and MSA[49], which were supplemented by images to clarify exercise types. This study also benefits from a large list of correlates and covariates being measured to control for confounding. A number of sensitivity analyses were conducted to test the robustness of findings. The key limitations are that this was a cross-sectional study among self-selected sample that relied on self-report and recall that are prone to bias, which could lead to over-reporting of physical activity, opening a possibility that the wider population had an even poorer physical activity profile during the pandemic. A small sample (11%) of all HEBECO baseline participants were exclude from the present analysis due to incomplete data (e.g. attrition from the survey before reporting physical activity). Some of the characteristics of the excluded participants could be associated with greater physical activity (i.e. younger age, non-female gender, lower BMI), while others were markers of lower socio-economic status and could be associated with lower physical activity levels, such as not being in employment, not having access to garden or balcony, being a smoker, or being of non-White ethnicity. The reported data are weighted using census data to increase their representativeness of the UK population. On balance, the present findings may be reflective of the changes in physical activity happening at the population level in the UK. Finally, the unfolding of the Covid-19 pandemic has coincided with season change from winter to spring. Without a true baseline from the same period in 2019 it is not possible to tease apart the effect of weather change from that of the pandemic.

For peer review only - http://bmjopen.bmj.com/site/about/guidelines.xhtml

Implications

This study adds to the growing body of literature that emphasises the importance of researching MSA in addition to the more commonly assessed MVPA [7]. It also shows that although the first UK lockdown adversely affected many individuals, a considerable proportion of the adults managed to maintain or even improved MVPA and MSA. These findings suggest that future interventions and policies should not only aim to prevent deterioration in physical activity, but also try to capitalise on the opportunities brought by lifestyle disruption to support increases in activity levels. We now have at our disposal many interventions and tools, including those which are digital or technology-facilitated and thus could be offered at relatively low cost to large target populations, all whilst adhering to even the strictest lockdown measures. Efforts should focus on identifying acceptable and effective ways of delivering interventions for both MVPA and MSA, especially given the ongoing challenges caused by the Covid-19 pandemic.

Conclusions

The findings point to social inequalities in how the first lockdown in the UK has affected physical activity levels, with differential impact on aerobic and strength training. Dedicated interventions are needed to support MVPA and MSA especially among those who have low activity levels, those who are older, have lower income, and have higher body mass index.

For peer review only

Footnotes

- Contributorship statement: AH conceived the idea in consultation with PL, AB and LS. AH and VS analysed the data. All authors interpreted the data. AH drafted the first version of the manuscript. All authors critically revised the manuscript and approved the final draft before submission.

- **Funding** This project is partially funded by an ongoing Cancer Research UK Programme Grant to UCL Tobacco and Alcohol Research Group (C1417/A22962) and by SPECTRUM, a UK Prevention Research Partnership Consortium (MR/S037519/1).

- **Competing interests** None declared.
- **Patient and public involvement** Patients and/or the public were not involved in the design, or conduct, or reporting, or dissemination plans of this research.
- **Patient consent for publication** Obtained.
- **Ethics approval** The study was approved by the by UCL Research Ethics Committee at the UCL Division of Psychology and Language Sciences (PaLS) (CEHP/2020/579; as part of the larger programme of work: The optimisation and implementation of interventions to change behaviours related to health and the environment).
- **Data Sharing Statement:** Study and additional data can be accessed by contacting lion.shahab@ucl.ac.uk and a.herbec@ucl.ac.uk

- **Acknowledgements:** We are grateful to all participants who have been supporting our research. We would like to thank Public Health England, and particularly members of the Behavioural Insights at Public Health England for providing feedback on the survey wording. We would like to thank Public Health England, Cancer Research UK, local authorities, Mayors' offices, as well as charities and other organisations in the UK, including the Asthma UK and British Lung Foundation Partnership, for supporting our recruitment campaign.

References

1. Warburton DER, Bredin SSD. Health benefits of physical activity: a systematic review of current systematic reviews. *Curr. Opin. Cardiol.* 2017;32(5):541-556.
2. Woods JA, Hutchinson NT, Powers SK, et al. The COVID-19 pandemic and physical activity. *Sports Medicine and Health Science.* 2020;2(2):55-64.
3. Shur NF, Johns D, Kluzek S, Peirce N. Physical inactivity and health inequality during coronavirus: a novel opportunity or total lockdown? *BMJ Open Sport & Exercise Medicine.* 2020;6(1):e000903.
4. Public Health England. Physical inactivity levels in adults aged 40 to 60 in England. 2017; Accessed on 20th September 2020 from: https://www.gov.uk/government/publications/physical-inactivity-levels-in-adults-aged-40-to-60-in-england/.
5. Health Survey for England. Health Survey for England 2016 - Physical activity in adults. 2017; Accessed on 20th Sept 2020 from: http://healthsurvey.hscic.gov.uk/media/63730/HSE16-Adult-phy-act.pdf
6. WHO. Physical activity. . 2018; Accessed on 20th November 2020 from https://www.who.int/news-room/fact-sheets/detail/physical-activity.

For peer review only - http://bmjopen.bmj.com/site/about/guidelines.xhtml

7. Bennie JA, Shakespear-Druery J, De Cocker K. Muscle-strengthening Exercise Epidemiology: a New Frontier in Chronic Disease Prevention. *Sports medicine - open.* 2020;6(1):40.
8. Kamada M, Shiroma EJ, Buring JE, Miyachi M, Lee IM. Strength Training and All-Cause, Cardiovascular Disease, and Cancer Mortality in Older Women: A Cohort Study. *Journal of the American Heart Association.* 2017;6(11).
9. Stamatakis E, Lee IM, Bennie J, et al. Does Strength-Promoting Exercise Confer Unique Health Benefits? A Pooled Analysis of Data on 11 Population Cohorts With All-Cause, Cancer, and Cardiovascular Mortality Endpoints. *American journal of epidemiology.* 2018;187(5):1102-1112.
10. Zhao G, Li C, Ford ES, et al. Leisure-time aerobic physical activity, muscle-strengthening activity and mortality risks among US adults: the NHANES linked mortality study. *Br. J. Sports Med.* 2014;48(3):244-249.
11. Zhao M, Veeranki SP, Magnussen CG, Xi B. Recommended physical activity and all cause and cause specific mortality in US adults: prospective cohort study. *BMJ (Clinical research ed.* 2020;370:m2031.
12. Bennie JA, De Cocker K, Teychenne MJ, Brown WJ, Biddle SJH. The epidemiology of aerobic physical activity and muscle-strengthening activity guideline adherence among 383,928 U.S. adults. *The international journal of behavioral nutrition and physical activity.* 2019;16(1):34.
13. Dwyer MJ, Pasini M, De Dominicis S, Righi E. Physical activity: Benefits and challenges during the COVID-19 pandemic. *Scand. J. Med. Sci. Sports.* 2020;30(7):1291-1294.
14. Ricci F, Izzicupo P, Moscucci F, et al. Recommendations for Physical Inactivity and Sedentary Behavior During the Coronavirus Disease (COVID-19) Pandemic. *Frontiers in public health.* 2020;8:199.
15. Stockwell S, Trott M, Tully M, et al. Changes in physical activity and sedentary behaviours from before to during the COVID-19 pandemic lockdown: a systematic review. *BMJ Open Sport Exerc Med.* 2021;7(1):e000960.
16. Ammar A, Brach M, Trabelsi K, et al. Effects of COVID-19 Home Confinement on Eating Behaviour and Physical Activity: Results of the ECLB-COVID19 International Online Survey. *Nutrients.* 2020;12(6).
17. Rogers NT, Waterlow N, Brindle HE, et al. Behavioural change towards reduced intensity physical activity is disproportionately prevalent among adults with serious health issues or self-perception of high risk during the UK COVID-19 lockdown. . *MedRxiv* 2020.
18. Sport England. Active Lives Adult Survey: November 2019/20 Report. 2021.
19. Dunton GF, Wang SD, Do B, Courtney J. Early effects of the COVID-19 pandemic on physical activity locations and behaviors in adults living in the United States. *Preventive medicine reports.* 2020;20:101241.
20. Meyer J, McDowell C, Lansing J, et al. Changes in physical activity and sedentary behaviour due to the COVID-19 outbreak and associations with mental health in 3,052 US adults. *Cambridge Open Engage.* 2020.
21. Smith L, Jacob L, Butler L, et al. Prevalence and correlates of physical activity in a sample of UK adults observing social distancing during the COVID-19 pandemic. *BMJ Open Sport & Exercise Medicine.* 2020;6(1):e000850.
22. Jacob L, Tully MA, Barnett Y, et al. The relationship between physical activity and mental health in a sample of the UK public: A cross-sectional study during the implementation of COVID-19 social distancing measures. *Mental health and physical activity.* 2020;19:100345.
23. Constandt B, Thibaut E, De Bosscher V, Scheerder J, Ricour M, Willem A. Exercising in Times of Lockdown: An Analysis of the Impact of COVID-19 on Levels and Patterns of Exercise among Adults in Belgium. *International journal of environmental research and public health.* 2020;17(11).
24. Maugeri G, Castrogiovanni P, Battaglia G, et al. The impact of physical activity on psychological health during Covid-19 pandemic in Italy. *Heliyon.* 2020;6(6):e04315.

For peer review only

25. López-Bueno R, Calatayud J, Andersen LL, et al. Immediate Impact of the COVID-19 Confinement on Physical Activity Levels in Spanish Adults. *Sustainability.* 2020;12(14):5708.
26. Robinson E, Boyland E, Chisholm A, et al. Obesity, eating behavior and physical activity during COVID-19 lockdown: A study of UK adults. *Appetite.* 2021;156:104853.
27. Spence JC, Rhodes RE, McCurdy A, Mangan A, Hopkins D, Mummery WK. Determinants of physical activity among adults in the United Kingdom during the COVID-19 pandemic: The DUK-COVID study. *British journal of health psychology.* 2021;26(2):588-605.
28. Kontsevaya AV, Mukaneeva DK, Myrzamatova AO, Okely AD, Drapkina OM. Changes in physical activity and sleep habits among adults in Russian Federation during COVID-19: a cross-sectional study. *BMC public health.* 2021;21(1):893.
29. Fuzeki E, Schroder J, Carraro N, et al. Physical Activity during the First COVID-19-Related Lockdown in Italy. *International journal of environmental research and public health.* 2021;18(5).
30. von Elm E, Altman DG, Egger M, et al. Strengthening the Reporting of Observational Studies in Epidemiology (STROBE) statement: guidelines for reporting observational studies. *BMJ (Clinical research ed.* 2007;335(7624):806-808.
31. Churilla JR, Johnson TM, Richardson MR, Williams BD, Rariden BS, Boltz AJ. Mode of physical activity participation by body mass index: 2015 behavioural risk factor surveillance system. *Res. Sports Med.* 2018;26(2):147-157.
32. Wen CP, Wai JPM, Tsai MK, et al. Minimum amount of physical activity for reduced mortality and extended life expectancy: a prospective cohort study. *The Lancet.* 2011;378(9798):1244-1253.
33. Kaczynski AT, Manske SR, Mannell RC, Grewal K. Smoking and Physical Activity: A Systematic Review. *American Journal of Health Behavior.* 2008;32(1).
34. Leasure JL, Neighbors C, Henderson CE, Young CM. Exercise and Alcohol Consumption: What We Know, What We Need to Know, and Why it is Important. *Frontiers in psychiatry.* 2015;6:156.
35. Potter FA. Study of Procedures to Identify and Trim Extreme Sample Weights. . *Proceedings of the Survey Research Methods Section, American Statistical Association.* 1990:225-230.
36. De Cocker K, Teychenne M, White RL, Bennie JA. Adherence to aerobic and muscle-strengthening exercise guidelines and associations with psychological distress: A cross-sectional study of 14,050 English adults. *Prev Med.* 2020;139:106192.
37. Bennie JA, De Cocker K, Smith JJ, Wiesner GH. The epidemiology of muscle-strengthening exercise in Europe: A 28-country comparison including 280,605 adults. *PloS one.* 2020;15(11):e0242220.
38. Salman A, Ukwaja KN, Alkhatib A. Factors Associated with Meeting Current Recommendation for Physical Activity in Scottish Adults with Diabetes. *International journal of environmental research and public health.* 2019;16(20).
39. DW. Coronavirus: What are the lockdown measures across Europe? 2020; Accessed 20th Sept 2020 from: https://www.dw.com/en/coronavirus-what-are-the-lockdown-measures-across-europe/a-52905137.
40. Sun F, Norman IJ, While AE. Physical activity in older people: a systematic review. *BMC public health.* 2013;13:449.
41. Shaw BA, Spokane LS. Examining the association between education level and physical activity changes during early old age. *J. Aging Health.* 2008;20(7):767-787.
42. Kraschnewski JL, Sciamanna CN, Poger JM, et al. Is strength training associated with mortality benefits? A 15year cohort study of US older adults. *Prev Med.* 2016;87:121-127.
43. Guizelini PC, de Aguiar RA, Denadai BS, Caputo F, Greco CC. Effect of resistance training on muscle strength and rate of force development in healthy older adults: A systematic review and meta-analysis. *Exp. Gerontol.* 2018;102:51-58.

For peer review only

44. Said MA, Abdelmoneem M, Almaqhawi A, Hamid Kotob AA, Alibrahim MC, Bougmiza I. Multidisciplinary approach to obesity: Aerobic or resistance physical exercise? *Journal of exercise science and fitness.* 2018;16(3):118-123.
45. Willis LH, Slentz CA, Bateman LA, et al. Effects of aerobic and/or resistance training on body mass and fat mass in overweight or obese adults. *J Appl Physiol (1985).* 2012;113(12):1831-1837.
46. Sharma A, Madaan V, Petty FD. Exercise for mental health. *Primary care companion to the Journal of clinical psychiatry.* 2006;8(2):106.
47. Mikkelsen K, Stojanovska L, Polenakovic M, Bosevski M, Apostolopoulos V. Exercise and mental health. *Maturitas.* 2017;106:48-56.
48. Callow DD, Arnold-Nedimala NA, Jordan LS, et al. The Mental Health Benefits of Physical Activity in Older Adults Survive the COVID-19 Pandemic. *Am. J. Geriatr. Psychiatry.* 2020;28(10):1046-1057.
49. Bennie JA, Pedisic Z, van Uffelen JG, et al. The descriptive epidemiology of total physical activity, muscle-strengthening exercises and sedentary behaviour among Australian adults--results from the National Nutrition and Physical Activity Survey. *BMC public health.* 2016;16:73.

For peer review only

Table 1. Sample characteristics (weighted) and comparison of participants who were included and excluded from the study (due to incomplete data).

	Included sample N=2442	Excluded sample N=350	*p*
Female	52.6%	48.7%	0.179
Age; M (SD)	50.0 (16.0)	42.7 (18.9)	<0.001
White ethnicity	90.5%	82.4%	<0.001
Household income ≥50.000	18.1%	15.5%	0.095
Income: <50 000 GBP	73.5%	73.1%	
Income: Prefer not to say	8.3%	11.5%	
Education: Highschool or higher	67.0%	69.1%	0.432
Employed	48.3%	40.7%	0.008
Laid-off/Furloughed	12.7%	15.2%	0.197
Condition limiting PA	16.3%	21.2%	0.035
BMI ≤24	43.2%	27.3%	<0.001
BMI: 25-29.99	35.1%	16.8%	
BMI: Obese: 30+	21.7%	8.4%	
BMI don't know/prefer not to say[a]	0%	47.6%	
Total isolation	7.8%	8.8%	0.541
Minor/no Covid-19 risk percept	33.3%	38.6%	0.069
Living with children	17.0%	15.7%	0.549
Living with vulnerable	15.1%	21.8%	0.001
Access garden/balcony	72.2%	62.6%	<0.001
Access green space	59.5%	52.4%	0.012
Time life affected by Covid-19 mid- March or sooner[b]	44.8%	53.3%	0.003
Enrolled from 1st June (ref)	4.4%	5.4%	0.404
Enrolled up until 15th May	50.1%	46.7%	
Enrolled second half of May	45.4%	47.9%	
Smoker	24.1%	38.9%	<0.001
Weekly alcohol drinking, none (ref)	21.8%	21.8%	0.920
Weekly or less	27.9%	26.9%	
More than weekly	50.3%	51.3%	
Deteriorated psychological wellbeing	54.1%	52.8%	0.708
Meeting WHO MVPA and MSA levels before Covid	15.6%	17.3%	0.218
Meeting only MVPA before	23.0%	22.0%	
Meeting only MSA before	13.0%	7.7%	
Meeting neither before	48.4%	53.0%	

[a]As per protocol, the participants who did not provide BMI data (e.g. they selected 'prefer not to say' or 'don't know' on the question on height or weight) but who provided other study data were to be included in the analyses, but were ultimately excluded due to having missing data on other variables. [b]Time that participant's life started to be affected by Covid-19 in any way

For peer review only

Table 2. Aerobic physical activity (MVPA) and strength training (MSA) before and since COVID-19.

	Before Covid-19[1]	Since Covid-19[1]
**No activity (0 sessions MVPA and 0 days MSA) % (N)**	17.6 (430)	22.1 (540)
**MVPA**		
MVPA 0 sessions % (N)	19.4 (473)	24.8 (604)
MVPA min/week among active; Median (IQR)	125 (210.0)	150 (220.0)
Mean (SD)	238.1 (424.0)	230.9 (398.8)
MVPA min/week among entire sample; Median (IQR)	90 (180.0)	90 (224.5)
Mean (SD)	196.2 (395.4)	179.8 (364.7)
**MSA**		
MSA % (N)		
0 das/week	55.3 (1557)	56.2 (1584)
1 day/week	11.1 (314)	9.6 (271)
2 days/week	10.4 (293)	7.9 (223)
3 days/week	9.0 (253)	7.6 (213
4+ days/week	7.0 (197)	11.3 (320)
MSA Median (IQR)	0 (2)	0 (2)
**Meet guidelines % (N)**		
Neither MVPA nor MSA	45.1 (1271)	44.6 (1256)
Meet MVPA (≥150min/week) only	21.3 (599)	21.2 (598)
Meet MSA (≥2 days/week) only	11.7 (330)	11.9 (336)
Meets both MVPA and MSA	14.5 (410)	14.8 (416)
**Change in form of exercise among those who exercise % (N)**		
None of the same	-	17.1 (331)
Some of the same	-	32.7 (632)
About half the same	-	8.7 (168)
Mostly the same	-	26.0 (503)
Exactly the same	-	15.5 (299)

For peer review only

For peer review only - http://bmjopen.bmj.com/site/about/guidelines.xhtml

Table 3. Predictors of meeting both, MVPA only and MSA only WHO guidelines since Covid-19 in comparison to not meeting WHO guidelines for MVPA and MSA (reference, n=1178). Results from fully adjusted models using all variables listed on weighted data with BH FDR correction of p-values (significant in bold).

	Both MVPA and MSA (n=388)				MVPA only (n=570)				MSA only (n=306)			
	aOR	95% CI		*p*	aOR	95% CI		*p*	aOR	95% CI		*p*
Female	1.08	0.82	1.41	0.588	0.79	0.62	1.00	0.049	1.02	0.77	1.35	0.906
Age ≤34												
Age: 35-64	0.70	0.49	0.99	0.047	1.11	0.78	1.56	0.566	**0.56**	**0.38**	**0.82**	**0.003**
Age: 65+	**0.53**	**0.32**	**0.87**	**0.012**	1.10	0.71	1.70	0.676	0.99	0.60	1.61	0.961
White ethnicity	1.61	1.03	2.52	0.036	1.57	0.99	2.49	0.054	0.72	0.47	1.10	0.130
Household income ≥50.000												
Income: <50 000 GBP	**0.65**	**0.46**	**0.91**	**0.011**	0.71	0.52	0.96	0.028	1.13	0.76	1.67	0.556
Income: Prefer not to say	**0.44**	**0.25**	**0.77**	**0.004**	0.63	0.38	1.03	0.065	0.88	0.49	1.59	0.666
Education: Highschool or higher	**1.57**	**1.14**	**2.17**	**0.006**	1.04	0.81	1.35	0.745	**1.74**	**1.24**	**2.45**	**0.002**
Employed	0.76	0.55	1.05	0.094	1.09	0.82	1.43	0.562	1.06	0.75	1.49	0.734
Laid-off/Furloughed	0.79	0.51	1.23	0.295	1.05	0.71	1.54	0.813	1.14	0.71	1.83	0.584
Condition limiting physical activity	**0.44**	**0.28**	**0.71**	**0.001**	**0.43**	**0.29**	**0.63**	**<0.001**	0.97	0.65	1.45	0.881
BMI ≤24												
BMI: 25-29.99	0.81	0.60	1.10	0.176	1.01	0.78	1.32	0.926	0.72	0.53	0.99	0.043
BMI: Obese: 30+	**0.49**	**0.33**	**0.73**	**<0.001**	0.87	0.64	1.19	0.394	**0.37**	**0.24**	**0.56**	**<0.001**
Total isolation	**0.44**	**0.23**	**0.83**	**0.011**	**0.26**	**0.14**	**0.50**	**<0.001**	0.83	0.49	1.40	0.478
Minor/no Covid-19 risk percept	1.00	0.74	1.34	0.977	1.20	0.93	1.55	0.167	0.94	0.68	1.30	0.718
Living with children	0.81	0.57	1.16	0.254	0.75	0.55	1.03	0.079	0.66	0.44	0.99	0.045
Living with vulnerable	1.17	0.82	1.68	0.395	0.86	0.61	1.20	0.366	0.87	0.58	1.30	0.499
Access garden/balcony to exercise comfortably in	1.10	0.81	1.51	0.533	1.02	0.79	1.33	0.861	0.80	0.58	1.09	0.159
Access green space within walking distance	1.27	0.96	1.69	0.096	1.31	1.03	1.66	0.028	0.91	0.68	1.23	0.548
Smoker	0.72	0.51	1.02	0.062	0.93	0.70	1.22	0.593	0.80	0.57	1.14	0.218
Weekly alcohol drinking, none (ref)												
Weekly or less	1.00	0.68	1.49	0.988	1.43	1.02	2.00	0.038	1.06	0.71	1.59	0.782
More than weekly	1.04	0.73	1.50	0.811	1.28	0.94	1.74	0.121	0.89	0.61	1.29	0.528
Deteriorated psychological wellbeing	**0.56**	**0.43**	**0.73**	**<0.001**	**0.71**	**0.56**	**0.89**	**0.003**	0.72	0.55	0.96	0.025
Meeting WHO PA[a] rec pre-Covid (meeting neither=ref)												
Meeting both before	**16.11**	**11.24**	**23.07**	**<0.001**	**2.31**	**1.58**	**3.39**	**<0.001**	**3.72**	**2.45**	**5.62**	**<0.001**
Meeting only MVPA before	**3.88**	**2.64**	**5.70**	**<0.001**	**7.57**	**5.82**	**9.84**	**<0.001**	1.47	0.94	2.30	0.092
Meeting only MSA before	**6.38**	**4.26**	**9.54**	**<0.001**	0.74	0.44	1.25	0.264	**9.61**	**6.70**	**13.77**	**<0.001**

a=meeting World Health Physical activity recommendations before the first Covid-19 lockdown in the UK. Notes: All models were also adjusted for the time of enrolment and time when Covid-19 started to affect individuals in any way. Following the BH correction the highest p-value that met the threshold for significance was p=0.019

For peer review only

Table 4. Independent associations of change in MVPA (models fully adjusted using all the variables listed). Segmented analyses of active sample (pre-Covid-19 MVPA activity ≥30min; predicting decrease by ≥20min) and inactive sample (pre-Covid-19 MVPA activity <30min; predicting increase by ≥20min).

	Sample active (≥30min/week) pre-Covid-19 (weighted n=1857)				Sample inactive (<30min/week) pre-Covid-19 (weighted n=585)			
	Decrease (n=868) vs not				Increase (n=174) vs not			
	aOR	95% CI		*p*	aOR	95% CI		*p*
Female	1.22	1.00	1.49	0.047	1.56	1.02	2.38	0.040
Age (in decades)	**0.91**	**0.85**	**0.98**	**0.015**	**0.72**	**0.61**	**0.84**	**<0.001**
White ethnicity	**0.62**	**0.44**	**0.86**	**0.005**	1.01	0.44	2.33	0.982
Household income ≥50.000								
Income: <50 000 GBP	1.30	1.00	1.67	0.048	0.49	0.26	0.93	0.030
Income: Prefer not to say	1.22	0.81	1.85	0.335	0.36	0.14	0.93	0.034
Education: Highschool or higher	**0.73**	**0.58**	**0.91**	**0.006**	**2.26**	**1.40**	**3.63**	**0.001**
Employed	0.86	0.69	1.07	0.180	**0.51**	**0.31**	**0.85**	**0.009**
Laid-off/Furloughed	1.05	0.76	1.47	0.750	1.30	0.72	2.37	0.385
Condition limiting PA	**1.74**	**1.27**	**2.39**	**0.001**	0.49	0.27	0.89	0.019
BMI ≤24								
BMI: 25-29.99	1.03	0.82	1.28	0.816	1.54	0.94	2.53	0.088
BMI: Obese: 30+	0.87	0.66	1.14	0.317	0.74	0.42	1.31	0.307
Total isolation	**3.81**	**2.33**	**6.23**	**<0.001**	**0.29**	**0.12**	**0.73**	**0.008**
Minor/no Covid-19 risk percept	0.99	0.80	1.23	0.926	1.20	0.76	1.92	0.436
Living with children	0.84	0.65	1.09	0.187	**2.32**	**1.27**	**4.24**	**0.006**
Living with vulnerable	0.78	0.59	1.02	0.074	0.90	0.51	1.59	0.716
Access garden/balcony to exercise comfortably in	**0.75**	**0.60**	**0.94**	**0.011**	0.72	0.46	1.13	0.157
Access green space within walking distance	0.82	0.67	1.01	0.059	1.10	0.72	1.67	0.665
Smoker	1.09	0.85	1.40	0.504	1.15	0.73	1.80	0.551
Weekly alcohol drinking, none (ref)								
Weekly or less	0.78	0.59	1.04	0.087	0.77	0.43	1.38	0.378
More than weekly	0.78	0.60	1.02	0.068	0.88	0.52	1.50	0.644
Deteriorated psychological wellbeing	**1.40**	**1.15**	**1.71**	**0.001**	1.09	0.72	1.66	0.682

Notes: All models were also adjusted for the time of enrolment and time when Covid-19 started to affect individuals in any way. Following the BH correction the highest p-value that met the threshold for significance was p=0.019.

For peer review only

Table 5. Independent associations of change in MSA (models fully adjusted using all the variables listed). Segmented analyses of active sample (pre-Covid-19 MSA activity ≥1 day/week; predicting decrease by ≥1 day/week) and less inactive sample (pre-Covid-19 MSA activity 0 days/week; predicting increase by ≥1 day/week). Findings from fully adjusted logistic regression models on weighted data and using BH FDR adjustment (significant in bold).

	Sample active (≥1 day/week) pre-Covid-19 (weighted n=985)				Sample inactive (0 days/week) pre-Covid-19 (weighted n=1456)			
	Decrease (n=444) vs not				Increase (n=239) vs not			
	aOR	95% CI		*p*	aOR	95% CI		*p*
Female	0.94	0.71	1.23	0.634	0.96	0.70	1.31	0.792
Age (in decades)	1.03	0.94	1.14	0.516	-	-	-	-
Age ≤34								
Age: 35-64	-	-	-	-	**0.22**	**0.15**	**0.33**	**<0.001**
Age: 65+	-	-	-	-	**0.34**	**0.20**	**0.58**	**<0.001**
White ethnicity	0.75	0.51	1.11	0.154	0.71	0.41	1.23	0.216
Household income ≥50.000								
Income: <50 000 GBP	1.11	0.80	1.55	0.535	0.67	0.45	0.99	0.047
Income: Prefer not to say	0.94	0.54	1.62	0.818	0.56	0.29	1.08	0.084
Education: Highschool or higher	0.82	0.59	1.15	0.260	1.20	0.85	1.70	0.310
Employed	**1.81**	**1.34**	**2.46**	**<0.001**	1.08	0.75	1.55	0.671
Laid-off/Furloughed	1.34	0.85	2.12	0.205	1.30	0.80	2.10	0.289
Condition limiting PA	1.11	0.72	1.72	0.632	0.64	0.38	1.05	0.078
BMI ≤24.99								
BMI: 25-29.99	**1.88**	**1.39**	**2.55**	**<0.001**	0.89	0.63	1.26	0.512
BMI: Obese: 30+	**3.38**	**2.23**	**5.14**	**<0.001**	0.64	0.42	0.97	0.037
Total isolation	1.87	1.02	3.42	0.042	0.80	0.42	1.52	0.499
Minor/no Covid-19 risk percept	1.43	1.06	1.92	0.019	0.93	0.65	1.31	0.669
Living with children	1.01	0.71	1.44	0.964	1.48	0.94	2.31	0.088
Living with vulnerable	1.17	0.79	1.74	0.437	0.95	0.63	1.44	0.809
Access garden/balcony to exercise comfortably in	1.02	0.74	1.41	0.891	0.84	0.60	1.17	0.301
Access green space within walking distance	1.07	0.79	1.43	0.667	0.87	0.64	1.18	0.370
Smoker	0.63	0.43	0.91	0.013	1.04	0.74	1.46	0.828
Weekly alcohol drinking, none (ref)								
Weekly or less	1.11	0.74	1.65	0.620	1.13	0.73	1.76	0.580
More than weekly	1.26	0.86	1.85	0.237	1.06	0.71	1.59	0.786
Deteriorated psychological wellbeing	1.27	0.97	1.66	0.082	**0.62**	**0.46**	**0.83**	**0.002**

Notes: All models were also adjusted for the time of enrolment and time when Covid-19 started to affect individuals in any way. Following the BH correction the highest p-value that met the threshold for significance for was p=0.019.

For peer review only

Supplementary Materials 1-5

Manuscript Title: Correlates of and changes in aerobic physical activity and strength training before and after the onset of COVID-19 pandemic in the UK – findings from the HEBECO study.

Authors: Aleksandra Herbec[1,2*], Verena Schneider[1], Abi Fisher[1], Dimitra Kale[1], Lion Shahab[1], Phillippa Lally[1].

1 Department of Behavioural Science and Health, University College London, UK

2 Clinical, Educational and Health Psychology, University College London, UK

*contact: Aleksandra Herbec a.herbec@ucl.ac.uk

For peer review only

Abstract

Objectives: Understanding changes in moderate to vigorous aerobic physical activity (MVPA) and strength training (MSA) from before to after (pre-/post-) the onset of the Covid-19 pandemic in the UK and their correlates can inform interventions.

Methods: We analysed retrospective and concurrent data on MVPA/MSA pre- and post-Covid-19 (up until 14th June 2020) among 2,657 UK adults. The associations between socio-demographic and health characteristics, MVPA/MSA pre-Covid-19, living and exercise conditions and meeting WHO recommended levels for MVPA/MSA/both (vs meeting neither), and changes in MVPA/MSA from pre- to post-Covid-19 were evaluated following stratification for pre-Covid-19 MVPA and MSA levels.

Results: A third of adults maintained (30.4%), decreased (36.2%) or increased (33.4%) their MVPA levels post-Covid-19. For MSA, the percentages were 61.6%, 18.2%, and 20.2%, respectively. MVPA increased or decreased by an average of 150min/week, and MSA by 2 days/week. Meeting both MSA+MVPA recommendations during lockdown (vs. meeting neither) was positively associated with meeting MVPA+MSA pre-lockdown (aOR=16.11,95%CI=11.24-23.07), and post-16-years of age education (aOR=1.57,1.14-2.17), and negatively associated with being obese (aOR=0.49,0.33-0.73), older age (65+ vs ≤34; aOR=.53,.32-.87), and annual household income <50.000GBP (vs ≥50.000GBP; aOR=0.65,0.46-0.91). The odds for decreasing MVPA were significantly lower for white ethnicity, post-16-years of age education, access to garden/balcony, and higher for those who were in total isolation. The odds for decreasing MSA were significantly higher for those who were overweight or obese.

Conclusions: Aerobic and strength training were differently impacted during the first UK lockdown, with poorer outcomes for older participants, those in isolation, and those with higher BMI.

Keywords: aerobic activity, strength training, covid-19, UK, cross-sectional, changes, MSA, MVPA

Pre-registration: https://osf.io/spdtb

Supplementary Materials – HEBECO PA1 paper

Supplementary Materials 1: Changes to the pre-registered protocol....................2
Supplementary Materials 2: detailed description of study measures....................3
Supplementary materials 3: Univariable analyses....................5
Supplementary Materials 4: Unweighted analyses....................8
Supplementary Materials 5: Sensitivity Analyses for RQ3 (different cut-off values for MVPA change). 13

Supplementary Materials 1: Changes to the pre-registered protocol

The analysis protocol pre-registered on OSF prior to data inspection outlined analyses for research questions 3 and 4 involving unstratified multinomial analyses with change in MVPA and MSA as dependent variables. Specifically, the pre-registered protocol specified that we will use multinomial regression models to assess the correlates of a three-level dependent variables on change in (i) MVPA (increase by at least 20min/week, maintenance with change between 0 to 19min, and a decrease in MVPA by at least 20min/week) and in (ii) MSA (increase by at least 1 day/week, maintenance with change=0 days/week, and a decrease with change by at least 1 day/week). All the analyses were performed as per the original plan and can be accessed here: https://osf.io/2ujxq/.

Upon inspecting the data and the results a decision was made that this approach is not satisfactory as the group of maintainers of MVPA and MSA category included both inactive and active participants. This has motivated us to conduct and report the present analyses that stratified the sample based on pre-Covid-19 activity levels to identify meaningful changes.

For peer review only

Supplementary Materials 2: detailed description of study measures

Below are the details of the all the explanatory measures assessed at baseline that are relevant for the study. For the full wording of the baseline survey see: https://osf.io/bja7g/

Socio-demographic, health and wellbeing characteristics

Gender (female gender/other; prefer not to say (PNS) were treated as missing) was assessed by asking participants: “Which of the following best describes how you think of yourself?”.

Ethnicity (white/non-white; PNS treated as missing) was assessed by asking participants: “What is your ethnic group?”.

Education (high school education or higher; yes/no) was assessed by asking participants: “What is the highest level of education that you have completed?”

Employment (employed or self-employed; yes/no) and furloughed/laid off (yes/no) were assessed by asking participants: “What is your current MAIN occupation (during COVID-19)?”.

Income (low-middle <50 000 GBP vs high ≥50 000 GBP vs PNS) was assessed by asking participants: “What was your household annual income BEFORE COVID-19? (do not include income of friends)”.

Age was assessed as a continuous variable. For the analyses we used age in decades and entered it as a continuous variable. Where the continuous variable violated the linearity assumptions of the logistic regression models (as did its logarithmic and square root transformation), a 3-level categorical variable was created (≤34, 35-64, 65+).

Health behaviours and conditions

- Body mass index, BMI (normal and underweight ≤24.99/overweight 25-29.99/ obese ≥30/ unknown (included answers ‘don’t know’ and PNS to height or weight variables)). BMI was calculated by dividing participants weight in kilogram by their squared height in meters and categorised into: normal weight and underweight. BMI is associated with both MVPA and MSA levels (Churilla et al., 2018).

- Self-reports of any condition that limited their ability to engage in physical activity (yes/no; PNS treated as missing). Participants were asked: “Do you now suffer from any condition that limits you from engaging in physical activity, including walking or doing housework?”

- Self-perceived risk of Covid-19 to one’s health (minor or no risk/major, significant, moderate risk or ‘don’t know’) was assessed with the question: “What risk does COVID-19 pose to your health?”

- Smoking status (current smokers vs not) was assessed using a single item: “Which statement about tobacco use and cigarette smoking best describes you?”. Those responding 1 (“I smoke cigarettes (including hand-rolled) every day”) and 2 (“I smoke cigarettes (including hand-rolled), but not every day”) were classified as current smokers. Smoking is associated with physical activity levels (Kaczynski, Manske, Mannell, & Grewal, 2008).

For peer review only

- Weekly alcohol consumption since the Covid-19 pandemic has started was assessed by asking participants how many times they had a drink containing alcohol, with the categorical answers converted to weekly frequencies from 0 to 6.5 times per week (0=I have not had any alcohol after COVID-19 started, 0.25=monthly or less, 0.75=2 to 4 times a month, 2.5=2 to 3 times a week, 4.5=4 to 5 times a week, 6.5=6 or more times a week). In models where the continuous alcohol frequency variable violated the linearity assumptions of the logistic regression models (as did its logarithmic and square root transformation), weekly alcohol frequency was categorised into three levels (none/weekly or less often/more frequently than weekly). Alcohol drinking is associated with physical activity levels (Leasure, Neighbors, Henderson, & Young, 2015).

- Deterioration in psychological wellbeing (yes/no). Deterioration in psychological wellbeing was assessed on a five-point Likert scale (1=poor, 3=average, 5=excellent) by asking participants: "How would you rate the below aspects of your life? – Psychological wellbeing". The question was asked about the period before Covid-19 and since Covid-19. A change score was calculated by deducting the score before from since score. As the continuous variable violated the linearity assumptions of the logistic regression models (as did its logarithmic and square root transformation), participants were categorised into either deteriorated psychological wellbeing (change score < 0) versus maintained or improved psychological wellbeing (change score ≥ 0).

- Isolation status (total isolation/other) was assessed by asking which type of Covid-19 induced isolation participants were experiencing. Answers were dichotomised into indications of total isolation/quarantine ("not leaving the house for any reasons, not even to buy groceries or medications or to exercise") versus any other (some, general, or no isolation). Participants' perceived risk of Covid-19 to their health was dichotomised into 'no or minor risk perception' versus any other indication (moderate risk, significant, major or not known). It was hypothesised that perceived risk could affect physical activity, especially MVPA that may happen outdoors.

- Access to space to exercise was assessed using a single question on participants' access to spaces that allowed multiple answers, including for: „a garden or balcony big enough to exercise comfortably" and to "a public park/green space that is within a walking distance and open during Covid-19". The answers were dummy coded into separate binary variables (yes/no).

- Living with children age 15 and younger (yes/no) and living with vulnerable persons (any of: persons over the age of 70, persons in poor health versus, or persons who they believed may be vulnerable to Covid-19 for any reason) was assessed by asking participants: "Do you live with any of these persons below? (select all that apply)." It was hypothesised that living with these groups could limit some of the opportunities to exercise.

For peer review only

Supplementary materials 3: Univariable analyses.

Supplementary Table S3.1. (univariable) Research question 2 -Predictors of meeting both, MVPA only and MSA only in comparison to meeting WHO guidelines for neither MVPA nor MSA (reference, n=1178). Results from unadjusted models on weighted data with BH FDR correction of p-values (significant in bold).

	Both (n=388)				MVPA only (n=570)				MSA only (n=306)			
	OR	95% CI		*p*	OR	95% CI		*p*	OR	95% CI		*p*
Female	1.01	0.80	1.27	0.928	0.62	0.51	0.76	<0.001	0.93	0.72	1.19	0.546
Age ≤34[a]												
Age: 35-64	0.57	0.43	0.75	<0.001	1.08	0.82	1.42	0.604	0.41	0.30	0.56	<0.001
Age: 65+	0.41	0.28	0.60	<0.001	1.02	0.73	1.43	0.893	0.58	0.40	0.84	0.004
White ethnicity	0.77	0.52	1.12	0.166	1.53	1.02	2.30	0.040	0.43	0.30	0.62	<0.001
Household income ≥50.000												
Income: <50 000 GBP	0.44	0.33	0.59	<0.001	0.66	0.50	0.85	0.002	0.80	0.57	1.12	0.194
Income: Prefer not to say	0.41	0.25	0.66	<0.001	0.50	0.32	0.77	0.002	0.79	0.47	1.34	0.385
Education: Highschool or higher	2.57	1.96	3.38	<0.001	1.34	1.09	1.65	0.006	2.41	1.79	3.24	<0.001
Employed	1.14	0.90	1.43	0.272	1.20	0.98	1.46	0.077	1.05	0.81	1.35	0.714
Laid-off/Furloughed	0.95	0.67	1.35	0.784	1.01	0.75	1.36	0.972	1.04	0.72	1.51	0.839
Condition limiting PA	0.24	0.16	0.37	<0.001	0.27	0.19	0.37	<0.001	0.64	0.46	0.89	0.009
BMI ≤24												
BMI: 25-29.99	0.62	0.48	0.81	<0.001	0.86	0.68	1.08	0.191	0.60	0.45	0.79	<0.001
BMI: Obese: 30+	0.31	0.22	0.43	<0.001	0.62	0.48	0.81	<0.001	0.27	0.19	0.40	<0.001
Total isolation	0.30	0.17	0.52	<0.001	0.17	0.10	0.31	<0.001	0.67	0.43	1.05	0.079
Minor/no Covid-19 risk percept	1.70	1.34	2.16	<0.001	1.39	1.12	1.72	0.002	1.37	1.05	1.79	0.019
Living with children	0.99	0.73	1.33	0.925	0.81	0.61	1.06	0.121	0.70	0.49	1.01	0.056
Living with vulnerable	1.09	0.80	1.48	0.583	0.80	0.59	1.06	0.122	0.87	0.61	1.25	0.450
Access garden/balcony to exercise comfortably in	1.18	0.91	1.54	0.212	1.01	0.81	1.27	0.918	0.82	0.63	1.08	0.157
Access green space within walking distance	1.70	1.33	2.16	<0.001	1.59	1.29	1.95	<0.001	1.29	1.00	1.67	0.049
Smoker	0.48	0.35	0.64	<0.001	0.72	0.57	0.91	0.006	0.64	0.47	0.87	0.004
Alcohol drinking frequency, never (ref)												
Weekly or less	1.32	0.94	1.85	0.106	1.50	1.12	2.02	0.006	1.48	1.04	2.11	0.032
More than weekly	1.47	1.09	1.98	0.012	1.50	1.15	1.95	0.003	1.21	0.87	1.68	0.249
Deteriorated psychological wellbeing	0.62	0.50	0.79	<0.001	0.71	0.58	0.87	0.001	0.74	0.58	0.95	0.019
Meeting WHO PA rec (meeting neither=ref)												
Meeting both before	17.58	12.53	24.65	<0.001	2.30	1.60	3.31	<0.001	4.03	2.72	5.99	<0.001
Meeting only MVPA before	4.53	3.13	6.55	<0.001	8.30	6.47	10.65	<0.001	1.67	1.08	2.58	0.021
Meeting only MSA before	7.72	5.27	11.32	<0.001	0.86	0.52	1.42	0.549	10.31	7.37	14.43	<0.001

For peer review only

Supplementary Table S3.2. (univariable) Research question 3: Independent associations of change in MVPA. Segmented analyses of active sample (pre-Covid-19 MVPA activity ≥30min; predicting decrease by ≥20min) and less active/inactive sample (pre-Covid-19 MVPA activity <30min; predicting increase by ≥20min). Findings from unadjusted logistic regression models on weighted data and using BH FDR adjustment (significant in bold).

	Sample active (≥30min/week) pre-Covid-19 (weighted n=1857)				Sample inactive (<30min/week) pre-Covid-19 (weighted n=585)			
	Decrease (n=868) vs not				Increase (n=174) vs not			
	OR	95% CI		*p*	OR	95% CI		*p*
Female	1.38	1.15	1.65	0.001	1.13	0.79	1.62	0.496
Age (in decades)	0.94	0.88	0.99	0.024	0.67	0.59	0.75	<0.001
White ethnicity	0.57	0.42	0.77	<0.001	0.46	0.24	0.88	0.019
Household income ≥50.000								
Income: <50 000 GBP	1.36	1.08	1.71	0.010	0.45	0.27	0.77	0.003
Income: Prefer not to say	1.59	1.09	2.33	0.017	0.53	0.25	1.14	0.103
Education: Highschool or higher	0.72	0.59	0.88	0.001	2.67	1.81	3.93	<0.001
Employed	0.82	0.69	0.99	0.036	0.80	0.55	1.16	0.235
Laid-off/Furloughed	1.22	0.92	1.62	0.165	2.16	1.36	3.43	0.001
Condition limiting PA	2.28	1.71	3.04	<0.001	0.27	0.16	0.43	<0.001
BMI ≤24								
BMI: 25-29.99	1.01	0.82	1.23	0.961	1.04	0.69	1.55	0.863
BMI: Obese: 30+	0.99	0.78	1.27	0.945	0.56	0.34	0.90	0.016
Total isolation	4.95	3.11	7.88	<0.001	0.22	0.10	0.49	<0.001
Minor/no Covid-19 risk percept	0.94	0.77	1.14	0.503	2.17	1.50	3.14	<0.001
Living with children	0.89	0.70	1.13	0.319	1.37	0.82	2.30	0.225
Living with vulnerable	0.72	0.56	0.93	0.012	1.14	0.70	1.85	0.599
Access garden/balcony to exercise comfortably in	0.82	0.67	1.00	0.053	0.61	0.42	0.89	0.011
Access green space within walking distance	0.81	0.67	0.98	0.026	1.66	1.16	2.37	0.006
Smoker	0.94	0.75	1.17	0.576	1.15	0.79	1.68	0.454
Alcohol drinking frequency, never (ref)								
Weekly or less	0.68	0.52	0.89	0.005	1.19	0.73	1.96	0.483
More than weekly	0.60	0.47	0.76	<0.001	1.22	0.78	1.90	0.387
Deteriorated psychological wellbeing	1.42	1.18	1.71	<0.001	1.03	0.73	1.47	0.854

Adjusted for the time of enrolment and time when Covid-19 started to affect individuals in any way.

For peer review only

Supplementary Table S3.3 Research question 4: Independent associations of change in MSA. Segmented analyses of active sample (pre-Covid-19 MSA activity ≥1 day/week; predicting decrease by ≥1 day/week) and less inactive sample (pre-Covid-19 MSA activity 0 days/week; predicting increase by ≥1 day/week). Findings from unadjusted logistic regression models on weighted data and using BH FDR adjustment (significant in bold).

	Sample active (≥1 day/week) pre-Covid-19 (weighted n=985)				Sample inactive (0 days/week) pre-Covid-19 (weighted n=1456)			
	Decrease (n=444) vs not				Increase (n=239) vs not			
	OR	95% CI		*p*	OR	95% CI		*p*
Female	0.87	0.67	1.12	0.274	0.85	0.64	1.12	0.249
Age (in decades)	1.02	0.94	1.10	0.625	-	-	-	-
Age ≤34[a]								
Age: 35-64	-	-	-	-	0.20	0.14	0.28	<0.001
Age: 65+	-	-	-	-	0.27	0.18	0.40	<0.001
White ethnicity	0.80	0.57	1.13	0.210	0.36	0.22	0.58	<0.001
Household income ≥50.000								
Income: <50 000 GBP	1.01	0.75	1.36	0.953	0.61	0.42	0.87	0.007
Income: Prefer not to say	0.80	0.48	1.31	0.368	0.64	0.36	1.17	0.147
Education: Highschool or higher	0.74	0.55	1.00	0.051	1.86	1.37	2.52	<0.001
Employed	1.60	1.24	2.06	<0.001	1.01	0.76	1.33	0.951
Laid-off/Furloughed	0.99	0.67	1.45	0.954	1.33	0.91	1.96	0.141
Condition limiting PA	1.35	0.92	1.97	0.120	0.43	0.27	0.67	<0.001
BMI ≤24								
BMI: 25-29.99	1.88	1.42	2.50	<0.001	0.68	0.50	0.93	0.016
BMI: Obese: 30+	3.20	2.18	4.72	<0.001	0.43	0.29	0.63	<0.001
Total isolation	2.05	1.22	3.45	0.007	0.62	0.35	1.10	0.102
Minor/no Covid-19 risk percept	1.07	0.83	1.38	0.596	1.61	1.20	2.15	0.001
Living with children	0.83	0.60	1.15	0.266	1.44	0.95	2.18	0.086
Living with vulnerable	1.13	0.79	1.63	0.498	1.01	0.69	1.47	0.971
Access garden/balcony to exercise comfortably in	0.90	0.67	1.19	0.453	0.74	0.55	0.99	0.043
Access green space within walking distance	0.94	0.72	1.22	0.621	1.12	0.85	1.49	0.420
Smoker	0.65	0.46	0.90	0.010	1.00	0.74	1.36	0.995
Alcohol drinking frequency, never (ref)								
Weekly or less	0.88	0.61	1.27	0.508	1.50	1.00	2.27	0.051
More than weekly	0.93	0.67	1.30	0.677	1.40	0.97	2.03	0.072
Deteriorated psychological wellbeing	1.37	1.06	1.77	0.014	0.72	0.55	0.95	0.021

Adjusted for the time of enrolment and time when Covid-19 started to affect individuals in any way.

[a]Age was categorised in models where the continuous predictor violated the linearity assumption.

For peer review only

Supplementary Materials 4: Unweighted analyses

Supplementary Table S4.1. (unweighted data) Sample characteristics % (N).

	Included sample (2657)	Excluded sample (336)	*p*
Female	68.9 (1831)	68.1 (224)	0.760
Age; M (SD)	48.4 (15.2)	43.7 (16.7)	<0.001
White ethnicity	94.4 (2507)	91.1 (297)	0.020
Household income ≥50.000	90.9 (993)	9.1 (100)	<0.001
Income: <50 000 GBP	54.5 (1447)	55.5 (186)	
Income: Prefer not to say	8.2 (217)	14.6 (49)	
Education: Highschool or higher	86.8 (2307)	85.7 (288)	0.571
Employed	56.1 (1490)	49.1 (164)	0.016
Laid-off/Furloughed	9.8 (261)	12.3 (41)	0.161
Condition limiting PA	13.2 (351)	19.6 (54)	0.003
BMI ≤24	49.1 (1305)	23.8 (62)	<0.001
BMI: 25-29.99	31.3 (832)	15.8 (41)	
BMI: Obese: 30+	19.6 (520)	9.2 (24)	
Total isolation	7.2 (190)	7.2 (21)	0.955
Minor/no Covid-19 risk percept	37.5 (996)	42.4 (123)	0.101
Living with children	19.6 (520)	19.0 (64)	0.820
Living with vulnerable	14.8 (394)	16.1 (54)	0.547
Access garden/balcony	71.9 (1911)	63.1 (212)	0.001
Access green space	63.5 (1687)	57.1 (192)	0.023
Time affected up till mid March	49.5 (1316)	55.1 (185)	0.056
Enrolled from 1st June (ref)	5.2 (139)	4.8 (16)	0.479
Enrolled up until 15th May	48.9 (1298)	45.8 (154)	
Enrolled second half of May	45.9 (1220)	49.4 (166)	
Smoker	17.7 (470)	25.6 (86)	<0.001
Alcohol drinking frequency, never (ref)	17.0 (453)	19.6 (66)	0.092
Weekly or less	27.5 (732)	31.3 (105)	
More than weekly	55.4 (1472)	49.1 (165)	
Deteriorated psychological wellbeing	55.1 (1465)	50.2 (117)	0.148
Meeting WHO PA rec (meeting both=ref)	17.9 (475)	14.9 (26)	0.332
Meeting only MVPA before	22.8 (605)	23.0 (40)	
Meeting only MSA before	14.9 (395)	11.5 (20)	
Meeting neither before	44.5 (1182)	50.6 (88)	

For peer review only

Supplementary Table S4.2. (unweighted data) Aerobic physical activity (MVPA) and strength training (MSA) before to after the Covid-19 pandemic has started to affect individuals in the UK (up until 14th June 2020).

	Before Covid-19[1]	Since Covid-19[1]
Inactive (0 sessions MVPA and 0 days MSA) % (N)	14.1 (375)	16.0 (425)
MVPA 0 sessions % (N)	15.9 (422)	18.5 (491)
MVPA		
MVPA min/week among active; Median (IQR)	135.0 (180.0)	150.0 (240.0)
Mean (SD)	215.3 (363.4)	227.0 (341.7)
Mean (SD) replacing extremes with 840 min/week	191.8 (186.9)	206.9 (193.7)
MVPA min/week among entire sample; Median (IQR)	120.0 (185.0)	120.0 (215.0)
Mean (SD)	184.9 (345.0)	190.7 (324.1)
Mean (SD) replacing extremes with 840 min/week	164.7 (185.6)	173.8 (193.1)
MSA Median (IQR)	0 (2)	0 (2)
MSA % (N)		
0 das/week	53.0 (1408)	54.0 (1435)
1 day/week	14.3 (379)	11.7 (311)
2 days/week	14.6 (387)	10.5 (280)
3 days/week	10.4 (277)	9.6 (254)
4+ days/week	7.8 (206)	14.2 (377)
Meet guidelines % (N)		
Neither MVPA nor MSA	44.5 (1182)	42.0 (1117)
Meet MVPA (≥150min/week) only	22.8 (605)	23.7 (629)
Meet MSA (≥2 days/week) only	14.9 (395)	14.7 (390)
Meets both MVPA and MSA	17.9 (475)	19.6 (521)
Change in form of exercise % (N)		
None of the same		13.2 (350)
Some of the same		29.1 (774)
About half the same		8.8 (233)
Mostly the same		23.4 (622)
Exactly the same		10.0 (267)

For peer review only

Supplementary Table S4.3. (unweighted data) Research question 2 – Factors associated with meeting both, MVPA only and MSA only in comparison to meeting WHO guidelines for neither MVPA nor MSA (reference, n=1117) after the Covid-19 pandemic has started to affect individuals in the UK (up until June 2020[1]). Results from fully adjusted models on unweighted data with BH FDR correction of p-values (significant in bold).

	Both (n=521)				MVPA only (n=629)				MSA only (n=390)			
Characteristics and associations	aOR	95% CI		*p*	aOR	95% CI		*p*	aOR	95% CI		*p*
Female	1.17	0.89	1.52	0.256	0.93	0.73	1.18	0.546	1.16	0.87	1.53	0.308
Age ≤34[a]												
Age: 35-64	0.92	0.67	1.25	0.589	1.49	1.09	2.05	0.013	0.59	0.43	0.82	0.001
Age: 65+	0.64	0.40	1.03	0.067	1.80	1.17	2.75	0.007	0.90	0.57	1.44	0.664
White ethnicity	1.36	0.81	2.27	0.245	1.79	1.01	3.15	0.044	0.67	0.42	1.08	0.099
Household income ≥50.000												
Income: <50 000 GBP	0.58	0.44	0.76	<0.001	0.70	0.55	0.90	0.006	1.00	0.75	1.33	0.976
Income: Prefer not to say	0.55	0.34	0.89	0.014	0.76	0.49	1.17	0.214	0.90	0.54	1.48	0.667
Education: Highschool or higher	1.38	0.92	2.09	0.121	1.20	0.86	1.67	0.293	1.86	1.19	2.90	0.007
Employed	0.78	0.58	1.05	0.102	1.11	0.85	1.47	0.442	1.22	0.89	1.68	0.212
Laid-off/Furloughed	1.00	0.64	1.55	0.997	1.07	0.70	1.62	0.759	1.33	0.84	2.13	0.227
Condition limiting PA	0.42	0.27	0.65	<0.001	0.36	0.24	0.54	<0.001	0.78	0.53	1.16	0.219
BMI ≤24												
BMI: 25-29.99	0.61	0.46	0.80	<0.001	0.80	0.62	1.02	0.076	0.66	0.49	0.88	0.005
BMI: Obese: 30+	0.35	0.24	0.51	<0.001	0.69	0.51	0.93	0.014	0.43	0.30	0.62	<0.001
Total isolation	0.39	0.22	0.69	0.001	0.22	0.12	0.41	<0.001	0.84	0.52	1.38	0.498
Minor/no Covid-19 risk percept	0.82	0.64	1.07	0.147	0.92	0.72	1.17	0.476	0.90	0.68	1.20	0.477
Living with children	0.79	0.58	1.07	0.130	0.56	0.42	0.76	<0.001	0.89	0.64	1.24	0.497
Living with vulnerable	1.14	0.81	1.60	0.457	1.07	0.79	1.46	0.662	0.94	0.65	1.35	0.721
Access garden/balcony to exercise comfortably in	1.09	0.82	1.44	0.562	1.00	0.78	1.29	0.994	0.98	0.74	1.31	0.901
Access green space within walking distance	1.17	0.90	1.51	0.243	1.23	0.97	1.56	0.081	1.16	0.89	1.53	0.278
Smoker	0.56	0.40	0.79	0.001	0.69	0.52	0.93	0.016	0.65	0.46	0.92	0.016
Alcohol drinking frequency, never (ref)												
Weekly or less	1.24	0.84	1.82	0.279	1.16	0.82	1.63	0.405	1.07	0.72	1.58	0.745
More than weekly	1.25	0.88	1.78	0.221	1.14	0.83	1.56	0.412	1.02	0.71	1.47	0.923
Deteriorated psychological wellbeing	0.61	0.48	0.78	<0.001	0.78	0.63	0.98	0.029	0.65	0.51	0.84	0.001
Meeting WHO PA rec (meeting neither=ref)												
Meeting both before	14.58	10.48	20.30	<0.001	2.47	1.74	3.52	<0.001	3.53	2.44	5.10	<0.001
Meeting only MVPA before	3.31	2.35	4.66	<0.001	5.86	4.54	7.55	<0.001	0.84	0.54	1.31	0.445
Meeting only MSA before	4.98	3.49	7.11	<0.001	0.79	0.51	1.24	0.303	6.59	4.80	9.06	<0.001

Models adjusted for the time of enrolment and time when Covid-19 started to affect individuals in any way.

[1] Covering the period of the strictest first lockdown in the UK

For peer review only

Supplementary Table S4.4. Research question 3: Independent associations of change in MVPA from before to after the Covid-19 pandemic has started to affect individuals in the UK (up until 14th June 2020[2]). Segmented analyses of active sample (pre-Covid-19 MVPA activity ≥30min; predicting decrease by ≥20min) and less active/inactive sample (pre-Covid-19 MVPA activity <30min; predicting increase by ≥20min). Findings from fully adjusted logistic regression models on unweighted data and using BH FDR adjustment (significant in bold).

Characteristics and associations	Sample active (≥30min/week) pre-Covid-19 (unweighted n=2132)				Sample inactive (<30min/week) pre-Covid-19 (unweighted n=525)			
	Decrease (n=905) vs not				Increase (n=195) vs not			
	aOR	95% CI		*p*	aOR	95% CI		*p*
Female	1.05	0.86	1.27	0.660	1.12	0.73	1.74	0.605
Age (in decades)	0.91	0.85	0.98	0.011	0.80	0.68	0.94	0.007
White ethnicity	0.52	0.35	0.76	0.001	0.80	0.30	2.15	0.656
Household income ≥50.000								
Income: <50 000 GBP	1.41	1.15	1.73	0.001	0.95	0.59	1.53	0.829
Income: Prefer not to say	1.31	0.92	1.87	0.130	0.72	0.32	1.65	0.441
Education: Highschool or higher	0.73	0.54	0.98	0.036	1.87	1.07	3.26	0.028
Employed	0.96	0.77	1.18	0.682	0.70	0.43	1.13	0.140
Laid-off/Furloughed	0.95	0.68	1.34	0.782	1.51	0.77	2.93	0.227
Condition limiting PA	1.87	1.37	2.55	<0.001	0.47	0.27	0.83	0.010
BMI ≤24								
BMI: 25-29.99	1.08	0.88	1.33	0.456	1.11	0.69	1.79	0.665
BMI: Obese: 30+	0.97	0.75	1.26	0.833	0.71	0.42	1.19	0.195
Total isolation	2.91	1.89	4.46	<0.001	0.22	0.09	0.54	0.001
Minor/no Covid-19 risk percept	1.21	0.99	1.47	0.067	1.19	0.76	1.87	0.441
Living with children	0.80	0.64	1.01	0.061	0.86	0.52	1.44	0.570
Living with vulnerable	0.92	0.71	1.20	0.551	0.83	0.49	1.43	0.511
Access garden/balcony to exercise comfortably in	0.74	0.60	0.91	0.005	0.96	0.61	1.49	0.847
Access green space within walking distance	0.95	0.78	1.15	0.589	1.45	0.96	2.19	0.077
Smoker	0.88	0.68	1.13	0.311	1.70	1.05	2.74	0.030
Alcohol drinking frequency, never (ref)								
Weekly or less	1.08	0.81	1.44	0.605	0.96	0.54	1.71	0.890
More than weekly	0.89	0.68	1.16	0.378	1.24	0.75	2.06	0.399
Deteriorated psychological wellbeing	1.40	1.16	1.68	<0.001	1.12	0.74	1.67	0.597

Models adjusted for the time of enrolment and time when Covid-19 started to affect individuals in any way.

For peer review only

[2] Covering the period of the strictest first lockdown in the UK

Supplementary Table S4.5. (unweighted data). Research question 4: Independent associations of change in MSA from before to after the Covid-19 pandemic has started to affect individuals in the UK (up until 14th June 2020[3]). Segmented analyses of active sample (pre-Covid-19 MSA activity ≥1 day/week; predicting decrease by ≥1 day/week) and less inactive sample (pre-Covid-19 MSA activity 0 days/week; predicting increase by ≥1 day/week). Findings from fully adjusted logistic regression models on unweighted data and using BH FDR adjustment (significant in bold).

	Sample active (≥1 day/week) pre-Covid-19 (unweighted n=1249)				Sample inactive (0 days/week) pre-Covid-19 (unweighted n=1408)			
	Decrease (n=534) vs not				Increase (n=284) vs not			
Characteristics and associations	aOR	95% CI		*p*	aOR	95% CI		*p*
Female	0.83	0.64	1.08	0.160	1.216	0.899	1.647	0.205
Age (in decades)	1.03	0.94	1.12	0.579	-	-	-	-
Age ≤34[a]								
Age: 35-64	-	-	-	-	0.353	0.248	0.502	<0.001
Age: 65+	-	-	-	-	0.305	0.178	0.524	<0.001
White ethnicity	0.89	0.57	1.39	0.611	0.553	0.298	1.024	0.060
Household income ≥50.000								
Income: <50 000 GBP	1.25	0.96	1.64	0.096	0.678	0.496	0.928	0.015
Income: Prefer not to say	1.39	0.88	2.18	0.155	0.480	0.258	0.893	0.020
Education: Highschool or higher	0.96	0.62	1.49	0.863	1.310	0.837	2.051	0.237
Employed	1.49	1.12	1.97	0.005	0.900	0.634	1.278	0.557
Laid-off/Furloughed	1.18	0.75	1.85	0.467	1.104	0.676	1.803	0.694
Condition limiting PA	1.08	0.72	1.62	0.708	0.664	0.409	1.078	0.097
BMI ≤24								
BMI: 25-29.99	1.63	1.24	2.13	<0.001	0.846	0.613	1.168	0.310
BMI: Obese: 30+	2.50	1.73	3.60	<0.001	0.707	0.484	1.031	0.072
Total isolation	2.03	1.18	3.50	0.011	0.861	0.481	1.543	0.615
Minor/no Covid-19 risk percept	1.18	0.91	1.52	0.216	1.045	0.769	1.421	0.777
Living with children	1.04	0.77	1.41	0.784	0.708	0.503	0.997	0.048
Living with vulnerable	1.11	0.78	1.59	0.553	0.990	0.677	1.446	0.957
Access garden/balcony to exercise comfortably in	0.86	0.65	1.13	0.275	0.765	0.562	1.040	0.088
Access green space within walking distance	0.95	0.74	1.23	0.713	1.102	0.816	1.489	0.527
Smoker	0.61	0.43	0.87	0.007	1.096	0.772	1.557	0.608
Alcohol drinking frequency, never (ref)								
Weekly or less	1.01	0.69	1.49	0.943	1.192	0.776	1.833	0.422
More than weekly	0.99	0.69	1.42	0.951	1.124	0.755	1.672	0.565
Deteriorated psychological wellbeing	1.38	1.09	1.76	0.008	0.643	0.488	0.848	0.002

Models adjusted for the time of enrolment and time when Covid-19 started to affect individuals in any way.

[a]Age was categorised in models where the continuous predictor violated the linearity assumption.

For peer review only

[3] Covering the period of the strictest first lockdown in the UK

For peer review only - http://bmjopen.bmj.com/site/about/guidelines.xhtml

Supplementary Materials 5: Sensitivity Analyses for RQ3 (different cut-off values for MVPA change).

Supplementary Table S5.1. Predicting MVPA decrease of at least 15 min in the active sample from before to after the Covid-19 pandemic has started to affect individuals in the UK (up until 14th June 2020). (fully adjusted model, weighted data).

Characteristics and associations	aOR	95% CI		***p***
Female	1.232	1.010	1.503	.039
Age (in decades)	.914	.849	.983	.015
White ethnicity	.649	.464	.909	.012
Household income ≥50.000	1.0			
Income: <50 000 GBP	1.272	.986	1.641	.064
Income: Prefer not to say	1.182	.783	1.783	.427
Education: Highschool or higher	.754	.602	.944	.014
Employed	.841	.677	1.045	.118
Laid-off/Furloughed	1.025	.738	1.423	.884
Condition limiting PA	1.732	1.260	2.381	.001
BMI ≤24	1.0			
BMI: 25-29.99	1.025	.822	1.280	.824
BMI: Obese: 30+	.875	.667	1.147	.334
Total isolation	3.682	2.252	6.020	.000
Minor/no Covid-19 risk percept	.984	.791	1.224	.882
Living with children	.858	.661	1.114	.251
Living with vulnerable	.801	.607	1.058	.118
Access garden/balcony to exercise comfortably in	.773	.618	.968	.025
Access green space within walking distance	.813	.662	.998	.048
Smoker	1.106	.861	1.420	.432
Alcohol drinking frequency, never (ref)	1.0			
Weekly or less	.790	.596	1.049	.103
More than weekly	.772	.594	1.003	.053
Deteriorated psychological wellbeing	1.422	1.170	1.728	.000

Model was adjusted for all the variable sin the table as well as the time of enrolment and time when Covid-19 started to affect individuals in any way. Significant results are in bold.

Supplementary Table S5.2. Predicting MVPA increase of at least 15 min in the active sample from before to after the Covid-19 pandemic has started to affect individuals in the UK (up until 14th June 2020; fully adjusted model, weighted data).

Characteristics and associations	aOR	95% CI		p
Female	1.513	1.003	2.282	.048
Age (in decades)	.750	.642	.876	.000
White ethnicity	.564	.249	1.276	.169
Household income ≥50.000	1.0			
Income: <50 000 GBP	.633	.339	1.182	.152
Income: Prefer not to say	.663	.271	1.620	.367
Education: Highschool or higher	1.700	1.078	2.683	.023
Employed	.846	.526	1.362	.492
Laid-off/Furloughed	1.380	.762	2.498	.288
Condition limiting PA	.445	.249	.795	.006
BMI ≤24	1.0			
BMI: 25-29.99	1.640	1.014	2.652	.044
BMI: Obese: 30+	.778	.451	1.343	.368
Total isolation	.395	.172	.907	.029
Minor/no Covid-19 risk percept	1.349	.857	2.122	.196
Living with children	1.600	.917	2.793	.098
Living with vulnerable	.872	.505	1.508	.624
Access garden/balcony to exercise comfortably in	.730	.469	1.136	.163
Access green space within walking distance	.986	.656	1.481	.944
Smoker	1.491	.963	2.309	.073
Alcohol drinking frequency, never (ref)	1.0			
Weekly or less	.719	.407	1.272	.257
More than weekly	.941	.567	1.563	.814
Deteriorated psychological wellbeing	1.275	.850	1.913	.241

Model was adjusted for all the variable sin the table as well as the time of enrolment and time when Covid-19 started to affect individuals in any way. Significant results are in bold.

For peer review only

Supplementary Table S5.3. Predicting MVPA decrease of at least 30 min in the active sample (fully adjusted model, weighted data).

Characteristics and associations	aOR	95% CI		p
Female	1.207	.989	1.474	.064
Age (in decades)	.904	.841	.973	.007
White ethnicity	.618	.441	.865	.005
Household income ≥50.000	1.0			
Income: <50 000 GBP	1.356	1.049	1.753	.020
Income: Prefer not to say	1.276	.845	1.927	.246
Education: Highschool or higher	.741	.592	.928	.009
Employed	.853	.686	1.061	.153
Laid-off/Furloughed	1.037	.747	1.441	.827
Condition limiting PA	1.672	1.219	2.293	.001
BMI ≤24	1.0			
BMI: 25-29.99	1.047	.838	1.308	.684
BMI: Obese: 30+	.880	.671	1.155	.357
Total isolation	3.426	2.130	5.511	.000
Minor/no Covid-19 risk percept	.978	.785	1.218	.842
Living with children	.837	.644	1.087	.182
Living with vulnerable	.756	.572	.998	.048
Access garden/balcony to exercise comfortably in	.744	.594	.932	.010
Access green space within walking distance	.820	.667	1.008	.059
Smoker	1.097	.854	1.410	.468
Alcohol drinking frequency, never (ref)	1.0			
Weekly or less	.753	.567	.999	.049
More than weekly	.752	.578	.977	.033
Deteriorated psychological wellbeing	1.403	1.154	1.706	.001

Model was adjusted for all the variable sin the table as well as the time of enrolment and time when Covid-19 started to affect individuals in any way. Significant results are in bold.

only

Supplementary Table S5.4. Predicting MVPA increase of at least 30 min in the inactive sample (fully adjusted model, weighted data).

Characteristics and associations	aOR	95% CI		p
Female	1.877	1.213	2.902	.005
Age (in decades)	.707	.599	.836	.000
White ethnicity	1.391	.575	3.363	.464
Household income ≥50.000	1.0			
Income: <50 000 GBP	.394	.205	.757	.005
Income: Prefer not to say	.208	.077	.560	.002
Education: Highschool or higher	2.116	1.296	3.456	.003
Employed	.516	.309	.861	.011
Laid-off/Furloughed	1.380	.757	2.517	.293
Condition limiting PA	.478	.257	.887	.019
BMI ≤24	1.0			
BMI: 25-29.99	1.511	.917	2.490	.105
BMI: Obese: 30+	.616	.341	1.113	.108
Total isolation	.332	.133	.827	.018
Minor/no Covid-19 risk percept	1.001	.620	1.616	.997
Living with children	2.290	1.225	4.283	.009
Living with vulnerable	.936	.519	1.687	.825
Access garden/balcony to exercise comfortably in	.764	.482	1.209	.250
Access green space within walking distance	.960	.622	1.481	.853
Smoker	.979	.622	1.542	.926
Alcohol drinking frequency, never (ref)	1.0			
Weekly or less	1.028	.555	1.904	.931
More than weekly	1.234	.708	2.152	.459
Deteriorated psychological wellbeing	.888	.579	1.362	.585

Model was adjusted for all the variable sin the table as well as the time of enrolment and time when Covid-19 started to affect individuals in any way. Significant results are in bold.

For peer review only

Reporting checklist for cross sectional study.

Based on the STROBE cross sectional guidelines.

Instructions to authors

Complete this checklist by entering the page numbers from your manuscript where readers will find each of the items listed below.

Your article may not currently address all the items on the checklist. Please modify your text to include the missing information. If you are certain that an item does not apply, please write "n/a" and provide a short explanation.

Upload your completed checklist as an extra file when you submit to a journal.

In your methods section, say that you used the STROBE cross sectionalreporting guidelines, and cite them as:

von Elm E, Altman DG, Egger M, Pocock SJ, Gotzsche PC, Vandenbroucke JP. The Strengthening the Reporting of Observational Studies in Epidemiology (STROBE) Statement: guidelines for reporting observational studies.

For peer review only

		Reporting Item	Page Number
**Title and abstract**			
Title	#1a	Indicate the study's design with a commonly used term in the title or the abstract	2
Abstract	#1b	Provide in the abstract an informative and balanced	2

		summary of what was done and what was found	
**Introduction**			
Background / rationale	#2	Explain the scientific background and rationale for the investigation being reported	4
Objectives	#3	State specific objectives, including any prespecified hypotheses	4
**Methods**			
Study design	#4	Present key elements of study design early in the paper	5-6
Setting	#5	Describe the setting, locations, and relevant dates, including periods of recruitment, exposure, follow-up, and data collection	5
Eligibility criteria	#6a	Give the eligibility criteria, and the sources and methods of selection of participants.	5
	#7	Clearly define all outcomes, exposures, predictors, potential confounders, and effect modifiers. Give diagnostic criteria, if applicable	5-6, Supplement 2
Data sources / measurement	#8	For each variable of interest give sources of data and details of methods of assessment (measurement). Describe comparability of assessment methods if there is more than one group. Give information separately for for exposed and unexposed groups if applicable.	5-6
Bias	#9	Describe any efforts to address potential sources of bias	7

For peer review only

Study size	#10	Explain how the study size was arrived at	5, 7
Quantitative variables	#11	Explain how quantitative variables were handled in the analyses. If applicable, describe which groupings were chosen, and why	5-7
Statistical methods	#12a	Describe all statistical methods, including those used to control for confounding	7
Statistical methods	#12b	Describe any methods used to examine subgroups and interactions	7
Statistical methods	#12c	Explain how missing data were addressed	5-7, Supplement 2
Statistical methods	#12d	If applicable, describe analytical methods taking account of sampling strategy	7
Statistical methods	#12e	Describe any sensitivity analyses	7
**Results**			
Participants	#13a	Report numbers of individuals at each stage of study—eg numbers potentially eligible, examined for eligibility, confirmed eligible, included in the study, completing follow-up, and analysed. Give information separately for for exposed and unexposed groups if applicable.	7
Participants	#13b	Give reasons for non-participation at each stage	7

For peer review only

Participants	#13c	Consider use of a flow diagram	N/A
Descriptive data	#14a	Give characteristics of study participants (eg demographic, clinical, social) and information on exposures and potential confounders. Give information separately for exposed and unexposed groups if applicable.	7, Table 1
Descriptive data	#14b	Indicate number of participants with missing data for each variable of interest	Table 1
Outcome data	#15	Report numbers of outcome events or summary measures. Give information separately for exposed and unexposed groups if applicable.	7-8 Tables
Main results	#16a	Give unadjusted estimates and, if applicable, confounder-adjusted estimates and their precision (eg, 95% confidence interval). Make clear which confounders were adjusted for and why they were included	7-8 Tables
Main results	#16b	Report category boundaries when continuous variables were categorized	5-8
Main results	#16c	If relevant, consider translating estimates of relative risk into absolute risk for a meaningful time period	N/A
Other analyses	#17	Report other analyses done—e.g., analyses of subgroups and interactions, and sensitivity analyses	7, Suplements

Discussion

Key results	#18	Summarise key results with reference to study objectives	8-10

For peer review only

Limitations	#19	Discuss limitations of the study, taking into account sources of potential bias or imprecision. Discuss both direction and magnitude of any potential bias.	10
Interpretation	#20	Give a cautious overall interpretation considering objectives, limitations, multiplicity of analyses, results from similar studies, and other relevant evidence.	10
Generalisability	#21	Discuss the generalisability (external validity) of the study results	10

Other Information

Funding	#22	Give the source of funding and the role of the funders for the present study and, if applicable, for the original study on which the present article is based	11

None The STROBE checklist is distributed under the terms of the Creative Commons Attribution License CC-BY. This checklist can be completed online using https://www.goodreports.org/, a tool made by the EQUATOR Network in collaboration with Penelope.ai

For peer review only

BMJ Open

Correlates of and changes in aerobic physical activity and strength training before and after the onset of COVID-19 pandemic in the UK – findings from the HEBECO study.

Journal:	*BMJ Open*
Manuscript ID	bmjopen-2021-054029.R1
Article Type:	Original research
Date Submitted by the Author:	16-Dec-2021
Complete List of Authors:	Herbec, Aleksandra; University College London,<br>Schneider, Verena; University College London<br>Fisher, Abigail; University College London<br>Kale, Dimitra; University College London<br>Shahab, Lion; University College London<br>Lally, Phillippa; University College London
**Primary Subject Heading**:	Public health
Secondary Subject Heading:	Epidemiology, Public health
Keywords:	COVID-19, SPORTS MEDICINE, PUBLIC HEALTH

SCHOLARONE™
Manuscripts

I, the Submitting Author has the right to grant and does grant on behalf of all authors of the Work (as defined in the below author licence), an exclusive licence and/or a non-exclusive licence for contributions from authors who are: i) UK Crown employees; ii) where BMJ has agreed a CC-BY licence shall apply, and/or iii) in accordance with the terms applicable for US Federal Government officers or employees acting as part of their official duties; on a worldwide, perpetual, irrevocable, royalty-free basis to BMJ Publishing Group Ltd ("BMJ") its licensees and where the relevant Journal is co-owned by BMJ to the co-owners of the Journal, to publish the Work in this journal and any other BMJ products and to exploit all rights, as set out in our licence.

The Submitting Author accepts and understands that any supply made under these terms is made by BMJ to the Submitting Author unless you are acting as an employee on behalf of your employer or a postgraduate student of an affiliated institution which is paying any applicable article publishing charge ("APC") for Open Access articles. Where the Submitting Author wishes to make the Work available on an Open Access basis (and intends to pay the relevant APC), the terms of reuse of such Open Access shall be governed by a Creative Commons licence – details of these licences and which Creative Commons licence will apply to this Work are set out in our licence referred to above.

Other than as permitted in any relevant BMJ Author's Self Archiving Policies, I confirm this Work has not been accepted for publication elsewhere, is not being considered for publication elsewhere and does not duplicate material already published. I confirm all authors consent to publication of this Work and authorise the granting of this licence.

For peer review only

Title: Correlates of and changes in aerobic physical activity and strength training before and after the onset of COVID-19 pandemic in the UK – findings from the HEBECO study.

Authors: Aleksandra Herbec[1,2*], Verena Schneider[1], Abi Fisher[1], Dimitra Kale[1], Lion Shahab[1], Phillippa Lally[1].

1 Department of Behavioural Science and Health, University College London, UK

2 Clinical, Educational and Health Psychology, University College London, UK

*contact: Aleksandra Herbec a.herbec@ucl.ac.uk

Pre-registration: https://osf.io/spdtb

Tables are at the end of the manuscript

For peer review only

Abstract

Objectives: Understanding changes in moderate to vigorous aerobic physical activity (MVPA) and muscle-strengthening activity (MSA) at the start of the COVID-19 pandemic and their correlates (socio-demographics, health characteristics, living and exercise conditions, and pre-pandemic MVPA/MSA) can inform interventions.

Design: Cross-sectional analysis of retrospective and concurrent data on MVPA/MSA.

Setting: Online survey in the United Kingdom.

Participants: 2,657 adults (weighted n=2442, 53.6% women) participating in the baseline survey (29th April – 14th June 2020) of the Health Behaviours during Covid-19 (HEBECO) study.

Primary and secondary outcome measures: Meeting World Health Organisation recommended levels for MVPA/MSA/both (vs meeting neither) during the first lockdown and changes in MVPA/MSA from before to since the Covid-19 pandemic following stratification for pre-pandemic MVPA/MSA.

Results: A third of adults maintained (30.4%), decreased (36.2%) or increased (33.4%) MVPA-. For MSA, the percentages were 61.6%, 18.2%, and 20.2%, respectively. MVPA increased or decreased by an average of 150min/week and 219min/week, respectively, and MSA by 2 days/week. Meeting both MSA+MVPA recommendations since Covid-19 (vs. meeting neither) was positively associated with meeting MVPA+MSA before Covid-19 (aOR=16.11,95%CI=11.24-23.07) and post-16 years of age education (aOR=1.57,1.14-2.17), and negatively associated with having obesity (aOR=0.49,0.33-0.73), older age (65+ vs ≤34; aOR=.53,.32-.87) and annual household income <50 000GBP (aOR=0.65,0.46-0.91). The odds for decreasing MVPA were lower for white ethnicity (aOR=0.62,0.44-.86), post-16 years of age education (aOR=0.73,0.58-0.91), access to garden/balcony (aOR=0.75,0.60-0.94), and were higher for those living in total isolation (aOR=3.81,2.33-6.23), with deteriorated psychological wellbeing (aOR=1.40,1.15-1.71) and conditions limiting physical activity (aOR=1.74,1,27-2.39). The odds for decreasing MSA were higher for having overweight (aOR=1.88,1.39-2.55), obesity (aOR=23.38,2.23-5.14) and being employed (aOR=1.81,1.34-2.46).

Conclusion: Aerobic and strength training were differently impacted during the first UK lockdown, with poorer outcomes associated with older age, lower education, and higher body mass index. Targeted interventions may be required to avoid pandemic-related inequities in physical activity.

Keywords: aerobic activity, strength training, Covid-19, UK, cross-sectional, changes, MSA, MVPA

For peer review only

Strengths and limitations of this study

- Novel data on the unique correlates of increases and decreases in WHO recommended levels of physical activity at the onset of the Covid-19 pandemic.
- Two types of physical activity, moderate-to-vigorous physical activity and muscle strength training are assessed in the study.
- Cross-sectional and self-reported study among a convenience sample.
- Biases were minimised by weighing data using UK Census data.
- We tested robustness of findings in sensitivity analyses.

For peer review only

Introduction

Physical activity has been negatively affected by the Covid-19 pandemic, which could have major implications for general health and Covid-19 outcomes[1,2,3]. As the Covid-19 pandemic continues and new restrictions may be introduced with new waves of the pandemic, it is paramount to understand how physical activity, and especially aerobic and muscle-strength training, were affected during the early stages of the pandemic, and which groups may require most support.

Adults who engage in less than 30 minutes of moderate physical activity per week are considered to be inactive [4,5]. In the UK [6] and other countries (e.g. the US [7]) guidelines for physical activity frequency and duration are complex, and often vary by age or clinical profile of the target population. However, a consensus exists that for the best health outcomes adults are recommended to engage in moderate-to-vigorous intensity aerobic physical activity (MVPA; i.e. activities that increase heart rate and make one feel warmer) for at least 150 minutes/week, as well as in muscle-strengthening activity (MSA; e.g., strength/resistance training) for at least two days/week[8]. MVPA and MSA lower morbidity and mortality both independently and combined [9-14]. Importantly, improving MVPA or MSA requires different strategies and interventions at both population and individual level, thus both are important outcomes to research[15].

The Covid-19 pandemic [15] and social distancing measures introduced in the UK and other countries limited many opportunities to engage in physical activity[15,16]. The first national lockdown in the UK was introduced on 23rd March 2020. As part of the lockdown measures, sports centres and gyms were temporarily closed and team sports banned, while access to outdoors spaces (e.g., remote national parks) was greatly reduced (i.e., some parks were closed or were only available to local visitors). By contrast, certain lockdown measures introduced in the UK could enable exercising, including the introduction of remote working that offered greater flexibility in scheduling and engaging in exercising at home or in local parks [15]. Furthermore, exercising outdoors was listed as one of the few activities that were still permitted even during the strictest lockdown in the UK, alongside shopping for essential items or medicine and going to work.

A large systematic review[17] showed that the majority of studies published by October 2020 reported declines in different measures of physical activity and increases in sedentary behaviour during the initial stages of Covid-19 pandemic[17,18]. However, some studies found that a small minority of adults increased their activity[19], suggesting it is important to identify factors associated with both the detrimental and beneficial changes to activity levels. Female gender, lower income, older age, health conditions, perceived risks, poorer mental health and non-white ethnicity [20,21 19,22-25] were all associated with lower physical activity levels. However, few studies accounted for other factors that could be relevant during the pandemic, such as having access to indoor and outdoor exercising space or household makeup[19].

Furthermore, research on how the Covid-19 pandemic has affected physical activity to date has often focused on moderate-to-vigorous physical activity (MVPA), without consideration of muscle-strengthening activity (MSA), or failed to distinguish between MVPA and MSA [19,26,17,27], or assessed only perceived, qualitative, changes to physical activity levels with respect to pre-pandemic levels (e.g. participants were asked to report if they exercised ‘more’ or ‘less’ since before the pandemic started rather than providing detailed information about MVPA and MSA responses)[28,29]. Moreover,

For peer review only

few studies that assessed and reported declines in MVPA and MSA, for example among adults in Russia [30] and in Italy [31], did not report on factors associated with these specific changes.

In order to identify groups in the population that may require targeted physical activity interventions during future periods of lockdowns or restrictions to physical activity, it is important to research the patterns and factors associated with changes in levels of both MVPA and MSA that are recommended by the WHO.

Aims

We characterised separately and combined MVPA and MSA levels during the periods *before* and *since* the Covid-19 pandemic had started in the UK, with the latter covering the period of the first strictest UK lockdown up until 14th June 2020. The research questions (RQs) were:

- RQ1: What were the levels and changes in MVPA and MSA among UK adults from before to since Covid-19?
- What socio-demographic, environmental and health factors were associated with:
  RQ2: meeting WHO recommendations for MVPA and MSA since Covid-19?
  RQ3: (i) decrease in MVPA among those who were active, and (ii) increase in MVPA among those who were inactive from before to since Covid-19?
  RQ4: (i) decrease in MSA among those who were active, and (ii) increase in MSA among those who were inactive, from before to since Covid-19?

Methods

Study Design

The study involved cross-sectional data analysis from the baseline online survey of the HEalth BEhaviours during the COvid-19 pandemic (HEBECO) study (https://osf.io/sbgru/). Data were collected using REDCap software[32] at UCL. The protocol for the present data analysis was preregistered (https://osf.io/ejghs/). Departures from the protocol are explained in Supplementary Materials 1. The results from all the pre-registered analyses not reported here are available elsewhere: https://osf.io/2ujxq/. The reporting follows the STROBE guidelines.[33]

Participants and Procedures

Recruitment involved a UK-wide campaign including paid and unpaid posts on social media and information shared through the networks of Cancer Research UK, Public Health England, other charities, sports clubs, universities, and local authorities. Interested participants were directed to the study website where they were shown a participant information sheet, provided informed consent, and then completed the online survey. Participants with complete data on the measures of interest for this study, and who enrolled between 5th May 2020 (start of the data collection for all study variables, during the first UK lockdown) until 14th June 2020 (the last day prior to easing of restrictions of the first UK lockdown) were included in the analytical sample.

For peer review only - http://bmjopen.bmj.com/site/about/guidelines.xhtml

Patient and Public Involvement

Patients or members of the public were not involved in the HEBECO baseline design, but the survey was shared with researchers at CRUK and Public Health England Behavioural Insights function in order to collect their feedback and suggestions for questions and answers wording, where relevant (e.g., when questions were not validated). Participants in the HEBECO study were encouraged to leave comments about specific behaviours and the survey itself, and these were incorporated into subsequent follow-up surveys (not the present study), where possible and relevant.

Measures

For the wording of all measures see https://osf.io/bja7g/ and Supplementary Materials 2. At baseline participants indicated the time when their lives started to be affected by the Covid-19 pandemic in any way, which was used as an anchor to assess before/since the Covid-19 pandemic onset MVPA and MSA. Almost half (44.0%, weighted) of adults selected the second half of March, followed by 26.9% who selected the first half of March.

Meeting WHO recommendations for MVPA and MSA

The before/since Covid-19 MVPA and MSA were measured using the following questions[14,34]. For MVPA, participants were first asked "In the month Before/Since Covid-19, on average, HOW MANY TIMES PER WEEK did you do at least 15 MINUTES or more of moderate or vigorous AEROBIC PHYSICAL ACTIVITY? Examples: brisk walk, jogging, dancing, cycling for recreation or commute, swimming, team or racket sports." The answers were capped at 14+, equivalent to at least twice per day. The 15 minute period was selected as it corresponds to the minimum amount of PA needed for mortality reduction and extending lifespan[35]. Those who engaged in at least one such session were asked "In the month BEFORE/Since COVID-19, HOW LONG (in MINUTES) was your average session of moderate or vigorous AEROBIC PHYSICAL ACTIVITY? Do NOT include strength training." Total weekly MVPA was computed by multiplying the session number by the minutes.

MSA activity was assessed by a single question: "Before/Since Covid-19, on average, on HOW MANY DAYS PER WEEK did you do STRENGTH TRAINING? Examples: Pilates, push-ups, squats, yoga, and exercises involving free weights, weight machines or elastic band." The answers were capped at 4+ given that the recommendations are for 2 days of MSA per week.

Data on MVPA and MSA were used to categorise participants, separately for before/since Covid-19, into four groups based on whether they met the WHO recommendations for MSA (at least 2 days/week) and MVPA (≥150 minutes/week)[8]: meeting neither, meeting MSA only, meeting MVPA only, or meeting recommendations for both MSA and MVPA.

Change in MVPA and MSA

Changes in MVPA (RQ 3) and MSA (RQ 4) were calculated by subtracting the before Covid-19 levels of MSA or MVPA from the since Covid-19 levels. Participants were categorised into: maintenance (change in MVPA <20 min; change in MSA=0), decrease or increase in activity (MVPA by ± ≥20min;

For peer review only - http://bmjopen.bmj.com/site/about/guidelines.xhtml

MSA by ± ≥1 day). Dichotomous variables were then created: decreased (vs maintained/increased) and increased (vs maintained/decreased) MVPA and MSA levels.

To minimise the bias due to ceiling and floor effects when assessing changes to MVPA and MSA, the analyses for RQs 3 and 4 were conducted after stratifying for before Covid-19 MVPA and MSA. Participants were categorised into: inactive MVPA (<30min of MVPA/week[4,5]) or MSA (0 days of MSA/week) and active (≥30 min/week of MVPA; and ≥ 1 day/week of MSA) before Covid-19.

Participants who were active since Covid-19 were also asked "Do you do the same FORM of exercise as you did before the COVID-19? (even if it is in a different location)?" The answer options were: None of the same/some of the same/about half the same/mostly the same/exactly the same.

Explanatory factors and correlates

Socio-demographic characteristics and living conditions assessed were: gender (participants were asked to select which answer they identify with most, and the answers were dichotomised into: female/all other), ethnicity (white/non-white); education (post-16-years of age/other); employed (yes/no); furloughed/laid off (yes/no); income (low-middle <50 000 GBP/high ≥50 000 GBP/prefer-not-to-say); age (in decades was entered as a continuous variable where it met assumptions, or as a 3-level categorical variable: ≤34, 35-64, 65+).

We assessed health behaviours, health and living conditions that could impact on MVPA or MSA levels: self-reports of any condition that limited physical activity (yes/no); body mass index (BMI[34]: normal and underweight ≤24.99/overweight 25-29.99/ obese ≥30); smoking status (current smoker vs not)[36]; weekly frequency of alcohol drinking[37]; deterioration in psychological wellbeing from before to since Covid-19 (yes/no); participants' perceived risk of Covid-19 to their health ('no or minor risk'/other); access to a garden or balcony big enough to exercise in comfortably (yes/no); access to a public park/green space that is within a walking distance and open during Covid-19 (yes/no); living with children aged ≤15 (yes/no); living with vulnerable persons (persons over the age of 70, in poor health, or who may be vulnerable to Covid-19; yes/not). Participants were asked about the status of their isolation at the time of completing the survey: "Which type of COVID-19 induced isolation are you experiencing?" (i) Total isolation/quarantine (not leaving the house for ANY reasons, not even to buy groceries or medications or to exercise); (ii) Some isolation (not leaving the house EXCEPT to buy essential items, such as groceries or medication or to exercise); (iii) General isolation but still go out to work (still go out to work and to buy essential items, such as groceries or medications or to exercise); (iv) No isolation (I am free to leave the house whenever I like, including participating in social gatherings or group sports, going to a bar or restaurant, travelling for leisure). The answers were dichotomized into: (i) total isolation/ (ii-iv) all other.

The regression models described below included two time co-variates to account for weather changes: enrolment time (up until 15th May, the second half of May, the first half June), and time when Covid-19 started to affect individuals (before mid-March/later) as these could affect exercise levels.

Analysis

For peer review only

Analyses were conducted in SPSS 26 (IBM Corp. Released 2019. IBM SPSS Statistics for Windows, Version 26.0. Armonk, NY: IBM Corp) with the data weighted using the 2018 Census and APS mid-year estimates for age, gender, ethnicity, country of living and household income. The analysis used weights trimmed to top 98th percentile to minimise the impact of extremely high weights [38]. Differences between the included and excluded participants were assessed with chi-squared for categorical and t-tests for continuous data.

For RQ1, descriptive statistics were computed to characterise the levels of MVPA, MSA, inactivity before/since the pandemic and changes in exercise form. For RQ2, univariate and fully adjusted multinomial regression models were computed. For RQ3 and RQ4 the sample was stratified using before pandemic MVPA or MSA levels to assess outcomes of interest using univariate and adjusted logistic regression models. All independent variables were entered into the models together as they were selected due to their previously established or theoretical importance. Sensitivity analyses involved replicating the analyses using unweighted data to check for the robustness of the results, and for RQ3 using different cut-off values of 15mins and 30mins. Family-wise error was corrected for by using the Benjamini-Hochberg procedure separately for each research question[39].

Results

Out of 2,992 participants who were included in the HEBECO baseline sample, 2,657 adults (weighted N=2,442) had complete data for this study and were included in the analytical sample. Table 1 presents comparisons of the excluded and included sample. The included weighted sample comprised 52% females, 90.5% of white ethnicity, and 67.0% with high school education or higher.

The sections below summarise the significant results for the weighted fully adjusted analyses. Results from univariable analyses are reported in Supplementary Materials 3, and from sensitivity analyses in Supplementary Materials 4 and 5. The results of the sensitivity analyses did not change the direction or magnitude of the results.

Research Question 1: Changes in MVPA and MSA before to since Covid-19.

Table 2 presents data on physical activity levels before and since Covid-19. Before Covid-19 17.6% of adults engaged in no MVPA and MSA, 55.3% engaged in no MSA, and 19.4% engaged in no MVPA. Just under 15% of adults met the recommended levels of both MVPA and MSA before and since Covid-19. The proportion of those who had no MVPA or MSA activity increased minimally (17.6% to 22.1%) which was primarily driven by declines in MVPA. Among those who were active since Covid-19, 41.5% continued with mostly or exactly the same form of activity as before Covid-19.

From before to since Covid-19 similar proportions of adults maintained (30.4%), decreased (36.2%) or increased (33.5%) their weekly MVPA. Among those who were active before Covid-19, 46.7% decreased MVPA. The decrease was of on average 219min/week. Among those who were inactive before Covid-19, 29.7% increased MVPA, with an average increase of 144min/week.

Maintenance of MSA was relatively more common (61.6%), with 18.2% decreasing and 20.2% increasing the number of days they engaged in MSA. Among those who were active before Covid-19 (40.2%), 45.1% decreased MSA, with an average decrease of 1.9 days/week. Among those who

For peer review only - http://bmjopen.bmj.com/site/about/guidelines.xhtml

For peer review only

engaged in no MSA before Covid-19 (59.8%), 16.4% increased, with an average increase of 2.5 days/week.

Research Question 2: Predictors of meeting WHO recommendations

Table 3 presents results from fully adjusted models. Being aged 65+ (adjusted odds ratio 95% CI, aOR=0.53,0.32-0.87), having a lower pre-Covid-19 household income (aOR=0.65,0.46-0.91), having a condition limiting physical activity (aOR=0.44,0.28-0.71), having obesity (aOR=0.49,0.33-0.73), living in total isolation (aOR=0.44,0.23-0.83) and deterioration in psychological wellbeing (aOR=0.56-0.43-0.73) were significantly associated with lower odds, while having at least high school education (aOR=1.57,1.14-2.17) or meeting the WHO recommended levels of MVPA (aOR=3.88,2.64-5.70), MSA (aOR=6.38,4.26-9.54) or both before Covid-19 (aOR=16.11,11.24-23.07), were significantly associated with greater odds of meeting both MVPA and MSA WHO guidelines since Covid-19, as compared with not meeting either.

Having a condition that limited physical activity (aOR=0.43,0.29-0.63), being in total isolation (0.26,0.14-0.50) and deterioration in psychological wellbeing (aOR=0.71,0.56-0.89) were significantly associated with lower odds, and meeting the WHO recommended levels of MVPA (aOR=7.57,5.82-3.39) or both MVPA and MSA (aOR=2.31,1.59-3.39) was significantly associated with greater odds of meeting MVPA only since Covid-19. Being aged 35-64 (aOR=0.56,0.38-0.82), and having obesity (BMI≥30; aOR=0.37-0.24,0.56) was associated with lower odds, while higher education, and meeting both MVPA and MSA or only MSA recommendations before Covid-19 was associated with greater odds of meeting the recommendations for MSA only since Covid-19.

RQ 3: Associations with changes in MVPA

Older age (aOR=0.91,0.85-0.98), white ethnicity (aOR=0.62,0.44-.86), post-16 years of age education (aOR=0.73,0.58-0.91) and access to a garden/balcony to exercise comfortably in (aOR=0.75,0.60-0.94) were significantly less likely to decrease MVPA activity (Table 4). Those with conditions limiting PA (aOR=1.74,1,27-2.39), living in total isolation (aOR=3.81,2.33-6.23), and experiencing a deterioration in psychological wellbeing during lockdown (aOR=1.40,1.15-1.71) were significantly more likely to decrease MVPA activity from before Covid levels.

White ethnicity, being employed during Covid-19, and living in total isolation were significantly associated with lower odds of increasing MVPA among this group. At least high school education and living with children was significantly associated with higher odds of increasing MVPA activity.

RQ 4: Associations with changes in MSA

Being employed during lockdown (aOR=1.81,1.34-2.46) and having overweight (BMI 25-29.99; aOR=1.88,1.39-2.55) or obesity (BMI ≥30; aOR=23.38,2.23-5.14) were significantly associated with higher odds of decreasing MSA activity. Older age (35-64, aOR=0.22,0.15-0.33; and 65+, aOR=0.34,0.20-0.58) and deterioration in psychological wellbeing (aOR=0.62,0.46-0.83) were significantly associated with lower odds of increasing MSA. See Table 5 for details.

For peer review only

Discussion

This study shows that the first Covid-19 lockdown in the UK affected the levels of moderate-to-vigorous physical activity (MVPA) and muscle-strengthening activity (MSA) differently, with several unique factors associated with engagement in and changes in these two activity types. Furthermore, although MVPA and MSA declined among a substantial proportion of UK adults during the first lockdown, which is in line with a majority of studies reporting declines across different measures of physical activity [17,20], the present findings show that a substantial minority of UK adults maintained or increased MVPA or MSA levels. These findings underline the importance of measuring MVPA and MSA, and their correlates, separately.

Meeting WHO recommendations for MVPA and MSA before and since Covid-19

Adherence to WHO recommendations for both MVPA and MSA before and since Covid-19 was low – at about 15%, which is lower than 26% reported in the Health Survey for England (HSE) for 2012-2016[40]. Also, in comparison to the findings from HSE (36%), fewer of the adults in this study (21%) met MVPA only recommendations, but more met MSA only recommendations (12% in this study vs 1% in HSE). The current MSA levels are reflecting other epidemiological data [9 41]. The differences between the HEBECO and HSE findings could be due to the differences in the measures of MSA and MVPA used, participant characteristics (e.g., in comparison to the HSE study [40], the HEBECO sample included more educated adults who may be more aware of the benefits of MSA), or reflect genuine changes to the patterns of physical activity in the UK across time. In line with other studies [40,42], older adults (aged 65+), adults who were obese, those with before Covid-19 household income below 50 000 GBP and those who had conditions limiting physical activity were at greater risk of not meeting the WHO recommendations for both MVPA and MSA. These groups should be targeted by future interventions aimed at increasing physical activity. Such targeting could include dedicated reach out campaigns of more generic interventions among these groups, as well as developing interventions that address the unique circumstances in which these populations can engage in physical activity.

Meeting the recommendations for MVPA and MSA before Covid-19 was strongly predictive of meeting them since Covid-19. Additionally, adults who remained active tended to maintain at least similar levels and forms of the exercises as in the pre-pandemic period. Thus, as UK adults remain relatively consistent in their behaviour, even during the lockdown, this study highlights the need to especially support those with low activity levels to develop healthy exercising routines.

Changes to MVPA and MSA levels from before to since the Covid-19 pandemic start

Although group level data suggest little change in physical activity before to since Covid-19, especially in MSA, about a third of adults decreased and another third increased their MVPA from before to since Covid-19. Those who changed MVPA decreased or increased their MVPA levels by over three and two hours per week, respectively. Those who changed MSA either gained or lost two days of MSA per week. These changes are substantial and likely to affect health outcomes.

Decreases in MVPA were found among other adults samples in the UK[19], Italy [26] and Spain[27]. However, the finding that over 70% of UK adults maintained or increased their MVPA could be at least partially attributed to the UK government's consistent lockdown policies that allowed leaving the house for exercising, which is in contrast to other countries with more restrictive policies[27,43].

The maintenance of MSA levels could be at least partially explained by the low before Covid-19 MSA levels. Additionally, many MSA exercises can be performed with no equipment and at one's home (e.g., pilates, push-ups) and thus may be less affected by social distancing measures. This is further supported by the finding that total isolation (i.e., not leaving the house for any reason) was associated with MVPA but not MSA levels.

Factors associated with changes and maintenance of MVPA and MSA since Covid-19

Older adults were more likely to maintain their before Covid-19 MVPA and MSA levels, including inactivity. Due to more established routines and lower baseline physical activity, this group might have been less affected by the lockdown restrictions, such as gym closures or team sports bans[44].

MVPA inactivity was more commonly maintained by those who were employed or living in total isolation. Such adults likely had fewer opportunities or flexibility to exercise. Additionally, those who had a health condition limiting physical activity and those living in total isolation tended to decrease MVPA. Factors that were associated with maintaining MVPA activity since Covid-19 were white ethnicity, higher education and having a garden or balcony large enough to exercise.

Increasing MVPA levels among those who were previously inactive was most common among those with higher education, which is in line with prior studies [45], and those who lived with children. Caring for children might have promoted some forms of MVPA, including outdoors, particularly as parents became responsible for children's physical education during school lockdown closures.

In terms of changes in MSA levels, older adults were at higher risk of maintaining MSA inactivity, while adults with higher BMI levels were at greater risk of decreasing MSA from before to since Covid-19. Adults with higher BMI reported lower levels of physical activity during lockdowns in other studies as well.[28] Therefore, both older adults and those with higher BMI should be among priority groups targeted with MSA interventions, particularly as MSA can bring important clinical benefits to these two groups[46-49]. Other priority groups for MSA training are those with lower household income and lower educational attainment.

Finally, as found previously[24], psychological wellbeing was associated with physical activity levels. In the present study deterioration in psychological wellbeing was associated with not meeting guidelines for MVPA or decreasing MVPA levels, but not with MSA. Due to the cross-sectional design, causality cannot be assumed as poorer psychological wellbeing (e.g., lower mood, anxiety, stress) can be both a predictor and a consequence of low exercise levels[50,51]. However, interventions aimed at improving physical activity are likely to improve mental health as well[50-52].

Strengths and limitations

The study is one of few that assessed the levels of, and changes in, both MVPA and MSA during the first UK lockdown. The study drew on previously used measures of MVPA and MSA[53], which were supplemented by images to clarify exercise types. This study also benefits from measuring and including in the models a large number of correlates and covariates that could act as potential confounding variables. Several sensitivity analyses were conducted to test the robustness of findings. The key limitations are that this was a cross-sectional study among a self-selected sample that relied on self-report and recall that are prone to bias. This could lead to over-reporting of physical activity and the possibility that the wider population had an even poorer physical activity profile during the pandemic. A small sample (11%) of all HEBECO baseline participants were excluded from the present

For peer review only - http://bmjopen.bmj.com/site/about/guidelines.xhtml

analyses due to incomplete data (e.g., attrition before reporting physical activity).However, while some of the characteristics of the excluded participants could be associated with greater physical activity (i.e., younger age, non-female gender, lower BMI), others may be associated with lower physical activity levels (i.e., not being in employment, not having access to garden or balcony, being a smoker, or being of non-White ethnicity). On balance, the present findings therefore may be reflective of the changes in physical activity at the population level in the UK. Finally, the unfolding of the Covid-19 pandemic has coincided with the seasonal change from winter to spring. Without a true baseline from the same period in 2019, it is not possible to tease apart the effect of weather change from that of the pandemic.

Implications

This study adds to the growing body of literature that emphasises the importance of researching MSA in addition to the more commonly assessed MVPA[9]. It also shows that although the first UK lockdown adversely affected many individuals, a considerable proportion of the adults managed to maintain or even improve MVPA and MSA. These findings suggest that future interventions and policies should not only aim to prevent deterioration in physical activity but also try to capitalise on the opportunities brought by lifestyle disruption to support increases in activity levels. We have at our disposal many interventions and tools, including those that are digital- or technology-based, which could be offered at relatively low cost to large target populations while adhering to even the strictest lockdown measures. Efforts should focus now on identifying acceptable and effective ways of delivering such interventions for both MVPA and MSA, especially among the groups in the population that are at risk of poorer PA outcomes and given the ongoing challenges brought by the Covid-19 pandemic.

Conclusions

The findings highlight social inequalities in how the first lockdown in the UK has affected physical activity levels, with differential impact on aerobic and strength training. Dedicated interventions are needed to support MVPA and MSA, especially among those who are older, have lower income, and have higher body mass index with general low activity levels.

For peer review only

Footnotes

- **Contributorship statement:** AH conceived the idea in consultation with PL, AF and LS. AH and VS analysed the data. All authors (AH, VS, AF, DK, LS and PL) interpreted the data. AH drafted the first version of the manuscript. All authors critically revised the manuscript and approved the final draft before submission.

- **Funding:** This project is partially funded by an ongoing Cancer Research UK Programme Grant to UCL Tobacco and Alcohol Research Group (C1417/A22962) and by SPECTRUM, a UK Prevention Research Partnership Consortium (MR/S037519/1). Dr Lally's salary is paid for by a Yorkshire Cancer Research grant (UCL420) and a Cancer Research UK project grant (C43975/A27498)

- **Competing interests:** None declared.
- **Patient consent for publication:** Obtained.
- **Ethics approval:** The study was approved by the by UCL Research Ethics Committee at the UCL Division of Psychology and Language Sciences (PaLS) (CEHP/2020/579; as part of the larger programme of work: The optimisation and implementation of interventions to change behaviours related to health and the environment).
- **Data Sharing Statement:** Study and additional data can be accessed by contacting lion.shahab@ucl.ac.uk and a.herbec@ucl.ac.uk.

- **Acknowledgements:** We are grateful to all participants who have been supporting our research. We would like to thank Public Health England, and particularly members of the Behavioural Insights at Public Health England for providing feedback on the survey wording. We would like to thank Public Health England, Cancer Research UK, local authorities, Mayors' offices, as well as charities and other organisations in the UK, including the Asthma UK and British Lung Foundation Partnership, for supporting our recruitment campaign.

References

1. Warburton DER, Bredin SSD. Health benefits of physical activity: a systematic review of current systematic reviews. *Curr. Opin. Cardiol.* 2017;32(5):541-556.
2. Woods JA, Hutchinson NT, Powers SK, et al. The COVID-19 pandemic and physical activity. *Sports Medicine and Health Science.* 2020;2(2):55-64.
3. Shur NF, Johns D, Kluzek S, Peirce N. Physical inactivity and health inequality during coronavirus: a novel opportunity or total lockdown? *BMJ Open Sport & Exercise Medicine.* 2020;6(1):e000903.
4. Public Health England. Physical inactivity levels in adults aged 40 to 60 in England. 2017; Accessed on 20th September 2020 from: https://www.gov.uk/government/publications/physical-inactivity-levels-in-adults-aged-40-to-60-in-england/.
5. Health Survey for England. Health Survey for England 2016 - Physical activity in adults. 2017; Accessed on 20th Sept 2020 from: http://healthsurvey.hscic.gov.uk/media/63730/HSE16-Adult-phy-act.pdf
6. Department of Health & Social Care. Physical activity guidelines: UK Chief Medical Officers' report. 2020.

For peer review only - http://bmjopen.bmj.com/site/about/guidelines.xhtml

7. US Department of Health and Human Services. Physical Activity Guidelines for Americans, 2nd edition. 2018.
8. WHO. Physical activity. . 2018; Accessed on 20th November 2020 from https://www.who.int/news-room/fact-sheets/detail/physical-activity.
9. Bennie JA, Shakespear-Druery J, De Cocker K. Muscle-strengthening Exercise Epidemiology: a New Frontier in Chronic Disease Prevention. *Sports medicine - open.* 2020;6(1):40.
10. Kamada M, Shiroma EJ, Buring JE, Miyachi M, Lee IM. Strength Training and All-Cause, Cardiovascular Disease, and Cancer Mortality in Older Women: A Cohort Study. *Journal of the American Heart Association.* 2017;6(11).
11. Stamatakis E, Lee IM, Bennie J, et al. Does Strength-Promoting Exercise Confer Unique Health Benefits? A Pooled Analysis of Data on 11 Population Cohorts With All-Cause, Cancer, and Cardiovascular Mortality Endpoints. *American journal of epidemiology.* 2018;187(5):1102-1112.
12. Zhao G, Li C, Ford ES, et al. Leisure-time aerobic physical activity, muscle-strengthening activity and mortality risks among US adults: the NHANES linked mortality study. *Br. J. Sports Med.* 2014;48(3):244-249.
13. Zhao M, Veeranki SP, Magnussen CG, Xi B. Recommended physical activity and all cause and cause specific mortality in US adults: prospective cohort study. *BMJ (Clinical research ed.* 2020;370:m2031.
14. Bennie JA, De Cocker K, Teychenne MJ, Brown WJ, Biddle SJH. The epidemiology of aerobic physical activity and muscle-strengthening activity guideline adherence among 383,928 U.S. adults. *The international journal of behavioral nutrition and physical activity.* 2019;16(1):34.
15. Dwyer MJ, Pasini M, De Dominicis S, Righi E. Physical activity: Benefits and challenges during the COVID-19 pandemic. *Scand. J. Med. Sci. Sports.* 2020;30(7):1291-1294.
16. Ricci F, Izzicupo P, Moscucci F, et al. Recommendations for Physical Inactivity and Sedentary Behavior During the Coronavirus Disease (COVID-19) Pandemic. *Frontiers in public health.* 2020;8:199.
17. Stockwell S, Trott M, Tully M, et al. Changes in physical activity and sedentary behaviours from before to during the COVID-19 pandemic lockdown: a systematic review. *BMJ Open Sport Exerc Med.* 2021;7(1):e000960.
18. Ammar A, Brach M, Trabelsi K, et al. Effects of COVID-19 Home Confinement on Eating Behaviour and Physical Activity: Results of the ECLB-COVID19 International Online Survey. *Nutrients.* 2020;12(6).
19. Rogers NT, Waterlow N, Brindle HE, et al. Behavioural change towards reduced intensity physical activity is disproportionately prevalent among adults with serious health issues or self-perception of high risk during the UK COVID-19 lockdown. . *MedRxiv* 2020.
20. Sport England. Active Lives Adult Survey: November 2019/20 Report. 2021.
21. Dunton GF, Wang SD, Do B, Courtney J. Early effects of the COVID-19 pandemic on physical activity locations and behaviors in adults living in the United States. *Preventive medicine reports.* 2020;20:101241.
22. Meyer J, McDowell C, Lansing J, et al. Changes in physical activity and sedentary behaviour due to the COVID-19 outbreak and associations with mental health in 3,052 US adults. *Cambridge Open Engage.* 2020.
23. Smith L, Jacob L, Butler L, et al. Prevalence and correlates of physical activity in a sample of UK adults observing social distancing during the COVID-19 pandemic. *BMJ Open Sport & Exercise Medicine.* 2020;6(1):e000850.
24. Jacob L, Tully MA, Barnett Y, et al. The relationship between physical activity and mental health in a sample of the UK public: A cross-sectional study during the implementation of COVID-19 social distancing measures. *Mental health and physical activity.* 2020;19:100345.
25. Constandt B, Thibaut E, De Bosscher V, Scheerder J, Ricour M, Willem A. Exercising in Times of Lockdown: An Analysis of the Impact of COVID-19 on Levels and Patterns of Exercise

For peer review only

among Adults in Belgium. *International journal of environmental research and public health.* 2020;17(11).

26. Maugeri G, Castrogiovanni P, Battaglia G, et al. The impact of physical activity on psychological health during Covid-19 pandemic in Italy. *Heliyon.* 2020;6(6):e04315.
27. López-Bueno R, Calatayud J, Andersen LL, et al. Immediate Impact of the COVID-19 Confinement on Physical Activity Levels in Spanish Adults. *Sustainability.* 2020;12(14):5708.
28. Robinson E, Boyland E, Chisholm A, et al. Obesity, eating behavior and physical activity during COVID-19 lockdown: A study of UK adults. *Appetite.* 2021;156:104853.
29. Spence JC, Rhodes RE, McCurdy A, Mangan A, Hopkins D, Mummery WK. Determinants of physical activity among adults in the United Kingdom during the COVID-19 pandemic: The DUK-COVID study. *British journal of health psychology.* 2021;26(2):588-605.
30. Kontsevaya AV, Mukaneeva DK, Myrzamatova AO, Okely AD, Drapkina OM. Changes in physical activity and sleep habits among adults in Russian Federation during COVID-19: a cross-sectional study. *BMC public health.* 2021;21(1):893.
31. Fuzeki E, Schroder J, Carraro N, et al. Physical Activity during the First COVID-19-Related Lockdown in Italy. *International journal of environmental research and public health.* 2021;18(5).
32. Harris PA, Taylor R, Thielke R, Payne J, Gonzalez N, Conde JG. Research electronic data capture (REDCap)--a metadata-driven methodology and workflow process for providing translational research informatics support. *J Biomed Inform.* 2009;42(2):377-381.
33. von Elm E, Altman DG, Egger M, et al. Strengthening the Reporting of Observational Studies in Epidemiology (STROBE) statement: guidelines for reporting observational studies. *BMJ (Clinical research ed.* 2007;335(7624):806-808.
34. Churilla JR, Johnson TM, Richardson MR, Williams BD, Rariden BS, Boltz AJ. Mode of physical activity participation by body mass index: 2015 behavioural risk factor surveillance system. *Res. Sports Med.* 2018;26(2):147-157.
35. Wen CP, Wai JPM, Tsai MK, et al. Minimum amount of physical activity for reduced mortality and extended life expectancy: a prospective cohort study. *The Lancet.* 2011;378(9798):1244-1253.
36. Kaczynski AT, Manske SR, Mannell RC, Grewal K. Smoking and Physical Activity: A Systematic Review. *American Journal of Health Behavior.* 2008;32(1).
37. Leasure JL, Neighbors C, Henderson CE, Young CM. Exercise and Alcohol Consumption: What We Know, What We Need to Know, and Why it is Important. *Frontiers in psychiatry.* 2015;6:156.
38. Potter FA. Study of Procedures to Identify and Trim Extreme Sample Weights. . *Proceedings of the Survey Research Methods Section, American Statistical Association.* 1990:225-230.
39. Benjamini Y, Hochberg Y. Controlling the False Discovery Rate: A Practical and Powerful Approach to Multiple Testing. *Journal of the Royal Statistical Society: Series B (Methodological).* 1995;57(1):289-300.
40. De Cocker K, Teychenne M, White RL, Bennie JA. Adherence to aerobic and muscle-strengthening exercise guidelines and associations with psychological distress: A cross-sectional study of 14,050 English adults. *Prev Med.* 2020;139:106192.
41. Bennie JA, De Cocker K, Smith JJ, Wiesner GH. The epidemiology of muscle-strengthening exercise in Europe: A 28-country comparison including 280,605 adults. *PloS one.* 2020;15(11):e0242220.
42. Salman A, Ukwaja KN, Alkhatib A. Factors Associated with Meeting Current Recommendation for Physical Activity in Scottish Adults with Diabetes. *International journal of environmental research and public health.* 2019;16(20).
43. DW. Coronavirus: What are the lockdown measures across Europe? 2020; Accessed 20th Sept 2020 from: https://www.dw.com/en/coronavirus-what-are-the-lockdown-measures-across-europe/a-52905137.

For peer review only - http://bmjopen.bmj.com/site/about/guidelines.xhtml

44. Sun F, Norman IJ, While AE. Physical activity in older people: a systematic review. *BMC public health.* 2013;13:449.
45. Shaw BA, Spokane LS. Examining the association between education level and physical activity changes during early old age. *J. Aging Health.* 2008;20(7):767-787.
46. Kraschnewski JL, Sciamanna CN, Poger JM, et al. Is strength training associated with mortality benefits? A 15year cohort study of US older adults. *Prev Med.* 2016;87:121-127.
47. Guizelini PC, de Aguiar RA, Denadai BS, Caputo F, Greco CC. Effect of resistance training on muscle strength and rate of force development in healthy older adults: A systematic review and meta-analysis. *Exp. Gerontol.* 2018;102:51-58.
48. Said MA, Abdelmoneem M, Almaqhawi A, Hamid Kotob AA, Alibrahim MC, Bougmiza I. Multidisciplinary approach to obesity: Aerobic or resistance physical exercise? *Journal of exercise science and fitness.* 2018;16(3):118-123.
49. Willis LH, Slentz CA, Bateman LA, et al. Effects of aerobic and/or resistance training on body mass and fat mass in overweight or obese adults. *J Appl Physiol (1985).* 2012;113(12):1831-1837.
50. Sharma A, Madaan V, Petty FD. Exercise for mental health. *Primary care companion to the Journal of clinical psychiatry.* 2006;8(2):106.
51. Mikkelsen K, Stojanovska L, Polenakovic M, Bosevski M, Apostolopoulos V. Exercise and mental health. *Maturitas.* 2017;106:48-56.
52. Callow DD, Arnold-Nedimala NA, Jordan LS, et al. The Mental Health Benefits of Physical Activity in Older Adults Survive the COVID-19 Pandemic. *Am. J. Geriatr. Psychiatry.* 2020;28(10):1046-1057.
53. Bennie JA, Pedisic Z, van Uffelen JG, et al. The descriptive epidemiology of total physical activity, muscle-strengthening exercises and sedentary behaviour among Australian adults--results from the National Nutrition and Physical Activity Survey. *BMC public health.* 2016;16:73.

For peer review only

Table 1. Sample characteristics (weighted) and comparison of participants who were included and excluded from the study (due to incomplete data).

	Included sample N=2442	Excluded sample N=350	*p*
Female	52.6%	48.7%	0.179
Age; M (SD)	50.0 (16.0)	42.7 (18.9)	<0.001
White ethnicity	90.5%	82.4%	<0.001
Household income ≥50 000	18.1%	15.5%	0.095
Income: <50 000 GBP	73.5%	73.1%	
Income: Prefer not to say	8.3%	11.5%	
Education: Highschool or higher	67.0%	69.1%	0.432
Employed	48.3%	40.7%	0.008
Laid-off/Furloughed	12.7%	15.2%	0.197
Condition limiting PA	16.3%	21.2%	0.035
BMI ≤24	43.2%	27.3%	<0.001
BMI: 25-29.99	35.1%	16.8%	
BMI: Obese: 30+	21.7%	8.4%	
BMI don't know/prefer not to say[a]	0%	47.6%	
Total isolation	7.8%	8.8%	0.541
Minor/no Covid-19 risk percept	33.3%	38.6%	0.069
Living with children	17.0%	15.7%	0.549
Living with vulnerable	15.1%	21.8%	0.001
Access garden/balcony	72.2%	62.6%	<0.001
Access green space	59.5%	52.4%	0.012
Time life affected by Covid-19 mid- March or sooner[b]	44.8%	53.3%	0.003
Enrolled from 1st June (ref)	4.4%	5.4%	0.404
Enrolled up until 15th May	50.1%	46.7%	
Enrolled second half of May	45.4%	47.9%	
Smoker	24.1%	38.9%	<0.001
Weekly alcohol drinking, none (ref)	21.8%	21.8%	0.920
Weekly or less	27.9%	26.9%	
More than weekly	50.3%	51.3%	
Deteriorated psychological wellbeing	54.1%	52.8%	0.708
Meeting WHO MVPA and MSA levels before Covid	15.6%	17.3%	0.218
Meeting only MVPA before	23.0%	22.0%	
Meeting only MSA before	13.0%	7.7%	
Meeting neither before	48.4%	53.0%	

[a]As per protocol, the participants who did not provide BMI data (e.g., they selected 'prefer not to say' or 'don't know' on the question on height or weight) but who provided other study data were to be included in the analyses but were ultimately excluded due to having missing data on other variables. [b]Time that participant's life started to be affected by Covid-19 in any way.

For peer review only

Table 2. Aerobic physical activity (MVPA) and strength training (MSA) before and since COVID-19.

	Before Covid-19[1]	Since Covid-19[1]
**No activity (0 sessions MVPA and 0 days MSA) % (N)**	17.6 (430)	22.1 (540)
**MVPA**		
MVPA 0 sessions % (N)	19.4 (473)	24.8 (604)
MVPA min/week among active; Median (IQR)	125 (210.0)	150 (220.0)
Mean (SD)	238.1 (424.0)	230.9 (398.8)
MVPA min/week among entire sample; Median (IQR)	90 (180.0)	90 (224.5)
Mean (SD)	196.2 (395.4)	179.8 (364.7)
**MSA**		
MSA % (N)		
0 das/week	55.3 (1557)	56.2 (1584)
1 day/week	11.1 (314)	9.6 (271)
2 days/week	10.4 (293)	7.9 (223)
3 days/week	9.0 (253)	7.6 (213
4+ days/week	7.0 (197)	11.3 (320)
MSA Median (IQR)	0 (2)	0 (2)
**Meet guidelines % (N)**		
Neither MVPA nor MSA	45.1 (1271)	44.6 (1256)
Meet MVPA (≥150 min/week) only	21.3 (599)	21.2 (598)
Meet MSA (≥2 days/week) only	11.7 (330)	11.9 (336)
Meets both MVPA and MSA	14.5 (410)	14.8 (416)
**Change in form of exercise among those who exercise % (N)**		
None of the same	-	17.1 (331)
Some of the same	-	32.7 (632)
About half the same	-	8.7 (168)
Mostly the same	-	26.0 (503)
Exactly the same	-	15.5 (299)

For peer review only

Table 3. Predictors of meeting both, MVPA only and MSA only WHO guidelines since Covid-19 in comparison to not meeting WHO guidelines for MVPA and MSA (reference, n=1178). Results from fully adjusted models using all variables listed on weighted data with BH FDR correction of p-values (significant in bold).

	Both MVPA and MSA (n=388)				MVPA only (n=570)				MSA only (n=306)			
	aOR	95% CI		*p*	aOR	95% CI		*p*	aOR	95% CI		*p*
Female	1.08	0.82	1.41	0.588	0.79	0.62	1.00	0.049	1.02	0.77	1.35	0.906
Age ≤34												
Age: 35-64	0.70	0.49	0.99	0.047	1.11	0.78	1.56	0.566	**0.56**	**0.38**	**0.82**	**0.003**
Age: 65+	**0.53**	**0.32**	**0.87**	**0.012**	1.10	0.71	1.70	0.676	0.99	0.60	1.61	0.961
White ethnicity	1.61	1.03	2.52	0.036	1.57	0.99	2.49	0.054	0.72	0.47	1.10	0.130
Household income ≥50 000												
Income: <50 000 GBP	**0.65**	**0.46**	**0.91**	**0.011**	0.71	0.52	0.96	0.028	1.13	0.76	1.67	0.556
Income: Prefer not to say	**0.44**	**0.25**	**0.77**	**0.004**	0.63	0.38	1.03	0.065	0.88	0.49	1.59	0.666
Education: post 16 years of age or higher	**1.57**	**1.14**	**2.17**	**0.006**	1.04	0.81	1.35	0.745	**1.74**	**1.24**	**2.45**	**0.002**
Employed	0.76	0.55	1.05	0.094	1.09	0.82	1.43	0.562	1.06	0.75	1.49	0.734
Laid-off/Furloughed	0.79	0.51	1.23	0.295	1.05	0.71	1.54	0.813	1.14	0.71	1.83	0.584
Condition limiting physical activity	**0.44**	**0.28**	**0.71**	**0.001**	**0.43**	**0.29**	**0.63**	**<0.001**	0.97	0.65	1.45	0.881
BMI ≤24												
BMI: 25-29.99	0.81	0.60	1.10	0.176	1.01	0.78	1.32	0.926	0.72	0.53	0.99	0.043
BMI: Obese: 30+	**0.49**	**0.33**	**0.73**	**<0.001**	0.87	0.64	1.19	0.394	**0.37**	**0.24**	**0.56**	**<0.001**
Total isolation	**0.44**	**0.23**	**0.83**	**0.011**	**0.26**	**0.14**	**0.50**	**<0.001**	0.83	0.49	1.40	0.478
Minor/no Covid-19 risk percept	1.00	0.74	1.34	0.977	1.20	0.93	1.55	0.167	0.94	0.68	1.30	0.718
Living with children	0.81	0.57	1.16	0.254	0.75	0.55	1.03	0.079	0.66	0.44	0.99	0.045
Living with vulnerable	1.17	0.82	1.68	0.395	0.86	0.61	1.20	0.366	0.87	0.58	1.30	0.499
Access garden/balcony to exercise comfortably in	1.10	0.81	1.51	0.533	1.02	0.79	1.33	0.861	0.80	0.58	1.09	0.159
Access green space within walking distance	1.27	0.96	1.69	0.096	1.31	1.03	1.66	0.028	0.91	0.68	1.23	0.548
Smoker	0.72	0.51	1.02	0.062	0.93	0.70	1.22	0.593	0.80	0.57	1.14	0.218
Weekly alcohol drinking, none (ref)												
Weekly or less	1.00	0.68	1.49	0.988	1.43	1.02	2.00	0.038	1.06	0.71	1.59	0.782
More than weekly	1.04	0.73	1.50	0.811	1.28	0.94	1.74	0.121	0.89	0.61	1.29	0.528
Deteriorated psychological wellbeing	**0.56**	**0.43**	**0.73**	**<0.001**	**0.71**	**0.56**	**0.89**	**0.003**	0.72	0.55	0.96	0.025
Meeting WHO PA[a] rec before Covid-19 (meeting neither=ref)												
Meeting both before	**16.11**	**11.24**	**23.07**	**<0.001**	**2.31**	**1.58**	**3.39**	**<0.001**	**3.72**	**2.45**	**5.62**	**<0.001**
Meeting only MVPA before	**3.88**	**2.64**	**5.70**	**<0.001**	**7.57**	**5.82**	**9.84**	**<0.001**	1.47	0.94	2.30	0.092
Meeting only MSA before	**6.38**	**4.26**	**9.54**	**<0.001**	0.74	0.44	1.25	0.264	**9.61**	**6.70**	**13.77**	**<0.001**

a=meeting World Health Physical activity recommendations before the first Covid-19 lockdown in the UK. Notes: All models were also adjusted for the time of enrolment and time when Covid-19 started to affect individuals in any way. Following the BH correction the highest p-value that met the threshold for significance was p=0.019

For peer review only - http://bmjopen.bmj.com/site/about/guidelines.xhtml

For peer review only

Table 4. Independent associations of change in MVPA (models fully adjusted using all the variables listed). Segmented analyses of active sample (before Covid-19 MVPA activity ≥30min; predicting decrease by ≥20min) and inactive sample (before Covid-19 MVPA activity <30min; predicting increase by ≥20min).

	Sample active (≥30min/week) before Covid-19 (weighted n=1857)				Sample inactive (<30min/week) before Covid-19 (weighted n=585)			
	Decrease (n=868) vs not				Increase (n=174) vs not			
	aOR	95% CI		*p*	aOR	95% CI		*p*
Female	1.22	1.00	1.49	0.047	1.56	1.02	2.38	0.040
Age (in decades)	**0.91**	**0.85**	**0.98**	**0.015**	**0.72**	**0.61**	**0.84**	**<0.001**
White ethnicity	**0.62**	**0.44**	**0.86**	**0.005**	1.01	0.44	2.33	0.982
Household income ≥50 000								
Income: <50 000 GBP	1.30	1.00	1.67	0.048	0.49	0.26	0.93	0.030
Income: Prefer not to say	1.22	0.81	1.85	0.335	0.36	0.14	0.93	0.034
Education: post 16 years of age or higher	**0.73**	**0.58**	**0.91**	**0.006**	**2.26**	**1.40**	**3.63**	**0.001**
Employed	0.86	0.69	1.07	0.180	**0.51**	**0.31**	**0.85**	**0.009**
Laid-off/Furloughed	1.05	0.76	1.47	0.750	1.30	0.72	2.37	0.385
Condition limiting PA	**1.74**	**1.27**	**2.39**	**0.001**	0.49	0.27	0.89	0.019
BMI ≤24								
BMI: 25-29.99	1.03	0.82	1.28	0.816	1.54	0.94	2.53	0.088
BMI: Obese: 30+	0.87	0.66	1.14	0.317	0.74	0.42	1.31	0.307
Total isolation	**3.81**	**2.33**	**6.23**	**<0.001**	**0.29**	**0.12**	**0.73**	**0.008**
Minor/no Covid-19 risk percept	0.99	0.80	1.23	0.926	1.20	0.76	1.92	0.436
Living with children	0.84	0.65	1.09	0.187	**2.32**	**1.27**	**4.24**	**0.006**
Living with vulnerable	0.78	0.59	1.02	0.074	0.90	0.51	1.59	0.716
Access garden/balcony to exercise comfortably in	**0.75**	**0.60**	**0.94**	**0.011**	0.72	0.46	1.13	0.157
Access green space within walking distance	0.82	0.67	1.01	0.059	1.10	0.72	1.67	0.665
Smoker	1.09	0.85	1.40	0.504	1.15	0.73	1.80	0.551
Weekly alcohol drinking, none (ref)								
Weekly or less	0.78	0.59	1.04	0.087	0.77	0.43	1.38	0.378
More than weekly	0.78	0.60	1.02	0.068	0.88	0.52	1.50	0.644
Deteriorated psychological wellbeing	**1.40**	**1.15**	**1.71**	**0.001**	1.09	0.72	1.66	0.682

Notes: All models were also adjusted for the time of enrolment and time when Covid-19 started to affect individuals in any way. Following the BH correction the highest p-value that met the threshold for significance was p=0.019.

For peer review only

Table 5. Independent associations of change in MSA (models fully adjusted using all the variables listed). Segmented analyses of active sample (before Covid-19 MSA activity ≥1 day/week; predicting decrease by ≥1 day/week) and less inactive sample (before Covid-19 MSA activity 0 days/week; predicting increase by ≥1 day/week). Findings from fully adjusted logistic regression models on weighted data and using BH FDR adjustment (significant in bold).

	Sample active (≥1 day/week) before Covid-19 (weighted n=985)				Sample inactive (0 days/week) before Covid-19 (weighted n=1456)			
	Decrease (n=444) vs not				Increase (n=239) vs not			
	aOR	95% CI		*p*	aOR	95% CI		*p*
Female	0.94	0.71	1.23	0.634	0.96	0.70	1.31	0.792
Age (in decades)	1.03	0.94	1.14	0.516	-	-	-	-
Age ≤34								
Age: 35-64	-	-	-	-	**0.22**	**0.15**	**0.33**	**<0.001**
Age: 65+	-	-	-	-	**0.34**	**0.20**	**0.58**	**<0.001**
White ethnicity	0.75	0.51	1.11	0.154	0.71	0.41	1.23	0.216
Household income ≥50 000								
Income: <50 000 GBP	1.11	0.80	1.55	0.535	0.67	0.45	0.99	0.047
Income: Prefer not to say	0.94	0.54	1.62	0.818	0.56	0.29	1.08	0.084
Education: post 16 years of age or higher	0.82	0.59	1.15	0.260	1.20	0.85	1.70	0.310
Employed	**1.81**	**1.34**	**2.46**	**<0.001**	1.08	0.75	1.55	0.671
Laid-off/Furloughed	1.34	0.85	2.12	0.205	1.30	0.80	2.10	0.289
Condition limiting PA	1.11	0.72	1.72	0.632	0.64	0.38	1.05	0.078
BMI ≤24.99								
BMI: 25-29.99	**1.88**	**1.39**	**2.55**	**<0.001**	0.89	0.63	1.26	0.512
BMI: Obese: 30+	**3.38**	**2.23**	**5.14**	**<0.001**	0.64	0.42	0.97	0.037
Total isolation	1.87	1.02	3.42	0.042	0.80	0.42	1.52	0.499
Minor/no Covid-19 risk percept	1.43	1.06	1.92	0.019	0.93	0.65	1.31	0.669
Living with children	1.01	0.71	1.44	0.964	1.48	0.94	2.31	0.088
Living with vulnerable	1.17	0.79	1.74	0.437	0.95	0.63	1.44	0.809
Access garden/balcony to exercise comfortably in	1.02	0.74	1.41	0.891	0.84	0.60	1.17	0.301
Access green space within walking distance	1.07	0.79	1.43	0.667	0.87	0.64	1.18	0.370
Smoker	0.63	0.43	0.91	0.013	1.04	0.74	1.46	0.828
Weekly alcohol drinking, none (ref)								
Weekly or less	1.11	0.74	1.65	0.620	1.13	0.73	1.76	0.580
More than weekly	1.26	0.86	1.85	0.237	1.06	0.71	1.59	0.786
Deteriorated psychological wellbeing	1.27	0.97	1.66	0.082	**0.62**	**0.46**	**0.83**	**0.002**

Notes: All models were also adjusted for the time of enrolment and time when Covid-19 started to affect individuals in any way. Following the BH correction the highest p-value that met the threshold for significance for was p=0.019.

For peer review only

Supplementary Materials 1-5

Manuscript Title: Correlates of and changes in aerobic physical activity and strength training before and after the onset of COVID-19 pandemic in the UK – findings from the HEBECO study.

Article pre-registration: https://osf.io/spdtb

Contents

Supplementary Materials 1: Changes to the pre-registered protocol........ 1
Supplementary Materials 2: detailed description of study measures........ 2
Supplementary materials 3: Univariable analyses........ 4
Supplementary Materials 4: Unweighted analyses........ 7
Supplementary Materials 5: Sensitivity Analyses for RQ3 (different cut-off values for MVPA change). 12

Supplementary Materials 1: Changes to the pre-registered protocol

The analysis protocol pre-registered on OSF prior to data inspection outlined analyses for research questions 3 and 4 involving unstratified multinomial analyses with change in MVPA and MSA as dependent variables. Specifically, the pre-registered protocol specified that we will use multinomial regression models to assess the correlates of a three-level dependent variables on change in (i) MVPA (increase by at least 20min/week, maintenance with change between 0 to 19min, and a decrease in MVPA by at least 20min/week) and in (ii) MSA (increase by at least 1 day/week, maintenance with change=0 days/week, and a decrease with change by at least 1 day/week). All the analyses were performed as per the original plan and can be accessed here: https://osf.io/2ujxq/.

Upon inspecting the data and the results a decision was made that this approach is not satisfactory as the group of maintainers of MVPA and MSA category included both inactive and active participants. This has motivated us to conduct and report the present analyses that stratified the sample based on pre-Covid-19 activity levels to identify meaningful changes.

Contact: Aleksandra Herbec a.herbec@ucl.ac.uk

For peer review only - http://bmjopen.bmj.com/site/about/guidelines.xhtml

Supplementary Materials 2: detailed description of study measures

Below are the details of the all the explanatory measures assessed at baseline that are relevant for the study. For the full wording of the baseline survey see: https://osf.io/bja7g/

Socio-demographic, health and wellbeing characteristics

Gender (female gender/other; prefer not to say (PNS) were treated as missing) was assessed by asking participants: "Which of the following best describes how you think of yourself?".

Ethnicity (white/non-white; PNS treated as missing) was assessed by asking participants: "What is your ethnic group?".

Education (high school education or higher; yes/no) was assessed by asking participants: "What is the highest level of education that you have completed?"

Employment (employed or self-employed; yes/no) and **furloughed/laid off** (yes/no) were assessed by asking participants: "What is your current MAIN occupation (during COVID-19)?".

Income (low-middle <50 000 GBP vs high ≥50 000 GBP vs PNS) was assessed by asking participants: "What was your household annual income BEFORE COVID-19? (do not include income of friends)".

Age was assessed as a continuous variable. For the analyses we used age in decades and entered it as a continuous variable. Where the continuous variable violated the linearity assumptions of the logistic regression models (as did its logarithmic and square root transformation), a 3-level categorical variable was created (≤34, 35-64, 65+).

Health behaviours and conditions

- **Body mass index, BMI** (normal and underweight ≤24.99/overweight 25-29.99/ obese ≥30/ unknown (included answers 'don't know' and PNS to height or weight variables)). BMI was calculated by dividing participants weight in kilogram by their squared height in meters and categorised into: normal weight and underweight. BMI is associated with both MVPA and MSA levels (Churilla et al., 2018).

- **Self-reports of any condition that limited their ability to engage in physical activity** (yes/no; PNS treated as missing). Participants were asked: "Do you now suffer from any condition that limits you from engaging in physical activity, including walking or doing housework?"

- **Self-perceived risk of Covid-19 to one's health** (minor or no risk/major, significant, moderate risk or 'don't know') was assessed with the question: "What risk does COVID-19 pose to your health?"

- **Smoking status** (current smokers vs not) was assessed using a single item: "Which statement about tobacco use and cigarette smoking best describes you?". Those responding 1 ("I smoke cigarettes (including hand-rolled) every day") and 2 ("I smoke cigarettes (including hand-rolled), but not every day") were classified as current smokers. Smoking is associated with physical activity levels (Kaczynski, Manske, Mannell, & Grewal, 2008).

Contact: Aleksandra Herbec a.herbec@ucl.ac.uk
For peer review only - http://bmjopen.bmj.com/site/about/guidelines.xhtml

- **Weekly alcohol consumption** since the Covid-19 pandemic has started was assessed by asking participants how many times they had a drink containing alcohol, with the categorical answers converted to weekly frequencies from 0 to 6.5 times per week (0=I have not had any alcohol after COVID-19 started, 0.25=monthly or less, 0.75=2 to 4 times a month, 2.5=2 to 3 times a week, 4.5=4 to 5 times a week, 6.5=6 or more times a week). In models where the continuous alcohol frequency variable violated the linearity assumptions of the logistic regression models (as did its logarithmic and square root transformation), weekly alcohol frequency was categorised into three levels (none/weekly or less often/more frequently than weekly). Alcohol drinking is associated with physical activity levels (Leasure, Neighbors, Henderson, & Young, 2015).

- **Deterioration in psychological wellbeing** (yes/no). Deterioration in psychological wellbeing was assessed on a five-point Likert scale (1=poor, 3=average, 5=excellent) by asking participants: "How would you rate the below aspects of your life? – Psychological wellbeing". The question was asked about the period before Covid-19 and since Covid-19. A change score was calculated by deducting the score before from since score. As the continuous variable violated the linearity assumptions of the logistic regression models (as did its logarithmic and square root transformation), participants were categorised into either deteriorated psychological wellbeing (change score < 0) versus maintained or improved psychological wellbeing (change score ≥ 0).

- **Isolation status** (total isolation/other) was assessed by asking which type of Covid-19 induced isolation participants were experiencing. Answers were dichotomised into indications of total isolation/quarantine ("not leaving the house for any reasons, not even to buy groceries or medications or to exercise") versus any other (some, general, or no isolation). Participants' perceived risk of Covid-19 to their health was dichotomised into 'no or minor risk perception' versus any other indication (moderate risk, significant, major or not known). It was hypothesised that perceived risk could affect physical activity, especially MVPA that may happen outdoors.

- **Access to space to exercise** was assessed using a single question on participants' access to spaces that allowed multiple answers, including for: „a garden or balcony big enough to exercise comfortably" and to "a public park/green space that is within a walking distance and open during Covid-19". The answers were dummy coded into separate binary variables (yes/no).

- **Living with children age 15 and younger** (yes/no) and **living with vulnerable persons** (any of: persons over the age of 70, persons in poor health versus, or persons who they believed may be vulnerable to Covid-19 for any reason) was assessed by asking participants: "Do you live with any of these persons below? (select all that apply)." It was hypothesised that living with these groups could limit some of the opportunities to exercise.

Contact: Aleksandra Herbec a.herbec@ucl.ac.uk
For peer review only - http://bmjopen.bmj.com/site/about/guidelines.xhtml

Supplementary materials 3: Univariable analyses.

Supplementary Table S3.1. (univariable) Research question 2 -Predictors of meeting both, MVPA only and MSA only in comparison to meeting WHO guidelines for neither MVPA nor MSA (reference, n=1178). Results from unadjusted models on weighted data with BH FDR correction of p-values (significant in bold).

	Both (n=388)				MVPA only (n=570)				MSA only (n=306)			
	OR	95% CI		*p*	OR	95% CI		*p*	OR	95% CI		*p*
Female	1.01	0.80	1.27	0.928	**0.62**	**0.51**	**0.76**	**<0.001**	0.93	0.72	1.19	0.546
Age ≤34[a]												
Age: 35-64	**0.57**	**0.43**	**0.75**	**<0.001**	1.08	0.82	1.42	0.604	**0.41**	**0.30**	**0.56**	**<0.001**
Age: 65+	**0.41**	**0.28**	**0.60**	**<0.001**	1.02	0.73	1.43	0.893	**0.58**	**0.40**	**0.84**	**0.004**
White ethnicity	0.77	0.52	1.12	0.166	1.53	1.02	2.30	0.040	**0.43**	**0.30**	**0.62**	**<0.001**
Household income ≥50.000												
Income: <50 000 GBP	**0.44**	**0.33**	**0.59**	**<0.001**	**0.66**	**0.50**	**0.85**	**0.002**	0.80	0.57	1.12	0.194
Income: Prefer not to say	**0.41**	**0.25**	**0.66**	**<0.001**	**0.50**	**0.32**	**0.77**	**0.002**	0.79	0.47	1.34	0.385
Education: Highschool or higher	**2.57**	**1.96**	**3.38**	**<0.001**	**1.34**	**1.09**	**1.65**	**0.006**	**2.41**	**1.79**	**3.24**	**<0.001**
Employed	1.14	0.90	1.43	0.272	1.20	0.98	1.46	0.077	1.05	0.81	1.35	0.714
Laid-off/Furloughed	0.95	0.67	1.35	0.784	1.01	0.75	1.36	0.972	1.04	0.72	1.51	0.839
Condition limiting PA	**0.24**	**0.16**	**0.37**	**<0.001**	**0.27**	**0.19**	**0.37**	**<0.001**	**0.64**	**0.46**	**0.89**	**0.009**
BMI ≤24												
BMI: 25-29.99	**0.62**	**0.48**	**0.81**	**<0.001**	0.86	0.68	1.08	0.191	**0.60**	**0.45**	**0.79**	**<0.001**
BMI: Obese: 30+	**0.31**	**0.22**	**0.43**	**<0.001**	**0.62**	**0.48**	**0.81**	**<0.001**	**0.27**	**0.19**	**0.40**	**<0.001**
Total isolation	**0.30**	**0.17**	**0.52**	**<0.001**	**0.17**	**0.10**	**0.31**	**<0.001**	0.67	0.43	1.05	0.079
Minor/no Covid-19 risk percept	**1.70**	**1.34**	**2.16**	**<0.001**	**1.39**	**1.12**	**1.72**	**0.002**	**1.37**	**1.05**	**1.79**	**0.019**
Living with children	0.99	0.73	1.33	0.925	0.81	0.61	1.06	0.121	0.70	0.49	1.01	0.056
Living with vulnerable	1.09	0.80	1.48	0.583	0.80	0.59	1.06	0.122	0.87	0.61	1.25	0.450
Access garden/balcony to exercise comfortably in	1.18	0.91	1.54	0.212	1.01	0.81	1.27	0.918	0.82	0.63	1.08	0.157
Access green space within walking distance	**1.70**	**1.33**	**2.16**	**<0.001**	**1.59**	**1.29**	**1.95**	**<0.001**	1.29	1.00	1.67	0.049
Smoker	**0.48**	**0.35**	**0.64**	**<0.001**	**0.72**	**0.57**	**0.91**	**0.006**	**0.64**	**0.47**	**0.87**	**0.004**
Alcohol drinking frequency, never (ref)												
Weekly or less	1.32	0.94	1.85	0.106	**1.50**	**1.12**	**2.02**	**0.006**	1.48	1.04	2.11	0.032
More than weekly	**1.47**	**1.09**	**1.98**	**0.012**	**1.50**	**1.15**	**1.95**	**0.003**	1.21	0.87	1.68	0.249
Deteriorated psychological wellbeing	**0.62**	**0.50**	**0.79**	**<0.001**	**0.71**	**0.58**	**0.87**	**0.001**	0.74	0.58	0.95	0.019
Meeting WHO PA rec (meeting neither=ref)												
Meeting both before	**17.58**	**12.53**	**24.65**	**<0.001**	**2.30**	**1.60**	**3.31**	**<0.001**	**4.03**	**2.72**	**5.99**	**<0.001**
Meeting only MVPA before	**4.53**	**3.13**	**6.55**	**<0.001**	**8.30**	**6.47**	**10.65**	**<0.001**	1.67	1.08	2.58	0.021
Meeting only MSA before	**7.72**	**5.27**	**11.32**	**<0.001**	0.86	0.52	1.42	0.549	**10.31**	**7.37**	**14.43**	**<0.001**

Contact: Aleksandra Herbec a.herbec@ucl.ac.uk

For peer review only - http://bmjopen.bmj.com/site/about/guidelines.xhtml

For peer review only

Supplementary Table S3.2. (univariable) Research question 3: Independent associations of change in MVPA. Segmented analyses of active sample (pre-Covid-19 MVPA activity ≥30min; predicting decrease by ≥20min) and less active/inactive sample (pre-Covid-19 MVPA activity <30min; predicting increase by ≥20min). Findings from unadjusted logistic regression models on weighted data and using BH FDR adjustment (significant in bold).

	Sample active (≥30min/week) pre-Covid-19 (weighted n=1857)				Sample inactive (<30min/week) pre-Covid-19 (weighted n=585)			
	Decrease (n=868) vs not				Increase (n=174) vs not			
	OR	95% CI		*p*	OR	95% CI		*p*
Female	**1.38**	**1.15**	**1.65**	**0.001**	1.13	0.79	1.62	0.496
Age (in decades)	0.94	0.88	0.99	0.024	**0.67**	**0.59**	**0.75**	**<0.001**
White ethnicity	**0.57**	**0.42**	**0.77**	**<0.001**	0.46	0.24	0.88	0.019
Household income ≥50.000								
Income: <50 000 GBP	**1.36**	**1.08**	**1.71**	**0.010**	**0.45**	**0.27**	**0.77**	**0.003**
Income: Prefer not to say	**1.59**	**1.09**	**2.33**	**0.017**	0.53	0.25	1.14	0.103
Education: Highschool or higher	**0.72**	**0.59**	**0.88**	**0.001**	**2.67**	**1.81**	**3.93**	**<0.001**
Employed	0.82	0.69	0.99	0.036	0.80	0.55	1.16	0.235
Laid-off/Furloughed	1.22	0.92	1.62	0.165	**2.16**	**1.36**	**3.43**	**0.001**
Condition limiting PA	**2.28**	**1.71**	**3.04**	**<0.001**	**0.27**	**0.16**	**0.43**	**<0.001**
BMI ≤24								
BMI: 25-29.99	1.01	0.82	1.23	0.961	1.04	0.69	1.55	0.863
BMI: Obese: 30+	0.99	0.78	1.27	0.945	0.56	0.34	0.90	0.016
Total isolation	**4.95**	**3.11**	**7.88**	**<0.001**	**0.22**	**0.10**	**0.49**	**<0.001**
Minor/no Covid-19 risk percept	0.94	0.77	1.14	0.503	**2.17**	**1.50**	**3.14**	**<0.001**
Living with children	0.89	0.70	1.13	0.319	1.37	0.82	2.30	0.225
Living with vulnerable	**0.72**	**0.56**	**0.93**	**0.012**	1.14	0.70	1.85	0.599
Access garden/balcony to exercise comfortably in	0.82	0.67	1.00	0.053	**0.61**	**0.42**	**0.89**	**0.011**
Access green space within walking distance	0.81	0.67	0.98	0.026	**1.66**	**1.16**	**2.37**	**0.006**
Smoker	0.94	0.75	1.17	0.576	1.15	0.79	1.68	0.454
Alcohol drinking frequency, never (ref)								
Weekly or less	**0.68**	**0.52**	**0.89**	**0.005**	1.19	0.73	1.96	0.483
More than weekly	**0.60**	**0.47**	**0.76**	**<0.001**	1.22	0.78	1.90	0.387
Deteriorated psychological wellbeing	**1.42**	**1.18**	**1.71**	**<0.001**	1.03	0.73	1.47	0.854

Adjusted for the time of enrolment and time when Covid-19 started to affect individuals in any way.

Contact: Aleksandra Herbec a.herbec@ucl.ac.uk

For peer review only

Supplementary Table S3.3 Research question 4: Independent associations of change in MSA. Segmented analyses of active sample (pre-Covid-19 MSA activity ≥1 day/week; predicting decrease by ≥1 day/week) and less inactive sample (pre-Covid-19 MSA activity 0 days/week; predicting increase by ≥1 day/week). Findings from unadjusted logistic regression models on weighted data and using BH FDR adjustment (significant in bold).

	Sample active (≥1 day/week) pre-Covid-19 (weighted n=985)				Sample inactive (0 days/week) pre-Covid-19 (weighted n=1456)			
	Decrease (n=444) vs not				Increase (n=239) vs not			
	OR	95% CI		*p*	OR	95% CI		*p*
Female	0.87	0.67	1.12	0.274	0.85	0.64	1.12	0.249
Age (in decades)	1.02	0.94	1.10	0.625	-	-	-	-
Age ≤34[a]								
Age: 35-64	-	-	-	-	**0.20**	**0.14**	**0.28**	**<0.001**
Age: 65+	-	-	-	-	**0.27**	**0.18**	**0.40**	**<0.001**
White ethnicity	0.80	0.57	1.13	0.210	**0.36**	**0.22**	**0.58**	**<0.001**
Household income ≥50.000								
Income: <50 000 GBP	1.01	0.75	1.36	0.953	**0.61**	**0.42**	**0.87**	**0.007**
Income: Prefer not to say	0.80	0.48	1.31	0.368	0.64	0.36	1.17	0.147
Education: Highschool or higher	0.74	0.55	1.00	0.051	**1.86**	**1.37**	**2.52**	**<0.001**
Employed	**1.60**	**1.24**	**2.06**	**<0.001**	1.01	0.76	1.33	0.951
Laid-off/Furloughed	0.99	0.67	1.45	0.954	1.33	0.91	1.96	0.141
Condition limiting PA	1.35	0.92	1.97	0.120	**0.43**	**0.27**	**0.67**	**<0.001**
BMI ≤24								
BMI: 25-29.99	**1.88**	**1.42**	**2.50**	**<0.001**	0.68	0.50	0.93	0.016
BMI: Obese: 30+	**3.20**	**2.18**	**4.72**	**<0.001**	**0.43**	**0.29**	**0.63**	**<0.001**
Total isolation	**2.05**	**1.22**	**3.45**	**0.007**	0.62	0.35	1.10	0.102
Minor/no Covid-19 risk percept	1.07	0.83	1.38	0.596	**1.61**	**1.20**	**2.15**	**0.001**
Living with children	0.83	0.60	1.15	0.266	1.44	0.95	2.18	0.086
Living with vulnerable	1.13	0.79	1.63	0.498	1.01	0.69	1.47	0.971
Access garden/balcony to exercise comfortably in	0.90	0.67	1.19	0.453	0.74	0.55	0.99	0.043
Access green space within walking distance	0.94	0.72	1.22	0.621	1.12	0.85	1.49	0.420
Smoker	0.65	0.46	0.90	0.010	1.00	0.74	1.36	0.995
Alcohol drinking frequency, never (ref)								
Weekly or less	0.88	0.61	1.27	0.508	1.50	1.00	2.27	0.051
More than weekly	0.93	0.67	1.30	0.677	1.40	0.97	2.03	0.072
Deteriorated psychological wellbeing	1.37	1.06	1.77	0.014	0.72	0.55	0.95	0.021

Adjusted for the time of enrolment and time when Covid-19 started to affect individuals in any way.

[a]Age was categorised in models where the continuous predictor violated the linearity assumption.

Contact: Aleksandra Herbec a.herbec@ucl.ac.uk

For peer review only - http://bmjopen.bmj.com/site/about/guidelines.xhtml

For peer review only

Supplementary Materials 4: Unweighted analyses

Supplementary Table S4.1. (unweighted data) Sample characteristics % (N).

	Included sample (2657)	Excluded sample (336)	*p*
Female	68.9 (1831)	68.1 (224)	0.760
Age; M (SD)	48.4 (15.2)	43.7 (16.7)	<0.001
White ethnicity	94.4 (2507)	91.1 (297)	0.020
Household income ≥50.000	90.9 (993)	9.1 (100)	<0.001
Income: <50 000 GBP	54.5 (1447)	55.5 (186)	
Income: Prefer not to say	8.2 (217)	14.6 (49)	
Education: Highschool or higher	86.8 (2307)	85.7 (288)	0.571
Employed	56.1 (1490)	49.1 (164)	0.016
Laid-off/Furloughed	9.8 (261)	12.3 (41)	0.161
Condition limiting PA	13.2 (351)	19.6 (54)	0.003
BMI ≤24	49.1 (1305)	23.8 (62)	<0.001
BMI: 25-29.99	31.3 (832)	15.8 (41)	
BMI: Obese: 30+	19.6 (520)	9.2 (24)	
Total isolation	7.2 (190)	7.2 (21)	0.955
Minor/no Covid-19 risk percept	37.5 (996)	42.4 (123)	0.101
Living with children	19.6 (520)	19.0 (64)	0.820
Living with vulnerable	14.8 (394)	16.1 (54)	0.547
Access garden/balcony	71.9 (1911)	63.1 (212)	0.001
Access green space	63.5 (1687)	57.1 (192)	0.023
Time affected up till mid March	49.5 (1316)	55.1 (185)	0.056
Enrolled from 1st June (ref)	5.2 (139)	4.8 (16)	0.479
Enrolled up until 15th May	48.9 (1298)	45.8 (154)	
Enrolled second half of May	45.9 (1220)	49.4 (166)	
Smoker	17.7 (470)	25.6 (86)	<0.001
Alcohol drinking frequency, never (ref)	17.0 (453)	19.6 (66)	0.092
Weekly or less	27.5 (732)	31.3 (105)	
More than weekly	55.4 (1472)	49.1 (165)	
Deteriorated psychological wellbeing	55.1 (1465)	50.2 (117)	0.148
Meeting WHO PA rec (meeting both=ref)	17.9 (475)	14.9 (26)	0.332
Meeting only MVPA before	22.8 (605)	23.0 (40)	
Meeting only MSA before	14.9 (395)	11.5 (20)	
Meeting neither before	44.5 (1182)	50.6 (88)	

Contact: Aleksandra Herbec a.herbec@ucl.ac.uk

For peer review only

Supplementary Table S4.2. (unweighted data) Aerobic physical activity (MVPA) and strength training (MSA) before to after the Covid-19 pandemic has started to affect individuals in the UK (up until 14th June 2020).

	Before Covid-19[1]	Since Covid-19[1]
Inactive (0 sessions MVPA and 0 days MSA) % (N)	14.1 (375)	16.0 (425)
MVPA 0 sessions % (N)	15.9 (422)	18.5 (491)
MVPA		
MVPA min/week among active; Median (IQR)	135.0 (180.0)	150.0 (240.0)
Mean (SD)	215.3 (363.4)	227.0 (341.7)
Mean (SD) replacing extremes with 840 min/week	191.8 (186.9)	206.9 (193.7)
MVPA min/week among entire sample; Median (IQR)	120.0 (185.0)	120.0 (215.0)
Mean (SD)	184.9 (345.0)	190.7 (324.1)
Mean (SD) replacing extremes with 840 min/week	164.7 (185.6)	173.8 (193.1)
MSA Median (IQR)	0 (2)	0 (2)
MSA % (N)		
0 das/week	53.0 (1408)	54.0 (1435)
1 day/week	14.3 (379)	11.7 (311)
2 days/week	14.6 (387)	10.5 (280)
3 days/week	10.4 (277)	9.6 (254)
4+ days/week	7.8 (206)	14.2 (377)
Meet guidelines % (N)		
Neither MVPA nor MSA	44.5 (1182)	42.0 (1117)
Meet MVPA (≥150min/week) only	22.8 (605)	23.7 (629)
Meet MSA (≥2 days/week) only	14.9 (395)	14.7 (390)
Meets both MVPA and MSA	17.9 (475)	19.6 (521)
Change in form of exercise % (N)		
None of the same		13.2 (350)
Some of the same		29.1 (774)
About half the same		8.8 (233)
Mostly the same		23.4 (622)
Exactly the same		10.0 (267)

Contact: Aleksandra Herbec a.herbec@ucl.ac.uk

For peer review only - http://bmjopen.bmj.com/site/about/guidelines.xhtml

For peer review only

Supplementary Table S4.3. (unweighted data) Research question 2 – Factors associated with meeting both, MVPA only and MSA only in comparison to meeting WHO guidelines for neither MVPA nor MSA (reference, n=1117) after the Covid-19 pandemic has started to affect individuals in the UK (up until June 2020[1]). Results from fully adjusted models on unweighted data with BH FDR correction of p-values (significant in bold).

	Both (n=521)				MVPA only (n=629)				MSA only (n=390)			
Characteristics and associations	aOR	95% CI		*p*	aOR	95% CI		*p*	aOR	95% CI		*p*
Female	1.17	0.89	1.52	0.256	0.93	0.73	1.18	0.546	1.16	0.87	1.53	0.308
Age ≤34[a]												
Age: 35-64	0.92	0.67	1.25	0.589	**1.49**	**1.09**	**2.05**	**0.013**	**0.59**	**0.43**	**0.82**	**0.001**
Age: 65+	0.64	0.40	1.03	0.067	**1.80**	**1.17**	**2.75**	**0.007**	0.90	0.57	1.44	0.664
White ethnicity	1.36	0.81	2.27	0.245	1.79	1.01	3.15	0.044	0.67	0.42	1.08	0.099
Household income ≥50.000												
Income: <50 000 GBP	**0.58**	**0.44**	**0.76**	**<0.001**	**0.70**	**0.55**	**0.90**	**0.006**	1.00	0.75	1.33	0.976
Income: Prefer not to say	**0.55**	**0.34**	**0.89**	**0.014**	0.76	0.49	1.17	0.214	0.90	0.54	1.48	0.667
Education: Highschool or higher	1.38	0.92	2.09	0.121	1.20	0.86	1.67	0.293	**1.86**	**1.19**	**2.90**	**0.007**
Employed	0.78	0.58	1.05	0.102	1.11	0.85	1.47	0.442	1.22	0.89	1.68	0.212
Laid-off/Furloughed	1.00	0.64	1.55	0.997	1.07	0.70	1.62	0.759	1.33	0.84	2.13	0.227
Condition limiting PA	**0.42**	**0.27**	**0.65**	**<0.001**	**0.36**	**0.24**	**0.54**	**<0.001**	0.78	0.53	1.16	0.219
BMI ≤24												
BMI: 25-29.99	**0.61**	**0.46**	**0.80**	**<0.001**	0.80	0.62	1.02	0.076	**0.66**	**0.49**	**0.88**	**0.005**
BMI: Obese: 30+	**0.35**	**0.24**	**0.51**	**<0.001**	**0.69**	**0.51**	**0.93**	**0.014**	**0.43**	**0.30**	**0.62**	**<0.001**
Total isolation	**0.39**	**0.22**	**0.69**	**0.001**	**0.22**	**0.12**	**0.41**	**<0.001**	0.84	0.52	1.38	0.498
Minor/no Covid-19 risk percept	0.82	0.64	1.07	0.147	0.92	0.72	1.17	0.476	0.90	0.68	1.20	0.477
Living with children	0.79	0.58	1.07	0.130	**0.56**	**0.42**	**0.76**	**<0.001**	0.89	0.64	1.24	0.497
Living with vulnerable	1.14	0.81	1.60	0.457	1.07	0.79	1.46	0.662	0.94	0.65	1.35	0.721
Access garden/balcony to exercise comfortably in	1.09	0.82	1.44	0.562	1.00	0.78	1.29	0.994	0.98	0.74	1.31	0.901
Access green space within walking distance	1.17	0.90	1.51	0.243	1.23	0.97	1.56	0.081	1.16	0.89	1.53	0.278
Smoker	**0.56**	**0.40**	**0.79**	**0.001**	**0.69**	**0.52**	**0.93**	**0.016**	**0.65**	**0.46**	**0.92**	**0.016**
Alcohol drinking frequency, never (ref)												
Weekly or less	1.24	0.84	1.82	0.279	1.16	0.82	1.63	0.405	1.07	0.72	1.58	0.745
More than weekly	1.25	0.88	1.78	0.221	1.14	0.83	1.56	0.412	1.02	0.71	1.47	0.923
Deteriorated psychological wellbeing	**0.61**	**0.48**	**0.78**	**<0.001**	0.78	0.63	0.98	0.029	**0.65**	**0.51**	**0.84**	**0.001**
Meeting WHO PA rec (meeting neither=ref)												
Meeting both before	**14.58**	**10.48**	**20.30**	**<0.001**	**2.47**	**1.74**	**3.52**	**<0.001**	**3.53**	**2.44**	**5.10**	**<0.001**
Meeting only MVPA before	**3.31**	**2.35**	**4.66**	**<0.001**	**5.86**	**4.54**	**7.55**	**<0.001**	0.84	0.54	1.31	0.445
Meeting only MSA before	**4.98**	**3.49**	**7.11**	**<0.001**	0.79	0.51	1.24	0.303	**6.59**	**4.80**	**9.06**	**<0.001**

Models adjusted for the time of enrolment and time when Covid-19 started to affect individuals in any way.

[1] Covering the period of the strictest first lockdown in the UK

Contact: Aleksandra Herbec a.herbec@ucl.ac.uk

For peer review only

Supplementary Table S4.4. Research question 3: Independent associations of change in MVPA from before to after the Covid-19 pandemic has started to affect individuals in the UK (up until 14th June 2020[2]). Segmented analyses of active sample (pre-Covid-19 MVPA activity ≥30min; predicting decrease by ≥20min) and less active/inactive sample (pre-Covid-19 MVPA activity <30min; predicting increase by ≥20min). Findings from fully adjusted logistic regression models on unweighted data and using BH FDR adjustment (significant in bold).

Characteristics and associations	Sample active (≥30min/week) pre-Covid-19 (unweighted n=2132)				Sample inactive (<30min/week) pre-Covid-19 (unweighted n=525)			
	Decrease (n=905) vs not				Increase (n=195) vs not			
	aOR	95% CI		*p*	aOR	95% CI		*p*
Female	1.05	0.86	1.27	0.660	1.12	0.73	1.74	0.605
Age (in decades)	**0.91**	**0.85**	**0.98**	**0.011**	**0.80**	**0.68**	**0.94**	**0.007**
White ethnicity	**0.52**	**0.35**	**0.76**	**0.001**	0.80	0.30	2.15	0.656
Household income ≥50.000								
Income: <50 000 GBP	**1.41**	**1.15**	**1.73**	**0.001**	0.95	0.59	1.53	0.829
Income: Prefer not to say	1.31	0.92	1.87	0.130	0.72	0.32	1.65	0.441
Education: Highschool or higher	0.73	0.54	0.98	0.036	1.87	1.07	3.26	0.028
Employed	0.96	0.77	1.18	0.682	0.70	0.43	1.13	0.140
Laid-off/Furloughed	0.95	0.68	1.34	0.782	1.51	0.77	2.93	0.227
Condition limiting PA	**1.87**	**1.37**	**2.55**	**<0.001**	**0.47**	**0.27**	**0.83**	**0.010**
BMI ≤24								
BMI: 25-29.99	1.08	0.88	1.33	0.456	1.11	0.69	1.79	0.665
BMI: Obese: 30+	0.97	0.75	1.26	0.833	0.71	0.42	1.19	0.195
Total isolation	**2.91**	**1.89**	**4.46**	**<0.001**	**0.22**	**0.09**	**0.54**	**0.001**
Minor/no Covid-19 risk percept	1.21	0.99	1.47	0.067	1.19	0.76	1.87	0.441
Living with children	0.80	0.64	1.01	0.061	0.86	0.52	1.44	0.570
Living with vulnerable	0.92	0.71	1.20	0.551	0.83	0.49	1.43	0.511
Access garden/balcony to exercise comfortably in	**0.74**	**0.60**	**0.91**	**0.005**	0.96	0.61	1.49	0.847
Access green space within walking distance	0.95	0.78	1.15	0.589	1.45	0.96	2.19	0.077
Smoker	0.88	0.68	1.13	0.311	1.70	1.05	2.74	0.030
Alcohol drinking frequency, never (ref)								
Weekly or less	1.08	0.81	1.44	0.605	0.96	0.54	1.71	0.890
More than weekly	0.89	0.68	1.16	0.378	1.24	0.75	2.06	0.399
Deteriorated psychological wellbeing	**1.40**	**1.16**	**1.68**	**<0.001**	1.12	0.74	1.67	0.597

Models adjusted for the time of enrolment and time when Covid-19 started to affect individuals in any way.

[2] Covering the period of the strictest first lockdown in the UK

Contact: Aleksandra Herbec a.herbec@ucl.ac.uk

For peer review only

Supplementary Table S4.5. (unweighted data). Research question 4: Independent associations of change in MSA from before to after the Covid-19 pandemic has started to affect individuals in the UK (up until 14th June 2020[3]). Segmented analyses of active sample (pre-Covid-19 MSA activity ≥1 day/week; predicting decrease by ≥1 day/week) and less inactive sample (pre-Covid-19 MSA activity 0 days/week; predicting increase by ≥1 day/week). Findings from fully adjusted logistic regression models on unweighted data and using BH FDR adjustment (significant in bold).

	Sample active (≥1 day/week) pre-Covid-19 (unweighted n=1249)				Sample inactive (0 days/week) pre-Covid-19 (unweighted n=1408)			
	Decrease (n=534) vs not				Increase (n=284) vs not			
Characteristics and associations	aOR	95% CI		*p*	aOR	95% CI		*p*
Female	0.83	0.64	1.08	0.160	1.216	0.899	1.647	0.205
Age (in decades)	1.03	0.94	1.12	0.579	-	-	-	-
Age ≤34[a]								
Age: 35-64	-	-	-	-	**0.353**	**0.248**	**0.502**	**<0.001**
Age: 65+	-	-	-	-	**0.305**	**0.178**	**0.524**	**<0.001**
White ethnicity	0.89	0.57	1.39	0.611	0.553	0.298	1.024	0.060
Household income ≥50.000								
Income: <50 000 GBP	1.25	0.96	1.64	0.096	**0.678**	**0.496**	**0.928**	**0.015**
Income: Prefer not to say	1.39	0.88	2.18	0.155	0.480	0.258	0.893	0.020
Education: Highschool or higher	0.96	0.62	1.49	0.863	1.310	0.837	2.051	0.237
Employed	**1.49**	**1.12**	**1.97**	**0.005**	0.900	0.634	1.278	0.557
Laid-off/Furloughed	1.18	0.75	1.85	0.467	1.104	0.676	1.803	0.694
Condition limiting PA	1.08	0.72	1.62	0.708	0.664	0.409	1.078	0.097
BMI ≤24								
BMI: 25-29.99	**1.63**	**1.24**	**2.13**	**<0.001**	0.846	0.613	1.168	0.310
BMI: Obese: 30+	**2.50**	**1.73**	**3.60**	**<0.001**	0.707	0.484	1.031	0.072
Total isolation	**2.03**	**1.18**	**3.50**	**0.011**	0.861	0.481	1.543	0.615
Minor/no Covid-19 risk percept	1.18	0.91	1.52	0.216	1.045	0.769	1.421	0.777
Living with children	1.04	0.77	1.41	0.784	0.708	0.503	0.997	0.048
Living with vulnerable	1.11	0.78	1.59	0.553	0.990	0.677	1.446	0.957
Access garden/balcony to exercise comfortably in	0.86	0.65	1.13	0.275	0.765	0.562	1.040	0.088
Access green space within walking distance	0.95	0.74	1.23	0.713	1.102	0.816	1.489	0.527
Smoker	**0.61**	**0.43**	**0.87**	**0.007**	1.096	0.772	1.557	0.608
Alcohol drinking frequency, never (ref)								
Weekly or less	1.01	0.69	1.49	0.943	1.192	0.776	1.833	0.422
More than weekly	0.99	0.69	1.42	0.951	1.124	0.755	1.672	0.565
Deteriorated psychological wellbeing	**1.38**	**1.09**	**1.76**	**0.008**	**0.643**	**0.488**	**0.848**	**0.002**

Models adjusted for the time of enrolment and time when Covid-19 started to affect individuals in any way.
[a]Age was categorised in models where the continuous predictor violated the linearity assumption.

[3] Covering the period of the strictest first lockdown in the UK

Contact: Aleksandra Herbec a.herbec@ucl.ac.uk

For peer review only

Supplementary Materials 5: Sensitivity Analyses for RQ3 (different cut-off values for MVPA change).

Supplementary Table S5.1. Predicting MVPA decrease of at least 15 min in the active sample from before to after the Covid-19 pandemic has started to affect individuals in the UK (up until 14th June 2020). (fully adjusted model, weighted data).

Characteristics and associations	**aOR**	**95% CI**		***p***
Female	1.232	1.010	1.503	.039
Age (in decades)	**.914**	**.849**	**.983**	**.015**
White ethnicity	**.649**	**.464**	**.909**	**.012**
Household income ≥50.000	1.0			
Income: <50 000 GBP	1.272	.986	1.641	.064
Income: Prefer not to say	1.182	.783	1.783	.427
Education: Highschool or higher	**.754**	**.602**	**.944**	**.014**
Employed	.841	.677	1.045	.118
Laid-off/Furloughed	1.025	.738	1.423	.884
Condition limiting PA	**1.732**	**1.260**	**2.381**	**.001**
BMI ≤24	1.0			
BMI: 25-29.99	1.025	.822	1.280	.824
BMI: Obese: 30+	.875	.667	1.147	.334
Total isolation	**3.682**	**2.252**	**6.020**	**.000**
Minor/no Covid-19 risk percept	.984	.791	1.224	.882
Living with children	.858	.661	1.114	.251
Living with vulnerable	.801	.607	1.058	.118
Access garden/balcony to exercise comfortably in	.773	.618	.968	.025
Access green space within walking distance	.813	.662	.998	.048
Smoker	1.106	.861	1.420	.432
Alcohol drinking frequency, never (ref)	1.0			
Weekly or less	.790	.596	1.049	.103
More than weekly	.772	.594	1.003	.053
Deteriorated psychological wellbeing	**1.422**	**1.170**	**1.728**	**.000**

Model was adjusted for all the variable sin the table as well as the time of enrolment and time when Covid-19 started to affect individuals in any way. Significant results are in bold.

Contact: Aleksandra Herbec a.herbec@ucl.ac.uk
For peer review only - http://bmjopen.bmj.com/site/about/guidelines.xhtml

Supplementary Table S5.2. Predicting MVPA increase of at least 15 min in the active sample from before to after the Covid-19 pandemic has started to affect individuals in the UK (up until 14th June 2020; fully adjusted model, weighted data).

Characteristics and associations	**aOR**	**95% CI**		**p**
Female	1.513	1.003	2.282	.048
Age (in decades)	**.750**	**.642**	**.876**	**.000**
White ethnicity	.564	.249	1.276	.169
Household income ≥50.000	1.0			
Income: <50 000 GBP	.633	.339	1.182	.152
Income: Prefer not to say	.663	.271	1.620	.367
Education: Highschool or higher	1.700	1.078	2.683	.023
Employed	.846	.526	1.362	.492
Laid-off/Furloughed	1.380	.762	2.498	.288
Condition limiting PA	**.445**	**.249**	**.795**	**.006**
BMI ≤24	1.0			
BMI: 25-29.99	1.640	1.014	2.652	.044
BMI: Obese: 30+	.778	.451	1.343	.368
Total isolation	.395	.172	.907	.029
Minor/no Covid-19 risk percept	1.349	.857	2.122	.196
Living with children	1.600	.917	2.793	.098
Living with vulnerable	.872	.505	1.508	.624
Access garden/balcony to exercise comfortably in	.730	.469	1.136	.163
Access green space within walking distance	.986	.656	1.481	.944
Smoker	1.491	.963	2.309	.073
Alcohol drinking frequency, never (ref)	1.0			
Weekly or less	.719	.407	1.272	.257
More than weekly	.941	.567	1.563	.814
Deteriorated psychological wellbeing	1.275	.850	1.913	.241

Model was adjusted for all the variable sin the table as well as the time of enrolment and time when Covid-19 started to affect individuals in any way. Significant results are in bold.

Contact: Aleksandra Herbec a.herbec@ucl.ac.uk

For peer review only - http://bmjopen.bmj.com/site/about/guidelines.xhtml

Supplementary Table S5.3. Predicting MVPA decrease of at least 30 min in the active sample (fully adjusted model, weighted data).

Characteristics and associations	aOR	95% CI		p
Female	1.207	.989	1.474	.064
Age (in decades)	**.904**	**.841**	**.973**	**.007**
White ethnicity	**.618**	**.441**	**.865**	**.005**
Household income ≥50.000	1.0			
Income: <50 000 GBP	1.356	1.049	1.753	.020
Income: Prefer not to say	1.276	.845	1.927	.246
Education: Highschool or higher	**.741**	**.592**	**.928**	**.009**
Employed	.853	.686	1.061	.153
Laid-off/Furloughed	1.037	.747	1.441	.827
Condition limiting PA	**1.672**	**1.219**	**2.293**	**.001**
BMI ≤24	1.0			
BMI: 25-29.99	1.047	.838	1.308	.684
BMI: Obese: 30+	.880	.671	1.155	.357
Total isolation	**3.426**	**2.130**	**5.511**	**.000**
Minor/no Covid-19 risk percept	.978	.785	1.218	.842
Living with children	.837	.644	1.087	.182
Living with vulnerable	.756	.572	.998	.048
Access garden/balcony to exercise comfortably in	**.744**	**.594**	**.932**	**.010**
Access green space within walking distance	.820	.667	1.008	.059
Smoker	1.097	.854	1.410	.468
Alcohol drinking frequency, never (ref)	1.0			
Weekly or less	.753	.567	.999	.049
More than weekly	.752	.578	.977	.033
Deteriorated psychological wellbeing	**1.403**	**1.154**	**1.706**	**.001**

Model was adjusted for all the variable sin the table as well as the time of enrolment and time when Covid-19 started to affect individuals in any way. Significant results are in bold.

Contact: Aleksandra Herbec a.herbec@ucl.ac.uk

For peer review only - http://bmjopen.bmj.com/site/about/guidelines.xhtml

Supplementary Table S5.4. Predicting MVPA increase of at least 30 min in the inactive sample (fully adjusted model, weighted data).

Characteristics and associations	**aOR**	**95% CI**		**p**
Female	**1.877**	**1.213**	**2.902**	**.005**
Age (in decades)	**.707**	**.599**	**.836**	**.000**
White ethnicity	1.391	.575	3.363	.464
Household income ≥50.000	1.0			
Income: <50 000 GBP	**.394**	**.205**	**.757**	**.005**
Income: Prefer not to say	**.208**	**.077**	**.560**	**.002**
Education: Highschool or higher	**2.116**	**1.296**	**3.456**	**.003**
Employed	**.516**	**.309**	**.861**	**.011**
Laid-off/Furloughed	1.380	.757	2.517	.293
Condition limiting PA	.478	.257	.887	.019
BMI ≤24	1.0			
BMI: 25-29.99	1.511	.917	2.490	.105
BMI: Obese: 30+	.616	.341	1.113	.108
Total isolation	.332	.133	.827	.018
Minor/no Covid-19 risk percept	1.001	.620	1.616	.997
Living with children	**2.290**	**1.225**	**4.283**	**.009**
Living with vulnerable	.936	.519	1.687	.825
Access garden/balcony to exercise comfortably in	.764	.482	1.209	.250
Access green space within walking distance	.960	.622	1.481	.853
Smoker	.979	.622	1.542	.926
Alcohol drinking frequency, never (ref)	1.0			
Weekly or less	1.028	.555	1.904	.931
More than weekly	1.234	.708	2.152	.459
Deteriorated psychological wellbeing	.888	.579	1.362	.585

Model was adjusted for all the variable sin the table as well as the time of enrolment and time when Covid-19 started to affect individuals in any way. Significant results are in bold.

Contact: Aleksandra Herbec a.herbec@ucl.ac.uk

For peer review only - http://bmjopen.bmj.com/site/about/guidelines.xhtml

Reporting checklist for cross sectional study.

Based on the STROBE cross sectional guidelines.

Instructions to authors

Complete this checklist by entering the page numbers from your manuscript where readers will find each of the items listed below.

Your article may not currently address all the items on the checklist. Please modify your text to include the missing information. If you are certain that an item does not apply, please write "n/a" and provide a short explanation.

Upload your completed checklist as an extra file when you submit to a journal.

In your methods section, say that you used the STROBE cross sectionalreporting guidelines, and cite them as:

von Elm E, Altman DG, Egger M, Pocock SJ, Gotzsche PC, Vandenbroucke JP. The Strengthening the Reporting of Observational Studies in Epidemiology (STROBE) Statement: guidelines for reporting observational studies.

For peer review only

		Reporting Item	Page Number
**Title and abstract**			
Title	#1a	Indicate the study's design with a commonly used term in the title or the abstract	2
Abstract	#1b	Provide in the abstract an informative and balanced	2

		summary of what was done and what was found	
**Introduction**			
Background / rationale	#2	Explain the scientific background and rationale for the investigation being reported	4
Objectives	#3	State specific objectives, including any prespecified hypotheses	4
**Methods**			
Study design	#4	Present key elements of study design early in the paper	5-6
Setting	#5	Describe the setting, locations, and relevant dates, including periods of recruitment, exposure, follow-up, and data collection	5
Eligibility criteria	#6a	Give the eligibility criteria, and the sources and methods of selection of participants.	5
	#7	Clearly define all outcomes, exposures, predictors, potential confounders, and effect modifiers. Give diagnostic criteria, if applicable	5-6, Supplement 2
Data sources / measurement	#8	For each variable of interest give sources of data and details of methods of assessment (measurement). Describe comparability of assessment methods if there is more than one group. Give information separately for for exposed and unexposed groups if applicable.	5-6
Bias	#9	Describe any efforts to address potential sources of bias	7

For peer review only

Study size	#10	Explain how the study size was arrived at	5, 7
Quantitative variables	#11	Explain how quantitative variables were handled in the analyses. If applicable, describe which groupings were chosen, and why	5-7
Statistical methods	#12a	Describe all statistical methods, including those used to control for confounding	7
Statistical methods	#12b	Describe any methods used to examine subgroups and interactions	7
Statistical methods	#12c	Explain how missing data were addressed	5-7, Supplement 2
Statistical methods	#12d	If applicable, describe analytical methods taking account of sampling strategy	7
Statistical methods	#12e	Describe any sensitivity analyses	7
**Results**			
Participants	#13a	Report numbers of individuals at each stage of study—eg numbers potentially eligible, examined for eligibility, confirmed eligible, included in the study, completing follow-up, and analysed. Give information separately for for exposed and unexposed groups if applicable.	7
Participants	#13b	Give reasons for non-participation at each stage	7

For peer review only

Participants	#13c	Consider use of a flow diagram	N/A
Descriptive data	#14a	Give characteristics of study participants (eg demographic, clinical, social) and information on exposures and potential confounders. Give information separately for exposed and unexposed groups if applicable.	7, Table 1
Descriptive data	#14b	Indicate number of participants with missing data for each variable of interest	Table 1
Outcome data	#15	Report numbers of outcome events or summary measures. Give information separately for exposed and unexposed groups if applicable.	7-8 Tables
Main results	#16a	Give unadjusted estimates and, if applicable, confounder-adjusted estimates and their precision (eg, 95% confidence interval). Make clear which confounders were adjusted for and why they were included	7-8 Tables
Main results	#16b	Report category boundaries when continuous variables were categorized	5-8
Main results	#16c	If relevant, consider translating estimates of relative risk into absolute risk for a meaningful time period	N/A
Other analyses	#17	Report other analyses done—e.g., analyses of subgroups and interactions, and sensitivity analyses	7, Suplements

Discussion

Key results	#18	Summarise key results with reference to study objectives	8-10

For peer review only

Limitations	#19	Discuss limitations of the study, taking into account sources of potential bias or imprecision. Discuss both direction and magnitude of any potential bias.	10
Interpretation	#20	Give a cautious overall interpretation considering objectives, limitations, multiplicity of analyses, results from similar studies, and other relevant evidence.	10
Generalisability	#21	Discuss the generalisability (external validity) of the study results	10

Other Information

Funding	#22	Give the source of funding and the role of the funders for the present study and, if applicable, for the original study on which the present article is based	11

None The STROBE checklist is distributed under the terms of the Creative Commons Attribution License CC-BY. This checklist can be completed online using https://www.goodreports.org/, a tool made by the EQUATOR Network in collaboration with Penelope.ai

For peer review only
